# Effect of backbone flexibility on covalent template-directed synthesis of linear oligomers†

**DOI:** 10.1039/d2ob01627c

**Published:** 2022-10-13

**Authors:** Diego Núñez-Villanueva, Christopher A. Hunter

**Affiliations:** Yusuf Hamied Department of Chemistry, University of Cambridge Lensfield Road Cambridge CB2 1EW UK herchelsmith.orgchem@ch.cam.ac.uk

## Abstract

Covalent template-directed synthesis can be used to replicate synthetic oligomers, but success depends critically on the conformational properties of the backbone. Here we investigate how the choice of monomer building block affects the flexibility of the backbone and in turn the efficiency of the replication process for a series of different triazole oligomers. Two competing reaction pathways were identified for monomers attached to a template, resulting in the formation of either macrocyclic or linear products. For flexible backbones, macrocycles and linear oligomers are formed at similar rates, but a more rigid backbone gave exclusively the linear product. The experimental results are consistent with ring strain calculations using molecular mechanics: products with low ring strain (20–30 kJ mol^−1^) formed rapidly, and products with high ring strain (>100 kJ mol^−1^) were not observed. Template-directed replication of linear oligomers requires monomers that rigid enough to prevent the formation of undesired macrocycles, but not so rigid that the linear templating pathway leading to the duplex is inhibited. Molecular mechanics calculations of ring strain provide a straightforward tool for assessing the flexibility of potential backbones and the viability different monomer designs before embarking on synthesis.

## Abstract 2

Conformational flexibility is important in selection of monomer building blocks used in template-directed replication of linear oligomers. The ring strain calculated for competing macrocyclisation reactions provides a useful design criterion.

## Graphical abstract

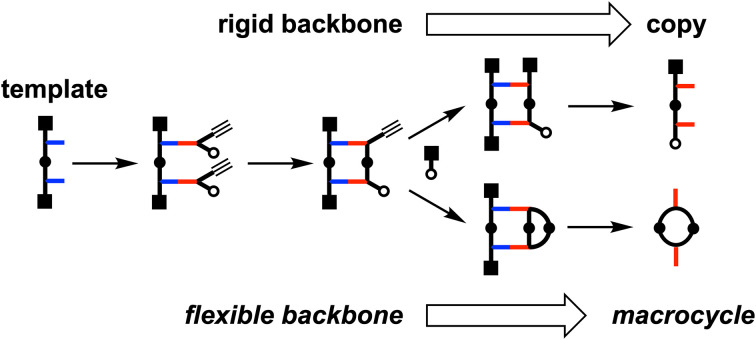

## Introduction

Nucleic acids encode functional biological information as a sequence of monomer units assembled into a linear polymer, which is replicated and translated into amino acid polymers *via* template-directed synthesis.^1^ The evolution of living organisms relies on this sequence information transfer process. Molecular evolution has been used to find novel functional biopolymers and to tailor proteins for therapeutic or manufacturing applications.^2–7^ Nevertheless, the chemical space accessible with current methods is limited to nucleic acids and proteins.^8–10^ The vast regions of chemical space constituted by synthetic information-containing polymers cannot therefore be targeted using nucleic acid-based replication.^11,12^ Although template-directed polymer synthesis has been demonstrated for polydisperse mixtures of synthetic polymers, these approaches are limited to homopolymers that do not contain sequence information.^13–18^ The synthesis of oligomers that contain sequences of different monomer units currently relies on solid phase methods.^19,20^ The development of an efficient method for transferring sequence information between synthetic polymers would open up the use of evolutionary methods to new molecular systems not found in biology.

**Fig. 1:**
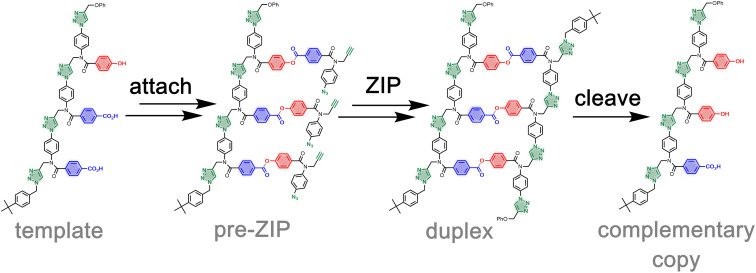
Sequence information transfer using covalent template-directed synthesis. In the attach step, complementary monomers are loaded onto the template using phenol–benzoic acid ester base-pairing chemistry. In the ZIP step, intramolecular CuAAC reactions lead to oligomerization of monomers on the template followed by capping of the chain ends. In the cleave step, hydrolysis of the ester bonds connecting the new oligomer to the template regenerates the template and releases the complementary copy strand.

We have recently reported a method for replicating synthetic oligomers using kinetically inert covalent base-pairs (Fig. 1).^21^ The information is encoded as a sequence of phenol and benzoic acid recognition units. Covalent base-pairs are formed by ester bond formation between phenol and benzoic acid and cleaved by hydrolysis. In the first step of the replication process shown in Fig. 1, polymerisable monomers equipped with an alkyne and an azide are attached to complementary bases on a mixed sequence template *via* a series of protection-coupling–deprotection-coupling reactions, to give the pre-ZIP intermediate. Intramolecular oligomerization *via* copper catalysed alkyne azide cycloaddition (CuAAC) in the presence of an end-capping azide leads to the duplex, which is cleaved to release the complementary copy and regenerate the template.^22,23^ We have shown that the transfer of information can be programmed by varying the chemical connectivity in the covalent base-pair using traceless linkers.^24^ The linkers allow either direct or reciprocal replication and provide a method for controlled mutation.^25,26^

**Fig. 2:**
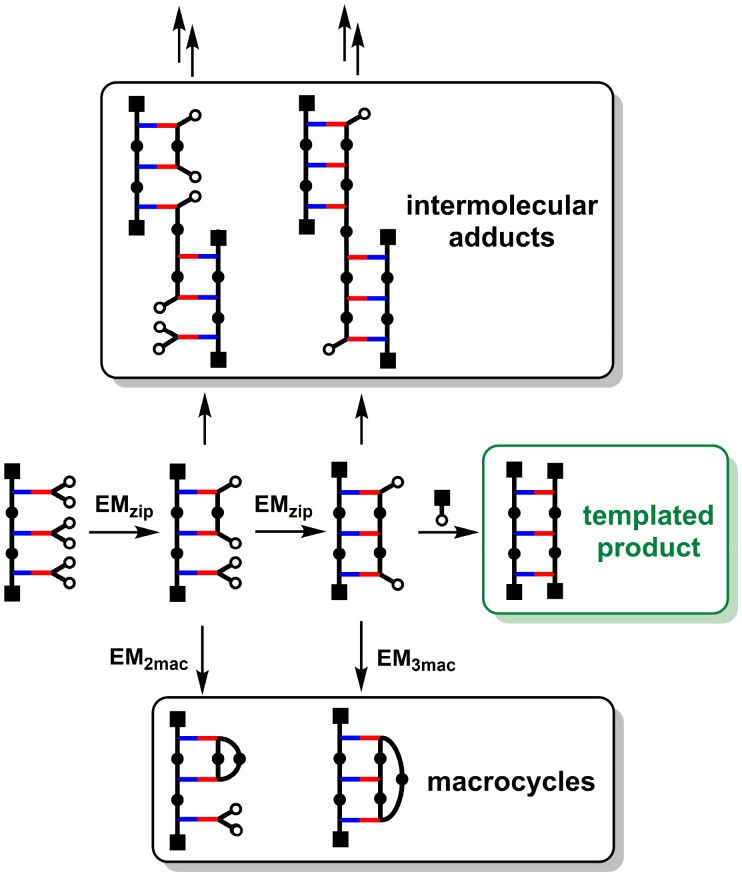
Competing pathways in the covalent template-directed synthesis of linear oligomers. Chain ends can react intramolecularly to form macrocycles and intermolecularly to form longer oligomers. The product distribution is determined by the concentration of capping agent, which competes with intermolecular processes, and the effective molarities for the intramolecular processes, EM_ZIP_, EM_2mac_ and EM_3mac_.

The crucial step that dictates the fidelity of the covalent template-directed replication process is the on-template oligomerization of the monomers in the ZIP step. Fig. 2 shows some of the competing reaction pathways that limit the yield of the duplex. The information encoded in the template is only transferred to the daughter strand if coupling reactions take place exclusively between adjacent monomers on the template (Fig. 2, middle channel). There are alternative intramolecular reactions that lead to macrocycles (Fig. 2, bottom channel) and intermolecular reactions that lead to longer oligomers (Fig. 2, top channel). A successful covalent template-directed replication experiment depends on minimisation of these undesired competing pathways.

*In situ* capping of the growing oligomer during the ZIP step proved to be an effective method for preventing the formation of intermolecular adducts.^22,23^ By operating at high dilution, where intramolecular templated reactions are much more favourable than intermolecular reactions, a large excess of the capping agent can be used to block the undesired intermolecular reactions shown in Fig. 2 without truncating the copy strand. This is possible because the kinetic effective molarity for the intramolecular ZIP reaction (EM_ZIP_) is two orders of magnitude higher than the concentration of cap required to block the intermolecular channel. However, the formation of macrocycles on the template is an intramolecular process and therefore concentration independent. In this paper, we discuss strategies to overcome undesired macrocyclization reactions in order to design viable candidates for the replication of chemical information using covalent template-directed synthesis.

**Fig. 3:**
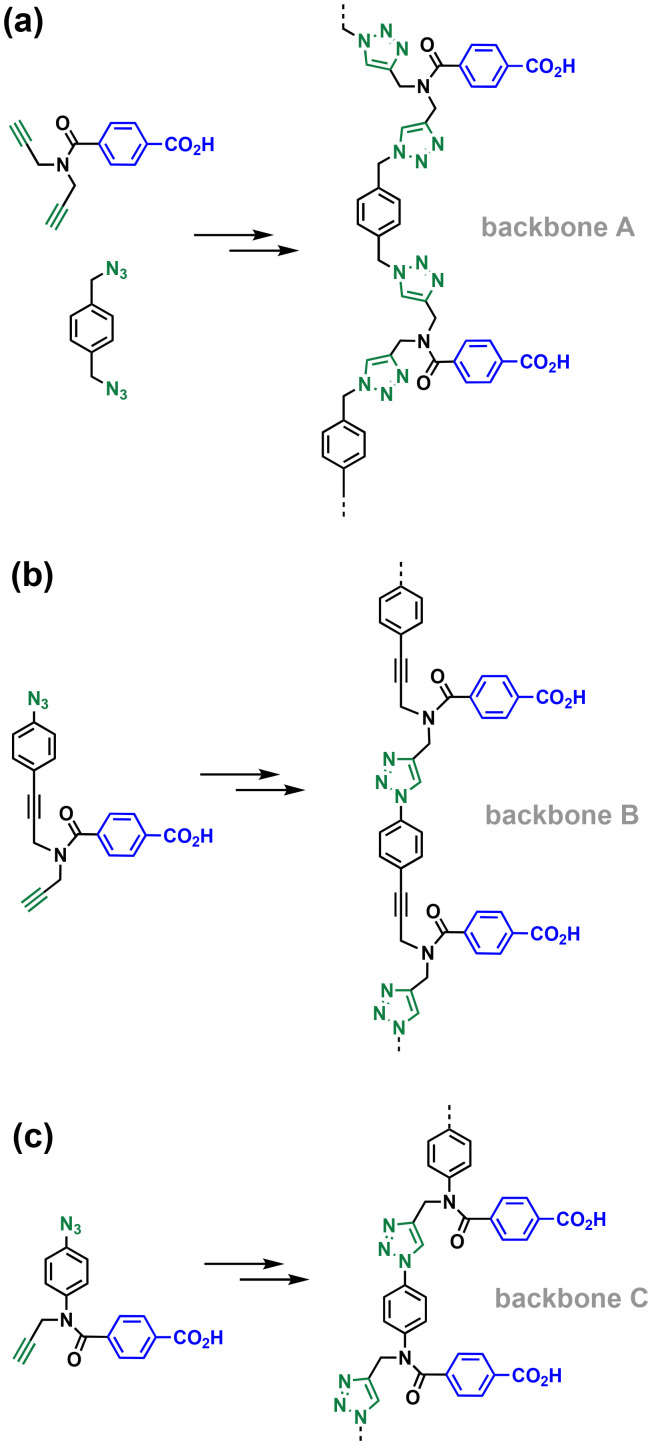
Three different backbones synthesised by CuAAC oligomerisation of the corresponding building blocks. (a) 1,4-Bis(triazolyl)methylbenzene (A). (b) *p*-Triazolylethynylbenzene (B). (c) *p*-Triazolylaniline (C).

The simplest macrocycle that can interfere with the formation of the copy stand during the ZIP step is composed of two monomer units. After two adjacent monomers react on the template to form a linear 2-mer, intramolecular reaction of the end groups can lead to the macrocyclic 2-mer. Similarly, after three adjacent monomers react on the template to form a linear 3-mer, intramolecular reaction of the end groups can lead to the macrocyclic 3-mer, and so on for longer oligomers. If the value of EM for the ZIP reaction (EM_zip_) is significantly greater than the values of EM for the formation of macrocycles (EM_2mac_, EM_3mac_*etc.*) then linear products predominate. These values of EM are all intrinsically connected to the conformational flexibility of the backbone and the geometries of the intermediates. Here, we investigate how the choice of monomer building block affects the conformational properties of the backbone and replication efficiency by directly comparing the three different architectures labelled A, B and C in Fig. 3. Backbone A is the most flexible system with four methylene groups in the chain connecting two base-pairing sites, backbone B has two methylene groups in the chain, and the most rigid backbone is C, which has only one methylene group between the base-pairing sites.

## Results and discussion

The first intramolecular reaction that can compete with duplex formation on the pathway shown in Fig. 2 is formation of the macrocyclic 2-mer, and we therefore focussed our attention on this reaction in the first instance. By examining the product distributions from templated oligomerisation reactions in the absence of a competing capping agent, it is possible to directly probe the relative rates of the macrocyclisation process shown in Fig. 2. This experiment requires synthesis of the monomer building blocks for the three different backbones, assembly of the 2-mer templates, and attachment of the monomers to the templates to give the corresponding pre-ZIP intermediates.

### Synthesis of pre-ZIP intermediates

**Scheme 1:**
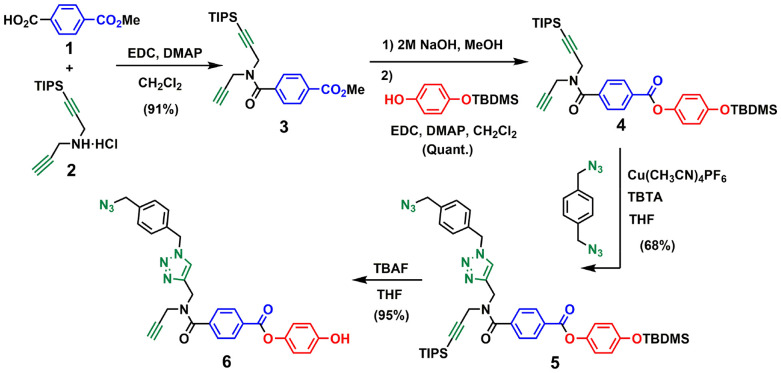
Synthesis of the phenol monomer building block of backbone A (6).

**Scheme 2:**
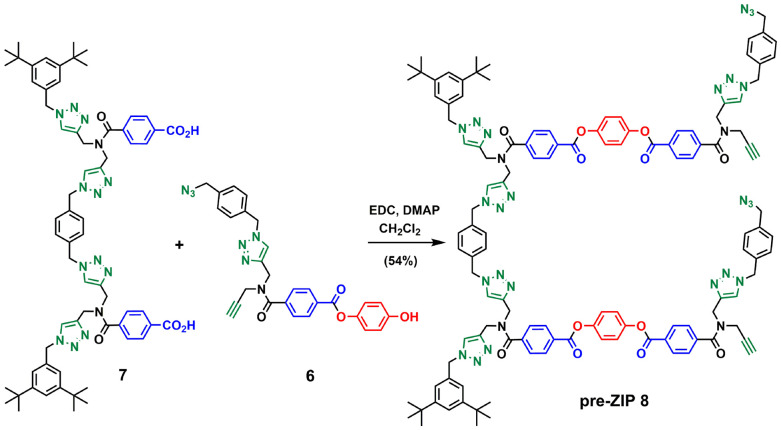
Synthesis of the pre-ZIP intermediate of backbone A (8).

For backbone A, the corresponding monomer equipped with an azide and an alkyne was prepared as shown in Scheme 1. The amide coupling of monomethyl terephthalic acid with mono-protected dipropargylamine 2 yielded 3 in excellent yield. Hydrolysis of 3 with NaOH and ester coupling of the resulting benzoic acid derivative with mono-protected hydroquinone^27^ gave 4 in excellent yield. CuAAC reaction of 4 with 1,4-bis(azidomethyl)benzene^27^ using copper(i) tetrakis(acetonitrile) hexafluorophosphate and tris[(1-benzyl-1*H*-1,2,3-triazol-4-yl)methyl]amine (CuTBTA)^28^ followed by TBAF deprotection of the silyl ether protecting groups provided monomer 6 in good yield. The 2-mer template 7 was prepared as described previously,^29^ and ester coupling with monomer 6 gave the pre-ZIP intermediate 8 (Scheme 2).

**Scheme 3:**
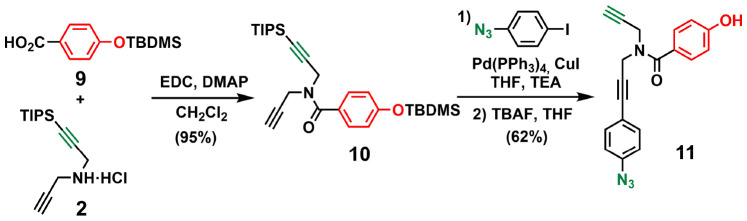
Synthesis of the phenol monomer building block of backbone B (11).

**Scheme 4:**
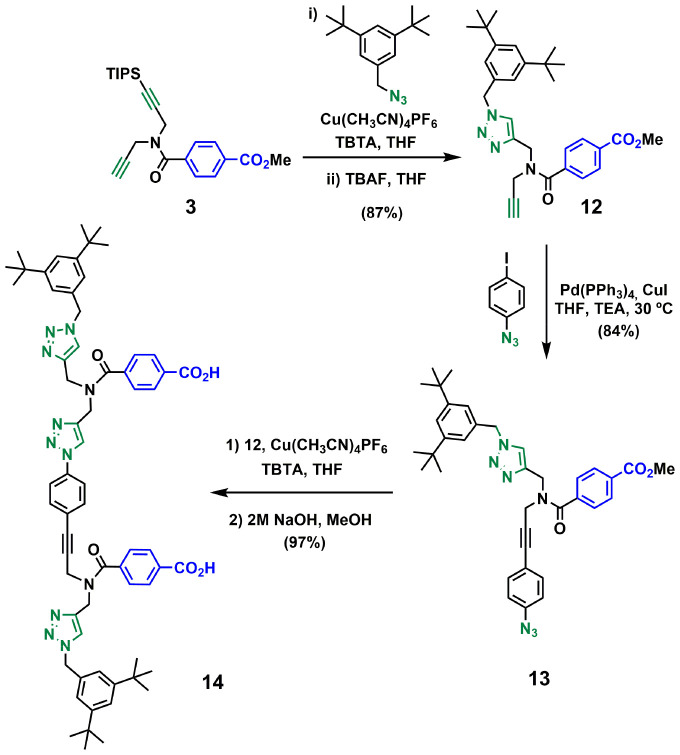
Synthesis of the 2-mer benzoic acid template of backbone B (14).

**Scheme 5:**
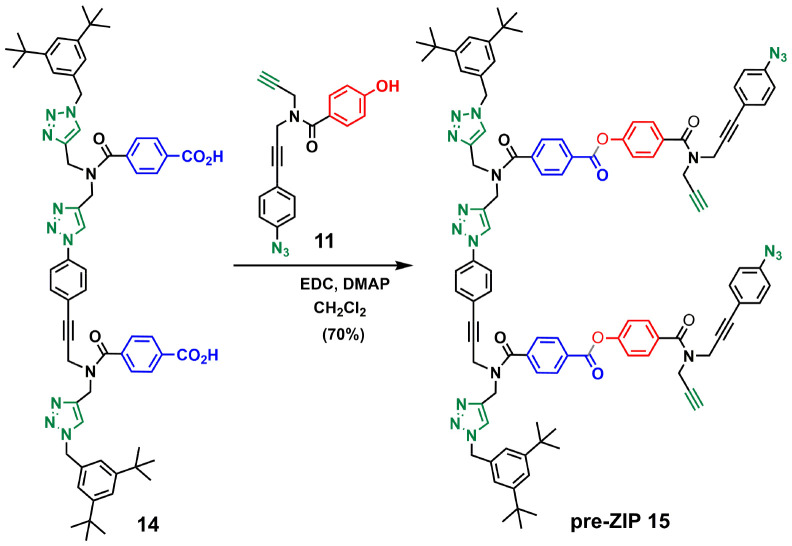
Synthesis of the pre-ZIP intermediate of backbone B (15).

Schemes 3–5 show the synthetic route for backbone B. First, amide coupling of protected *p*-hydroxybenzoic acid 9 and monoprotected propargylamine 2 gave access to 10 in excellent yield. Sonogashira coupling of 10 with 1-azido-4-iodobenzene^30^ followed by TBAF-mediated deprotection of silyl ether groups gave monomer 11 in good yield (Scheme 3). The 2-mer template of backbone B was made from compound 3. CuAAC capping reaction with 1-(azidomethyl)-3,5-di-*tert*-butylbenzene^27^ followed by *in situ* deprotection of the alkyne gave 12 (Scheme 4). Functionalization of the alkyne with a *p*-azidophenyl group was achieved in good yield by Sonogashira coupling with 1-azido-4-iodobenzene.^30^ CuAAC coupling between 12 and 13 followed by basic hydrolysis of the methyl esters gave the 2-mer template 14 in excellent yield. Pre-ZIP intermediate 15 was obtained in good yield by EDC-mediated ester coupling of template 14 with monomer 11 (Scheme 5).

**Scheme 6:**
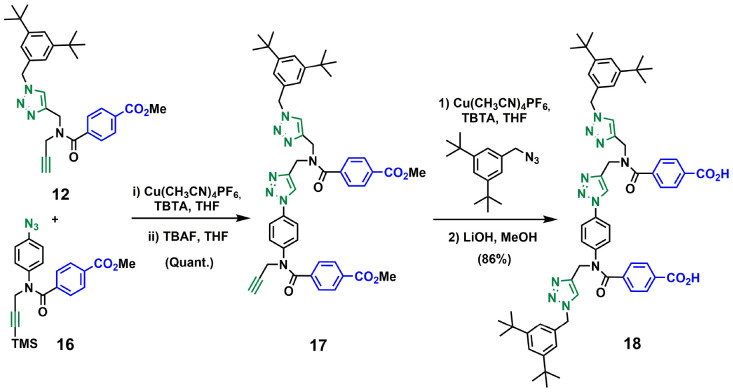
Synthesis of the 2-mer benzoic acid template of backbone C (18).

**Scheme 7:**
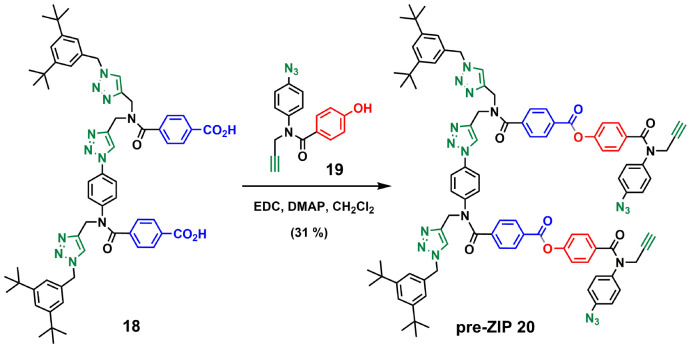
Synthesis of the pre-ZIP intermediate of backbone C (20).

Compound 12 also served as building block for the synthesis of the template for backbone C (Scheme 6). CuAAC coupling of 12^23^ with 16^21^ followed by removal of the TMS-protecting group gave 17 in excellent yield. Capping of 17 with 1-(azidomethyl)-3,5-di-*tert*-butylbenzene^27^ and subsequent basic hydrolysis gave the 2-mer template 18. Attachment of the phenol monomer 19 to the template 18 was achieved by ester coupling using EDC to give the pre-ZIP intermediate 20 (Scheme 7).

The base-pairing motif used with backbone A differs slightly from the base-pairs used with the other two backbones, because the building blocks were readily accessible. In pre-ZIP intermediates 15 and 20, the phenol base is directly attached to the backbone *via* an amide bond, but in pre-ZIP intermediate 8, there is an additional aryl ester spacer, so this base-pair is somewhat longer than in the other two systems. We have previously reported experiments on a number of different base-pairing motifs using backbone C and have not found any significant differences in the values of EM associated with such differences in the length of the base-pair.^14,15^ Nevertheless it is possible that this difference in geometry makes an additional contribution to any differences observed in the behaviour of backbone A.

### ZIP experiments

**Fig. 4:**
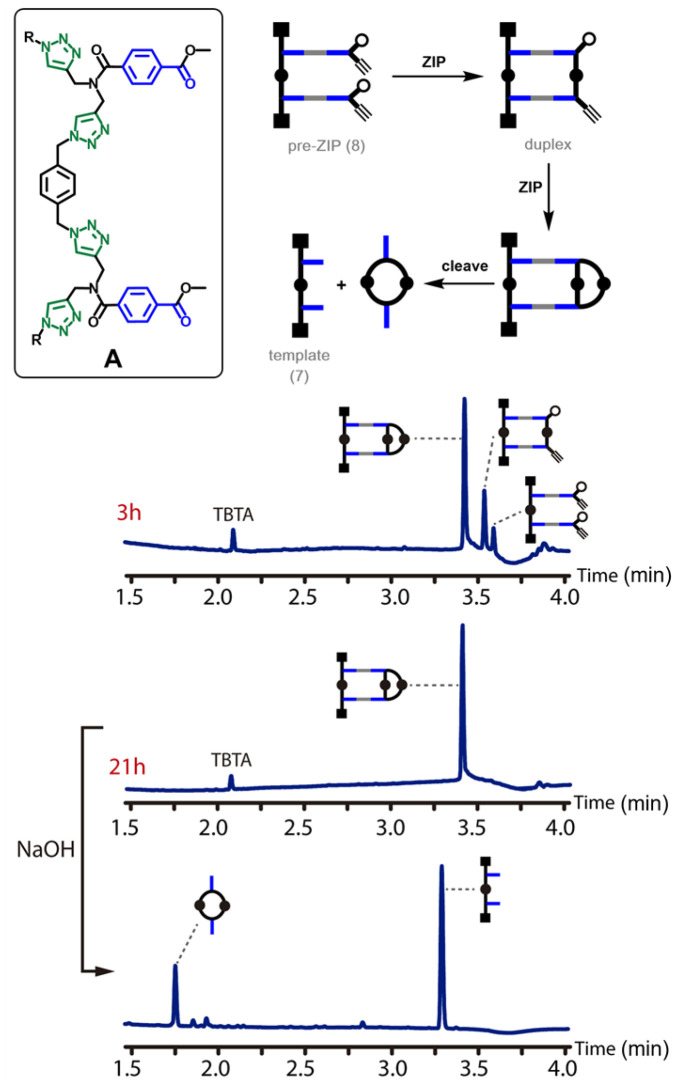
ZIP reaction for backbone A. Schematic representation and UPLC traces of the CuAAC reaction of pre-ZIP 8 (250 μM in THF) after 3 h and 21 h, and after subsequent hydrolysis. Conditions: C18 column at 40 °C (254 nm) using water + 0.1% formic acid (A) and CH_3_CN + 0.1% formic acid (B); gradient of 0–3 minutes 5–95% B + 2 minutes 95% B.

The pre-ZIP intermediates 8, 15 and 20 were treated with copper(i) salts and TBTA under dilute conditions (50–250 μM). Fig. 4 shows the outcome of the CuAAC reaction for backbone A. After 3 hours of reaction, the main species in the reaction mixture was the macrocyclic product, and after 21 hours, the starting material had been quantitatively converted into this product. Cleavage of the ester bonds by basic hydrolysis regenerated the template 7 along with the macrocyclic 2-mer.

**Fig. 5:**
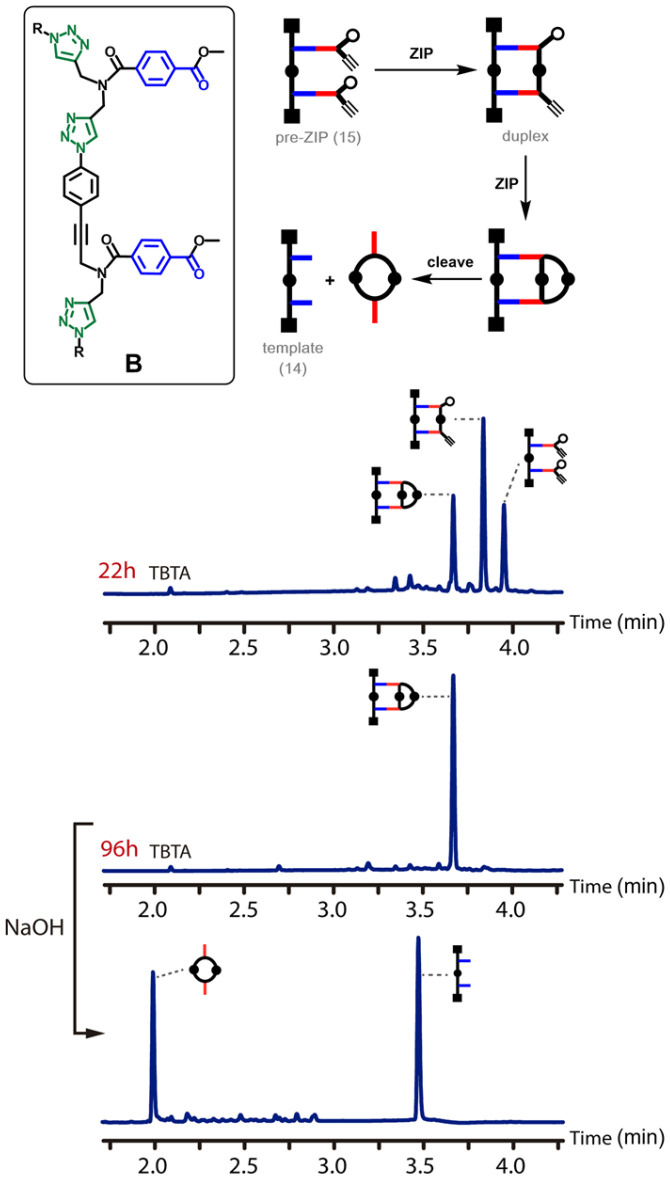
ZIP reaction for backbone B. Schematic representation and UPLC traces of the CuAAC reaction of pre-ZIP 15 (50 μM in THF) after 22 h and 96 h, and after subsequent hydrolysis. Conditions: C18 column at 40 °C (254 nm) using water + 0.1% formic acid (A) and CH_3_CN + 0.1% formic acid (B); gradient of 0–3 minutes 5–95% B + 2 minutes 95% B.

Fig. 5 shows the outcome of the CuAAC reaction for backbone B. In this case, the reaction is much slower. In contrast to backbone A, there was still a substantial amount of starting material present in the reaction mixture after 22 hours. Although some of the macrocyclic product was observed, the major species in the reaction mixture was the duplex. It is clear that the rates of both the ZIP reaction and the macrocyclisation reaction are much slower for backbone B than for backbone A. After 96 hours, the starting material had been quantitatively converted into the macrocyclic product, and cleavage of the ester bonds by basic hydrolysis regenerated the template 14 along with the macrocyclic 2-mer as the only products.

**Fig. 6:**
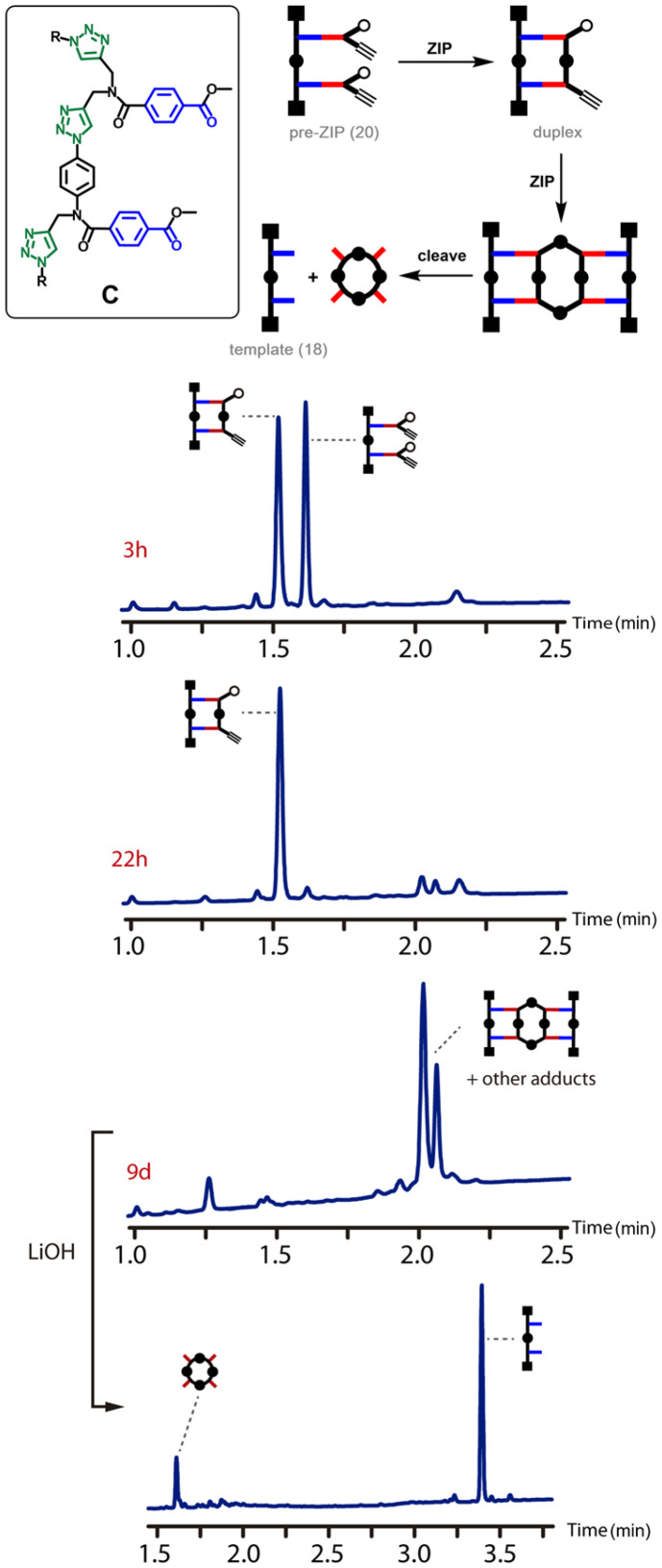
ZIP reaction for backbone C. Schematic representation and UPLC traces for the CuAAC reaction of pre-ZIP 20 (50 μM in THF) after 3 h, 22 h and 9 days, and after subsequent hydrolysis. Conditions: C18 column at 40 °C (254 nm) using water + 0.1% formic acid (A) and CH_3_CN + 0.1% formic acid (B); gradient of 0–2 minutes 65–100% B + 1 minute 100% B for the CuAAC reaction (top three chromatograms), and gradient of 0–4 minutes 5–100% B + 1 minute 100% B for the hydrolysis (bottom chromatogram).

Fig. 6 shows the outcome of the CuAAC reaction for backbone C. The starting material was quantitatively converted in the duplex after 22 hours, and there was no sign of the macrocyclic species observed for the other two backbones. In this case, the ZIP reaction is fast, and the macrocyclization reaction is slow. The reaction mixture was monitored over 9 days, and none of the macrocyclic product was observed. However, the products of intermolecular reactions between two duplexes were observed. Basic hydrolysis of the reaction mixture regenerated the template 18 along with the macrocyclic 4-mer as the only products (LiOH was used to minimise hydrolysis of the amides). The two peaks observed in the UPLC trace recorded after 9 days and before hydrolysis presumably correspond to different isomers of the adduct shown, which results from the directionality of backbone C.

### Computational analysis

The experimental results shown in Fig. 4–6 provide a useful benchmark to test whether computational methods can be used to design monomers suitable for template-directed replication. The ZIP reaction appears to be somewhat slower for backbone B and somewhat faster for backbone A, but there are much larger differences in the rates of macrocyclisation. The propensity for macrocyclization increases in the following order:backbone C ≪ backbone B < backbone A.

**Fig. 7:**
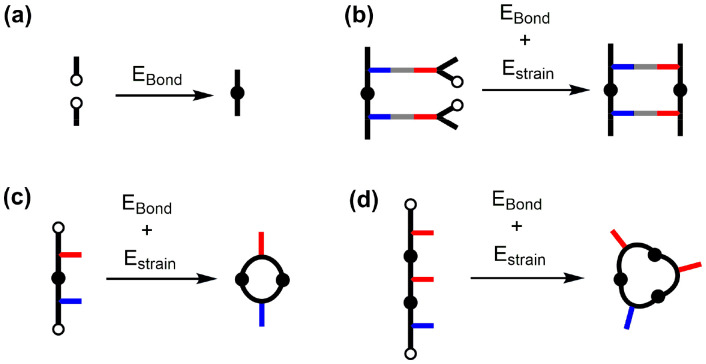
Models used to calculate *E*_bond_ (a), and the ring strain energies for the ZIP reaction (b), 2-mer macrocyclization (c) and 3-mer macrocyclization (d).

For each backbone, the ring strain (*E*_strain_) associated with both the ZIP and macrocyclisation reactions was calculated using molecular mechanics. The energy associated with formation of the triazole linkage (*E*_Bond_) is obtained using the difference in energy between the two fragments and the final oligomer backbone (eqn (1) and Fig. 7a).

1 *E*_Bond_ = *E*_backbone_ − *E*_frag1_ − *E*_frag2_

The ring strain is obtained from the difference in energy between the final structure (*E*_closed_) and the precursor (*E*_open_), allowing for the energy associated with the triazole linkage (eqn (2)).

2 *E*_strain_ = *E*_closed_ − *E*_open_ − *E*_bond_

Fig. 7b and c illustrate the structures used to calculate *E*_open_ and *E*_closed_ in eqn (2) to obtain the ring strain associated with the ZIP reaction and with formation of the macrocyclic 2-mer. Similar calculations can be carried out to assess the ring strain of larger macrocycles, such as the macrocyclic 3-mer illustrated in Fig. 7d. Strictly, the calculation of ring strain for the macrocycles should be carried out for formation of the macrocycle attached to the template. Attachment to the template constrains the conformation of the backbone and increases the ring strain associated with macrocycle formation (see ESI† for details of these calculations). However, the difficulty in reliably sampling conformational space increases dramatically with the size of the molecule, so we prefer to focus on the simple macrocycles to obtain a ring strain that represents a lower limit on the ring strain associated with macrocycle formation in the templated reaction.

**Fig. 8:**
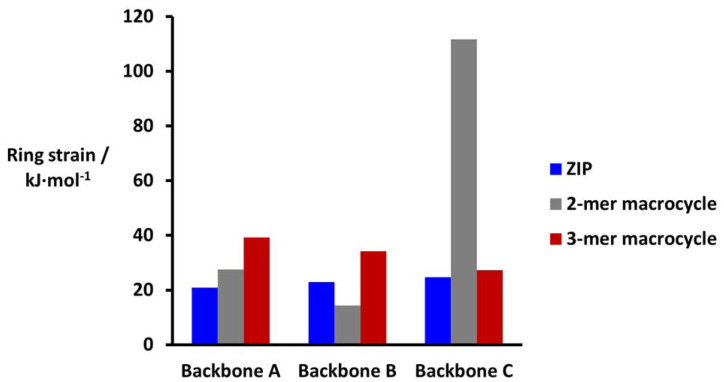
Ring strain energies (kJ mol^−1^) for the ZIP and macrocyclisation reactions calculated using eqn (1) and (2) and the energies of the lowest energy conformations from molecular mechanics conformational searches with the MMFFs force-field and CHCl_3_ solvation.^32,33^

The values of the ring strain energies are plotted in Fig. 8 for the three different backbones. For the ZIP reaction, there is almost no ring strain for any of the backbones: the values are 20–25 kJ mol^−1^, which is comparable to the ring strain for cyclopentane (26 kJ mol^−1^).^31^ Similar ring strain energies were obtained for formation of the 2-mer macrocycles with backbones A and B. However for backbone C, the calculated ring strain for formation of the 2-mer macrocycle is over 100 kJ mol^−1^. These calculations are in good agreement with the experimental results. The only reaction for which there is substantial ring strain is the macrocyclisation process on backbone C, and this is the only intramolecular reaction that was not observed experimentally. These results suggest that ring strain calculations using molecular mechanics should provide a useful tool for the rational design of backbones with the right properties for covalent template-directed replication.

The calculated ring strain energies also indicate that there is almost no strain associated with formation of the macrocyclic 3-mers for all three backbones (Fig. 8). We have previously investigated the templated formation of the macrocyclic 3-mer with backbone C. As predicted by the ring strain calculations, oligomerization on a 3-mer template led to quantitative formation of the macrocyclic 3-mer. Experiments using a 3-mer template of backbone B gave a mixture of the macrocyclic 2-mer and the macrocyclic 3-mer as the only major products (see ESI† for details). Thus, different strategies are required to avoid the formation of larger macrocycles, which are difficult to prevent by using ring strain in the backbone design.^22,23^

## Conclusions

The experiments described here show that oligomerisation reactions on 2-mer templates provide a useful tool for assessing the viability of different molecular designs for synthetic replication systems. Three closely related oligotriazole backbones were investigated, and the results show that backbone flexibility is an important parameter that governs the relative probability of obtaining macrocyclic or linear products in template-directed oligomerisation reactions. For very flexible backbones, macrocyclisation reactions on the template take place at similar rates to the intramolecular reactions that lead to the formation of linear oligomers. However, no macrocyclic products were formed on the template when a more rigid monomer building block was used.

In conventional polymerisation processes, it is possible to bias the product distribution in favour of linear oligomers by operating at high concentrations, which accelerates intermolecular reactions relative to intramolecular cyclisations. A similar strategy is not possible in the corresponding templated process, because both types of reaction are intramolecular and independent of the operating concentration. The results presented here imply that the attachment of monomers to a linear template does not significantly bias the product distribution against macrocycles, so template-directed synthesis of linear oligomers requires relatively rigid monomer building blocks that do not form macrocycles in the absence of template. However, care must be taken in the design of monomer building blocks to ensure that the rigidity required to avoid macrocycles does not also adversely affect the ZIP reaction that leads to duplex formation.

We show that ring strain calculations using molecular mechanics provides a straightforward method for assessing whether different backbone designs can reduce the probability of macrocycle formation without impacting the ZIP reaction. In the systems studied here, products with ring strain energies of the order 20–30 kJ mol^−1^ were formed rapidly, but when the ring strain was over 100 kJ mol^−1^, none to that product was observed. These energies were calculated using molecular mechanics and the MMFFs force-field, but the energy differences required to discriminate between low strain and high strain products are so high that the results are unlikely to be sensitive to the force-field or the level of theory used.

The identification of structural requirements for the rational design of suitable backbones completes the list of criteria which must be met for efficient covalent template-directed replication of synthetic oligomers. The different aspects to consider are:

(1) Covalent base-pairing. A high-yielding, orthogonal and reversible reaction is required for the development of covalent base-pairing. Interestingly, these are the same as the requirements for efficient protecting group chemistry.^34^ The covalent base-pair must be formed from two distinct reactive sites, so the information can be encoded as a sequence of these groups. An orthogonal synthetic strategy is required for the selective loading of complementary monomers onto a mixed sequence template. Ester chemistry satisfies all these requirements. Formation and cleavage are clean high-yielding reactions and orthogonality can be achieved by selective protection of alcohols in the presence of carboxylic acids.^21^

(2) ZIP reaction. The oligomerization reaction must also be high yielding, robust and compatible with a wide range of chemical functionalities.^35^ In addition, the reaction must be compatible with high dilution conditions, which are required to prevent the formation of intermolecular adducts. Click reactions are well-suited to efficient low dilution oligomerization processes,^36,37^ and CuAAC is the paramount example,^38–40^ which we have exploited for covalent templating.^26^

(3) Backbone flexibility. As described here, the flexibility of the backbone is a critical design element. The monomers must be rigid enough to prevent the formation of macrocycles, but not so rigid that the ZIP reaction is inhibited. Molecular mechanics can be used as a tool for assessing the viability of potential backbones by calculation of the ring strain associated with the different intramolecular reaction pathways that can occur on the template.

(4) Directionality of the backbone. The best performing backbone C is directional, so the daughter strand can have a parallel or antiparallel arrangement relative to the template. Either arrangement would result in a complementary copy of the template, but a mixture of both directions on a single template would lead to truncated copy strands. For backbone C, we have demonstrated that the antiparallel ZIP is preferred over the parallel one, which minimises this problem.^22^ Alternatively, the use of symmetrical monomers would prevent any directionality issues. For example, template-directed synthesis of backbone A from dialkyne and diazide building blocks would result in a single duplex rather than the parallel–antiparallel mixture obtained for backbone C.

(5) *In situ* capping. End-capping strategies can be used to avoid undesired macrocyclization and intermolecular reactions during the ZIP step.^22,23,26^ The concentration of capping agent must be ideally 2 orders of magnitude lower than EM_ZIP_ for 99% efficacy, and the concentration of pre-ZIP intermediate should be another 2 orders of magnitude lower. Capping agents will also intercept macrocycle formation if EM_mac_ is orders of magnitude lower than EM_ZIP_.

## Conflicts of interest

There are no conflicts to declare.

## Supplementary Material

OB-020-D2OB01627C-s001
