# Supplementary material for: Effect of backbone flexibility on covalent template-directed synthesis of linear oligomers
Source: Org Biomol Chem. 2022 Oct 13;20(42):8285–92. doi: 10.1039/d2ob01627c (PMC9629452; doi:10.1039/d2ob01627c)
Supplement: OB-020-D2OB01627C-s001 [file OB-020-D2OB01627C-s001.pdf]

Electronic Supplementary Material (ESI) for Organic & Biomolecular Chemistry.
This journal is © The Royal Society of Chemistry 2022

Supporting information

Effect of backbone flexibility on covalent template-directed synthesis of linear oligomers

Diego Núñez-Villanueva and Christopher A. Hunter*

Department of Chemistry, University of Cambridge, Lensfield Road, Cambridge CB2 1EW, UK. E-mail: herchelsmith.orgchem@ch.cam.ac.uk

TABLE OF CONTENTS **Page**

General experimental details. S2

Synthesis and characterization of described compounds. S3

CuAAC reaction of pre-ZIP 8. S74

CuAAC reaction of pre-ZIP 15. S78

CuAAC reaction of pre-ZIP 20. S82

CuAAC reaction of pre-ZIP S7 S87

Molecular modelling calculations. S89

References. S98

General experimental details.

All the reagents and materials used in the synthesis of the compounds described below were bought from commercial sources, without prior purification. Dry THF and CH_2Cl_2 were obtained from a solvent purification system (Pure Solv™, Innovative Technology, Inc.). Anhydrous DMF was purchased from Sigma-Aldrich. Thin layer chromatography was carried out using with silica gel 60F (Merck) on glass plates. Flash chromatography was carried out on an automated system (Combiflash Rf+ or Combiflash Rf Lumen) using prepacked cartridges of silica (25µ PuriFlash® columns). All NMR spectroscopy was carried out on a Bruker 400 MHz DPX400, 400 MHz AVIII400, 500 MHz DCH cryoprobe or 500 MHz TCI Cryoprobe spectrometer using the residual solvent as the internal standard. All chemical shifts (δ) are quoted in ppm and coupling constants given in Hz. Splitting patterns are given as follows: s (singlet), bs (broad singlet), d (doublet), t (triplet), q (quadruplet), m (multiplet). FT-IR spectra were measured on a PerkinElmer Spectrum One spectrometer equipped with an ATR cell. Melting points were measured in a Mettler Toledo MP50 Melting Point System. UPLC analysis of samples was performed using Waters Acquity H-class UPLC coupled with a single quadrupole Waters SQD2. ACQUITY UPLC CSH C18 Column, 130Å, 1.7 µm, 2.1 mm X 50 mm was used as the UPLC column. The conditions of the UPLC method are as follows: gradients of water +0.1% formic acid (solvent A) and acetonitrile +0.1% formic acid (solvent B=) as specified in each case. Flow rate: 0.6 ml/min; Column temperature of 40°C; Injection volume of 2 µL. The signal was monitored at 254 nm. HRMS analysis was performed in a Waters LCT Premier equipped with a TOF mass analyser and W optics for enhanced resolution, using 50% aqueous acetonitrile with 0.25% formic acid as mobile phase.

Compounds **2**[S1], **3**[S1], **7**[S3], **12**[S1], **16**[S2], **17**[S1] and **19**[S2] have been previously described.

Synthesis and characterization of described compounds

Compound 4.

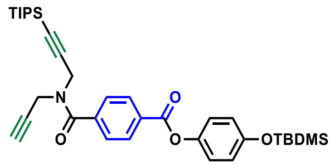

To a solution of **3** (0.346 g, 0.84 mmol) in MeOH (5 mL) was added 2 M NaOH solution (1.09 mL, 2.19 mmol). The reaction was stirred overnight at room temperature and then the solution was carefully quenched with 5% dilute HCl and extracted with EtOAc (3x) followed by washing with H_2O and brine. The organic layer was dried over anhydrous $MgSO_4$ and concentrate under vacuum, affording the corresponding carboxylic acids (0.325 g, 97 %) as a light yellow solid. To this material, 4-((*tert*-butyldimethylsilyl)oxy)phenol[S3] (0.183 g, 0.82 mmol), EDC (0.172 g, 0.90 mmol) and DMAP (0.010 g, 0.08 mmol) were added and dissolved under N_2 atmosphere in dry CH_2Cl_2 (10 ml). The solution was stirred under N_2 atmosphere at room temperature for 1 h. The crude was diluted with EtOAc (20 mL) and washed with 5% aq. soln. HCl (3x), H_2O (1x) and brine. The organic phase was dried with $MgSO_4$ and concentrated *in vacuo*. Compound **4** was obtained as a yellow oil (0.493 g, quantitative; overall yield: 97%) as a clear oil.

^{1}H NMR (400 MHz, CDCl$_3$): δ_H = 8.24 (d,2H, *J* = 8.0 Hz), 7.70 (bs, 2H), 7.07 (d, 2H, *J* = 9.0 Hz), 6.87 (d, 2H, *J* = 9.0 Hz), 4.51 and 4.17 (bs, 4H, splitting is due to the existence of rotamers around the amide bond; only a singlet would be expected in the absence of rotamers), 2.31 (s, 1H), 1.09 (m, 21H), 1.00 (s, 9H), 0.22 (s, 6H).

^{13}C NMR (100.6 MHz, CDCl$_3$): δ_C = 172.3, 170.9, 153.7, 152.7, 144.8, 137.3, 130.5, 130.4, 127.4, 122.4, 120.8, 96.9, 85.1, 77.7, 72.7, 38.5, 33.5, 25.8, 19.5, 18.7, 11.3, -4.3.

HRMS (ES+): calcd for $C_{35}H_{50}NO_4Si_2$ 604.3278 $[M+H]^+$, found 604.3280 $[M+H]^+$.

FT-IR (ATR): ν_{max} 2930, 2864, 1740, 1653, 1502, 1462, 1258, 1191, 1075, 915 and 775 cm^{-1}.

^{1}H-NMR (400 MHz, $CDCl_3$) Compound 4.

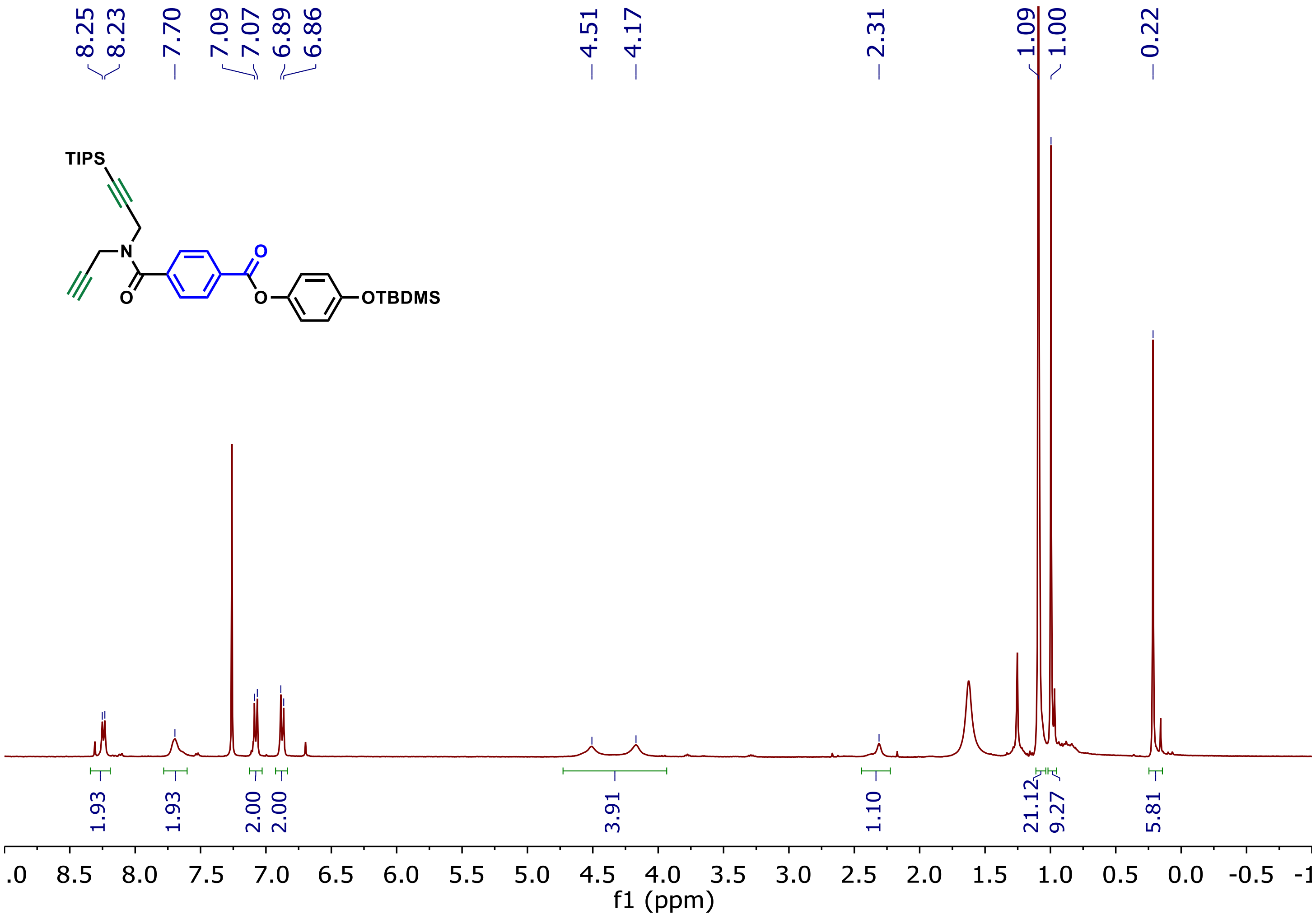

^{13}C-NMR (100.6 MHz, CDCl$_3$) Compound 4.

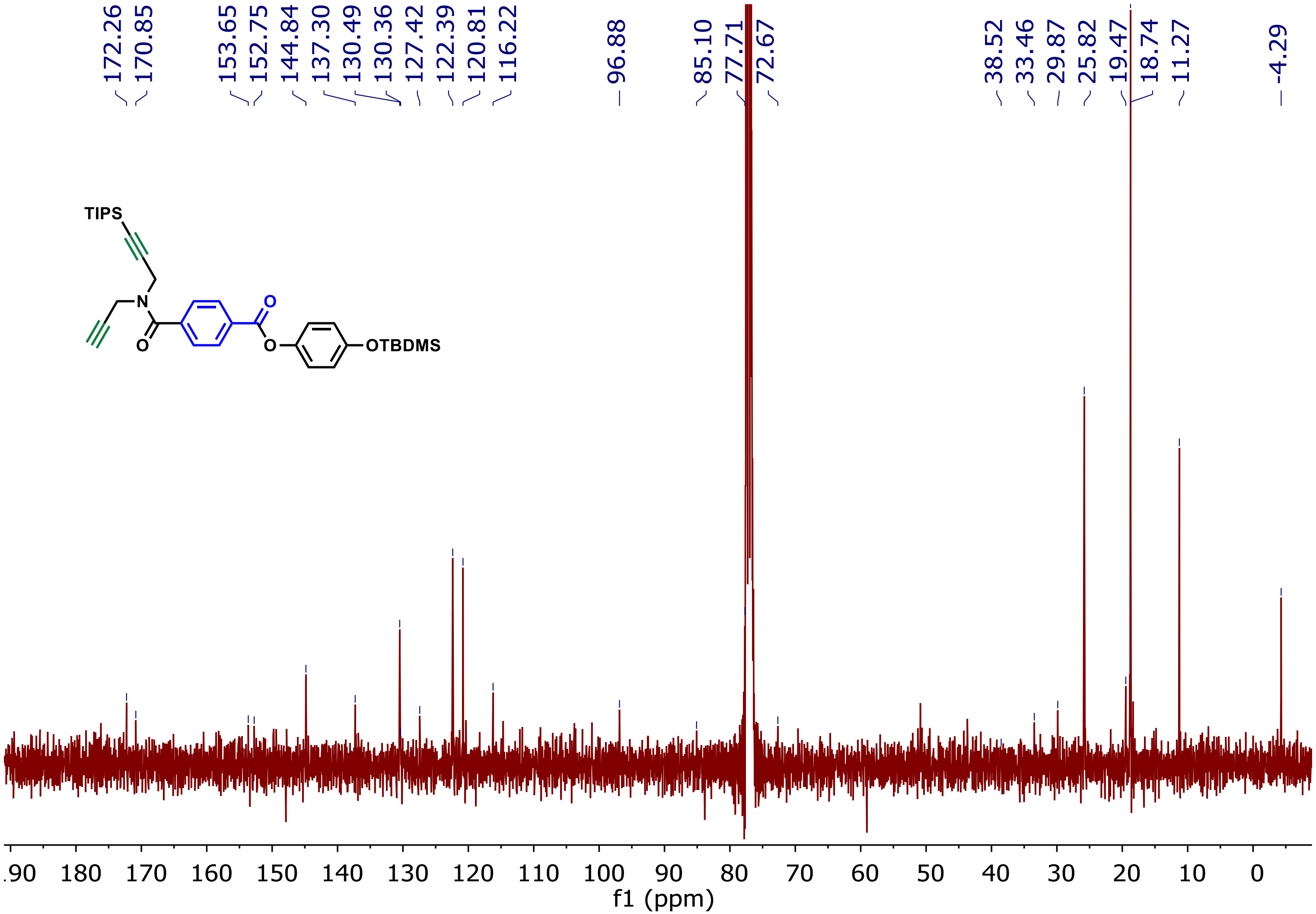

Compound 5.

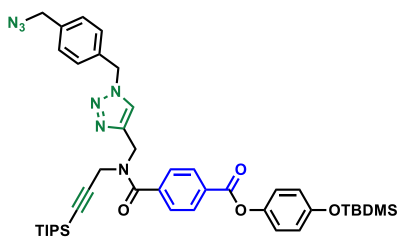

1,4-bis(azidomethyl)benzene[S4] (0.1.578 g, 8.39 mmol), $Cu(CH_3CN)_4PF_6$ (0.044 g, 0.08 mmol) and TBTA (0.044 g, 0.08 mmol) were mixed in a round-bottom flask and, under N_2, THF (120 mL) was added. A solution of **4** (0.506 g, 0.84 mmol) in THF (2 mL) was added and the reaction was stirred overnight at room temperature. The solvent was evaporated to dryness and the crude was purified by flash column chromatography on silica gel (gradient from 10% to 100% of EtOAc in Pet. Ether) to afford compound **5** (0.452 g, 68%) as a foam, and the corresponding disubstituted compound (0.050 g, 8%).

^{1}H NMR (400 MHz, $CDCl_3$): δ_H = 8.20 (d, 2H, J = 8.5 Hz), 7.69 (d, 2H, J = 8.5 Hz), 7.66 (s partially overlapped, 1H) ,7.33 (m, 4H), 7.07 (d, 2H, J = 9.0 Hz), 6.87 (d, 2H, J = 9.0 Hz), 5.53 (s, 2H), 4.85 and 4.11 (bs, 4H), 4.36 (s, 2H), 1.10 (m, 21H), 0.99 (s, 9H), 0.21 (s, 6H).

^{13}C NMR (125.7 MHz, $CDCl_3$): δ_C = 170.2, 164.8, 153.6, 144.8, 139.8, 136.4, 134.7, 131.4, 130.7, 130.6, 130.4, 129.1, 128.8, 127.5, 123.6, 122.4, 120.8, 101.9, 77.6, 54.4, 54.0, 40.6 and 40.3, 25.8, 18.8, 18.4, 11.3, -4.3.

HRMS (ES+): calcd for $C_{43}H_{58}N_7O_4Si_2$ 792.4089 $[M+H]^+$, found 792.4084 $[M+H]^+$.

FT-IR (ATR): ν_{max} 2923, 2852, 2099, 1738, 1642, 1502, 1259, 1192 and 773 cm^{-1}.

^{1}H-NMR (400 MHz, $CDCl_3$) Compound 5

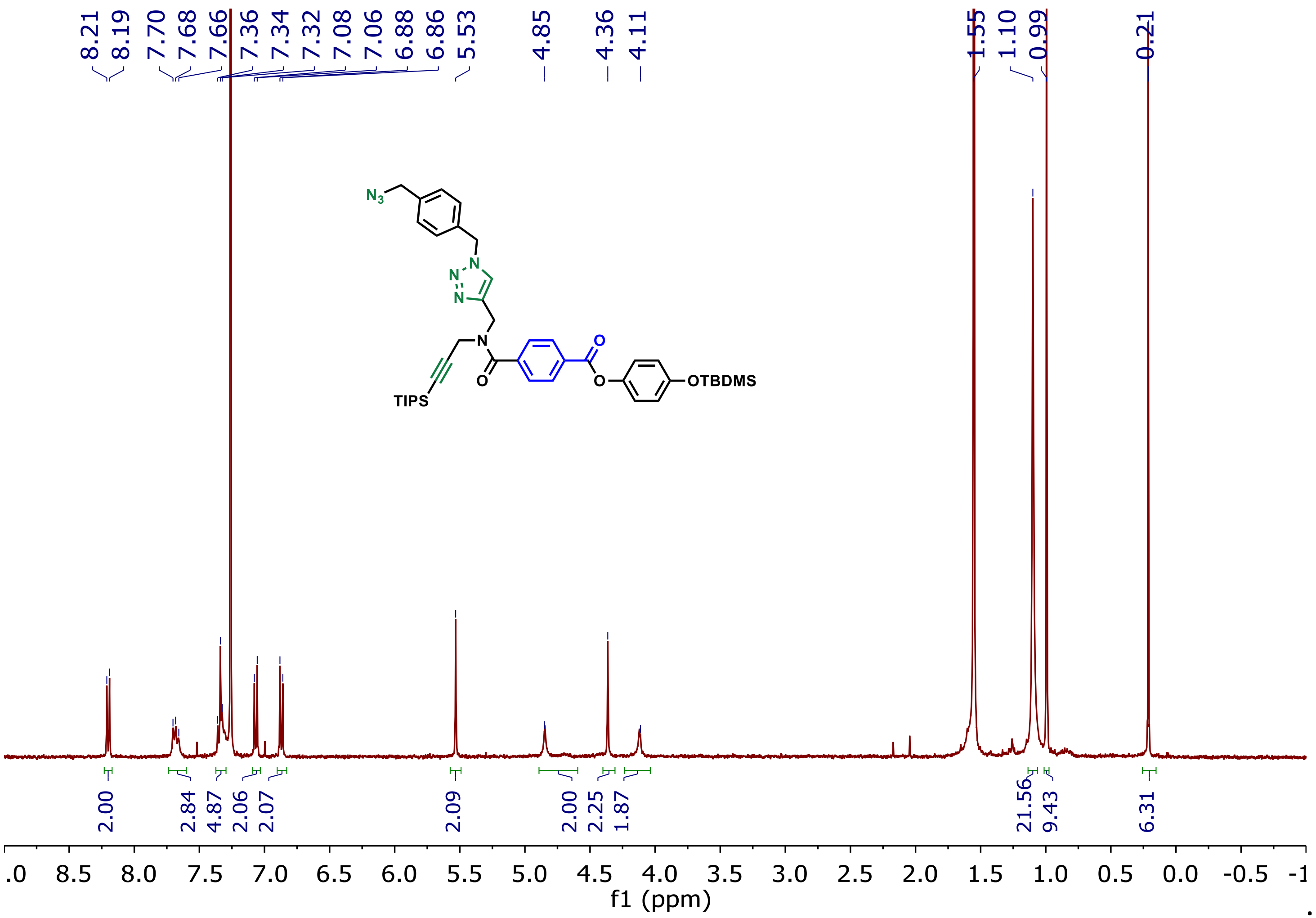

^{13}C-NMR (125.7 MHz, $CDCl_3$) Compound 5.

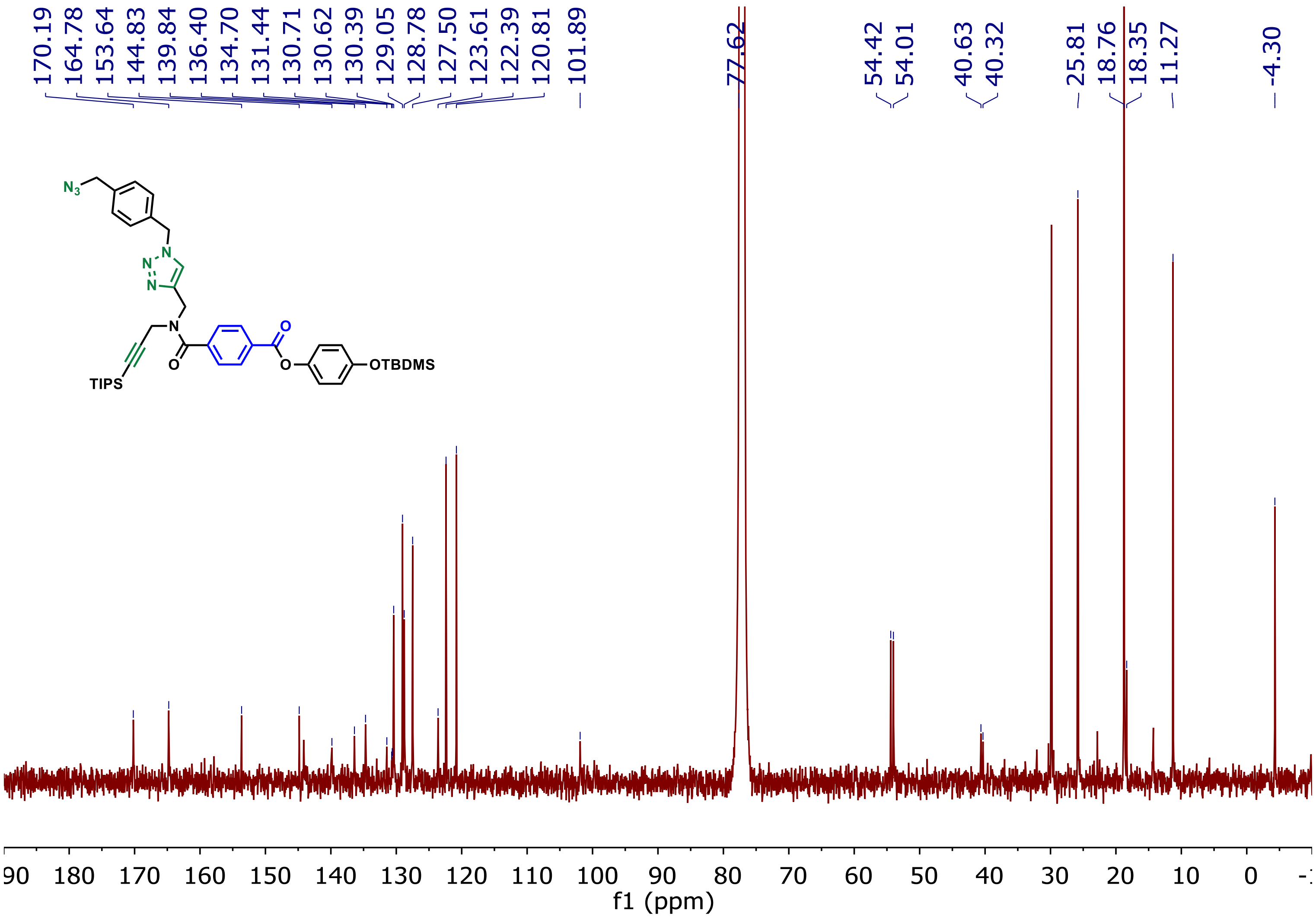

Compound 6.

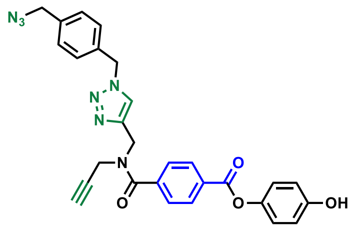

A solution of **5** (0.452 g, 0.57 mmol) in dry THF (4 mL) was treated at 0 °C with TBAF solution (1M in THF, 1.14 mL, 1.14 mmol). The reaction was stirred for 5 minutes and quenched with 5% soln. HCl and extracted with EtOAc (3x) followed by washing with H_2O and brine The organic layer was dried over anhydrous $MgSO_4$ and concentrate under vacuum. The crude material was purified by flash column chromatography on silica gel (gradient from 10% to 100%of EtOAc in Pet. Ether) to afford compound **6** (0.284 g, 95%) as a light yellow solid.

Melting point: 154-156 °C.

^{1}H NMR (400 MHz, CDCl$_3$): δ_H = 8.22 (d, 2H, *J* = 8.0 Hz), 7.66 (s partially overlapped, 1H), 7.64 (m, 2H), 7.34 (m, 4H), 7.07 (d, 2H, *J* = 9.0 Hz), 6.86 (d, 2H, *J* = 9.0 Hz), 5.54 (s, 2H), 5.20, 4.86, 4.67 and 4.10 (bs, 4H, splitting is due to the existence of rotamers around the amide bond; only two singlets would be expected in the absence of rotamers), 4.36 (s, 2H), 2.36 and 2.29 (bs, 1H).

^{13}C NMR (100.6 MHz, CDCl$_3$): δ_C = 170.4, 164.9, 154.0, 144.2, 143.9, 139.6, 132.5, 130.5, 129.1, 128.8, 127.6, 127.5, 126.0, 123.7, 122.6, 116.3, 78.3, 73.7, 54.4, 54.0. N-CH$_2$ peaks are not listed due to broadness caused by the existence of rotamers.

HRMS (ES+): calcd for $C_{28}H_{24}N_7O_4$ 522.1890 [M+H]$^+$, found 522.1891 [M+H]$^+$.

FT-IR (ATR): ν_{max} 2922, 2851, 2098, 1732, 1635, 1506, 1264, 1193, 1077 and 774 cm^{-1}.

^{1}H-NMR (400 MHz, $CDCl_3$) Compound 6.

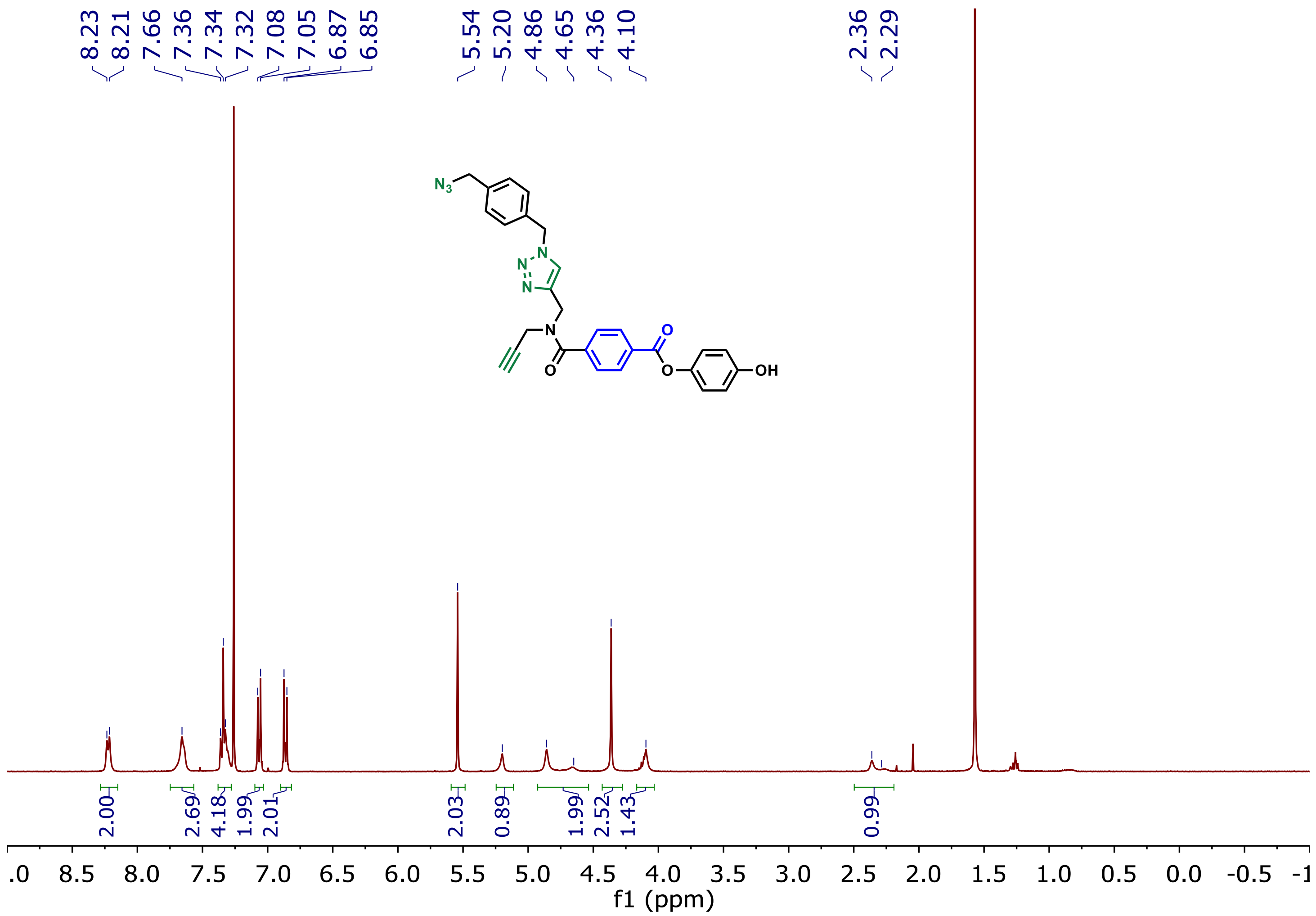

^{13}C-NMR (100.6 MHz, CDCl$_3$) Compound 6.

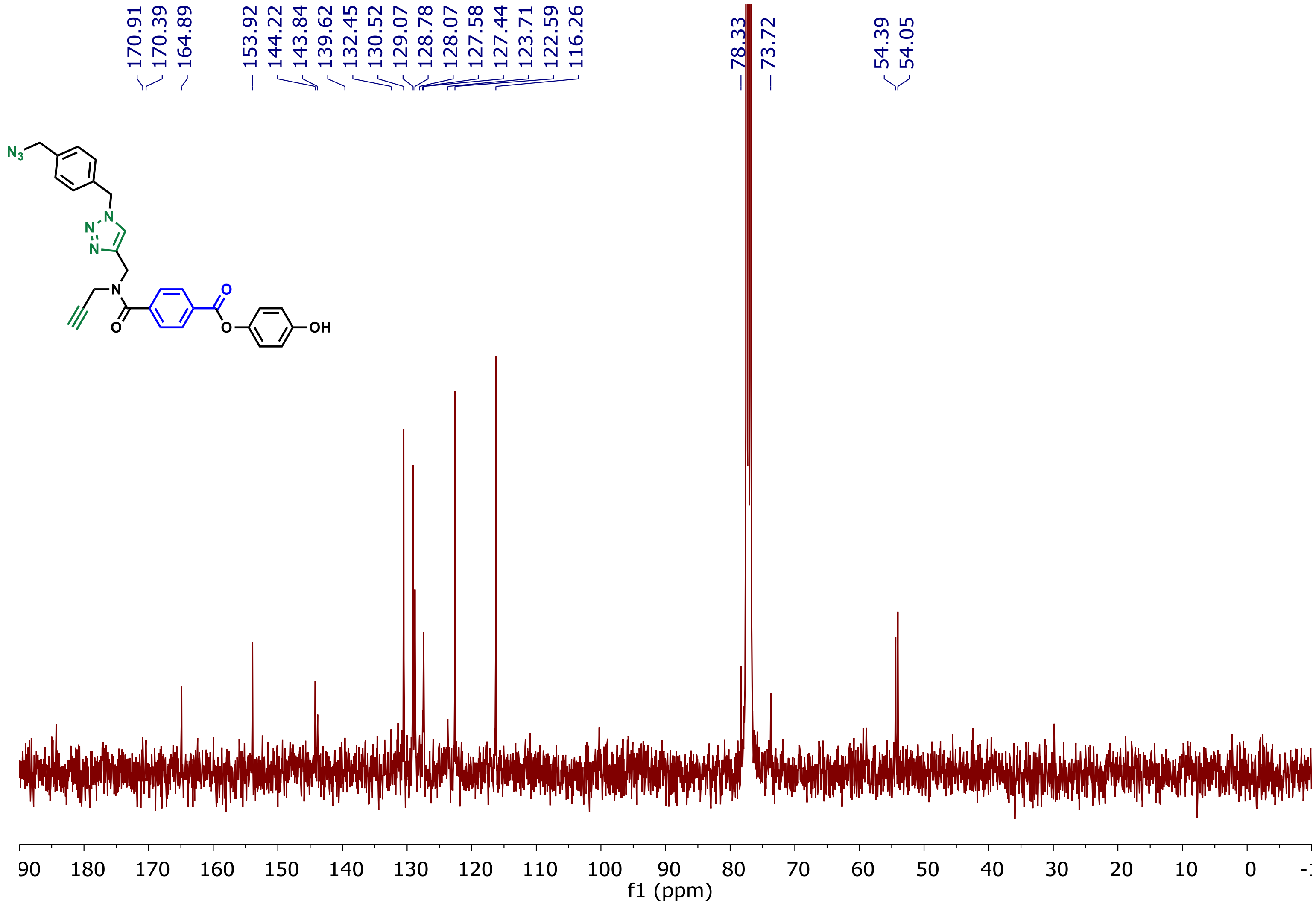

Pre-ZIP 8.

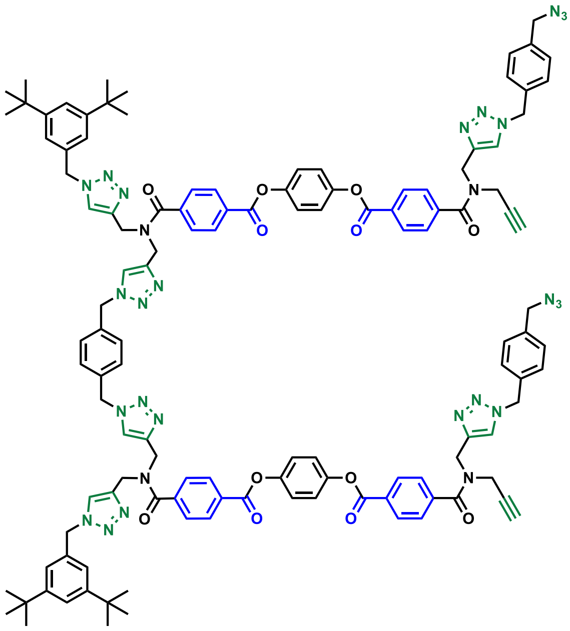

Compound **7**[S3] (0.072 g, 0.06 mmol), compound **6** (0.065 g, 0.12 mmol), EDC (0.026 g, 0.14 mmol) and DMAP (0.001 g, 0.006 mmol) were dissolved under N_2 atmosphere in dry CH_2Cl_2 (3 ml). The solution was stirred overnight under N_2 atmosphere at room temperature. The crude was diluted with EtOAc (5 mL) and washed with 5% aq. soln. HCl (3x), H_2O (1x) and brine. The organic phase was dried with $MgSO_4$ and concentrated *in vacuo*. The crude material was purified by flash column chromatography on silica gel (gradient from 0% to 4% of MeOH in CH_2Cl_2) to afford **pre-ZIP 8** (0.073 g, 54%) as a foam.

^{1}H NMR (400 MHz, CDCl$_3$): δ_H = 8.26 (m, 8H), 7.84 (d, 4H, *J* = 7.5 Hz), 7.77-7-67 (m, 8H), 7.65 and 7.58 (s, 2H, splitting is due to the existence of rotamers around the amide bond, only a singlet would be expected in the absence of rotamers), 7.44 (t, 2H, *J* = 1.5 Hz), 7.39-7.33 (m, 12H), 7.30 (s, 8H), 7.13 (d, 4H, *J* = 1.5 Hz), 5.54 (m, 12H), 4.89, 4.69 and 4.58 (bs, 12H, splitting is due to the existence of rotamers around the amide bond; only two singlets would be expected in the absence of rotamers), 4.39 (s, 4H), 4.39 and 4.12 (bs, 4H, splitting is due to the existence of rotamers around the amide bond; only a singlet would be expected in the absence of rotamers), 2.39 and 2.29 (bs, 2H, splitting is due to the existence of rotamers around the amide bond; only a singlet would be expected in the absence of rotamers), 1.33 (s, 36H).

^{13}C NMR (125.7 MHz, CDCl$_3$): δ_C = 170.7, 164.5, 152.1, 152.0, 148.6, 148.5, 144.2, 140.6, 135.5, 135.4, 133.7, 130.7, 130.6, 130.5, 129.1, 128.8, 128.1, 127.5, 124.0, 123.9, 123.6, 123.4, 123.1, 122.9, 122.8, 122.5, 78.4, 73.7, 55.1, 54.4, 54.0, 53.9, 44.2, 43.9, 40.2, 39.6, 35.1, 31.5.

HRMS (ES+): calcd for $C_{122}H_{119}N_{28}O_{12}$ 2167.9562 $[M+H]^+$, found 2167.9568 $[M+H]^+$.

FT-IR (ATR): ν_{max} 2960, 2931, 2102, 1736, 1633, 1260, 1172, 1072 and 749 cm^{-1}.

^{1}H-NMR (400 MHz, CDCl$_3$) pre-ZIP 8.

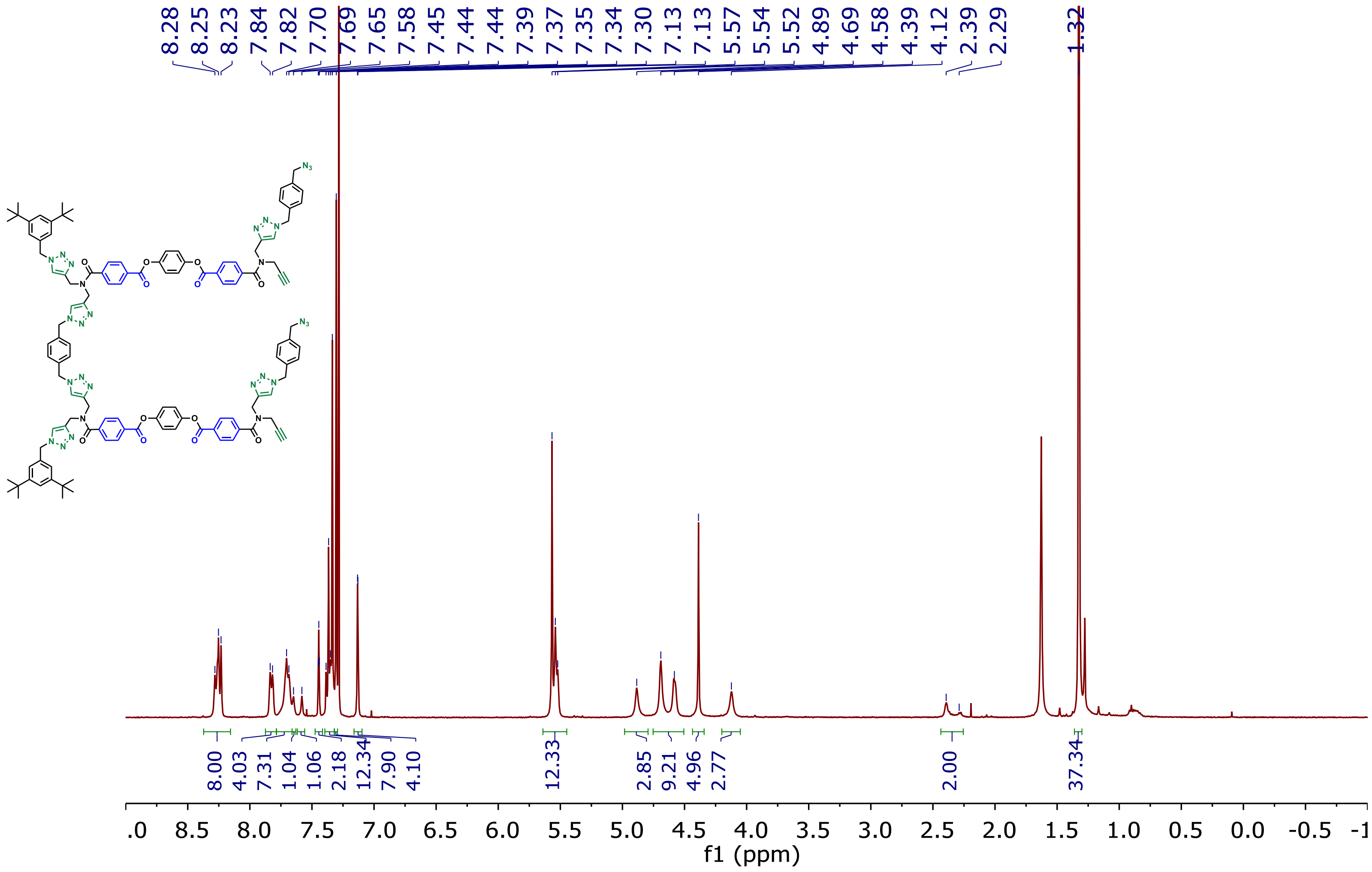

^{13}C-NMR (125.7 MHz, $CDCl_3$) pre-ZIP 8.

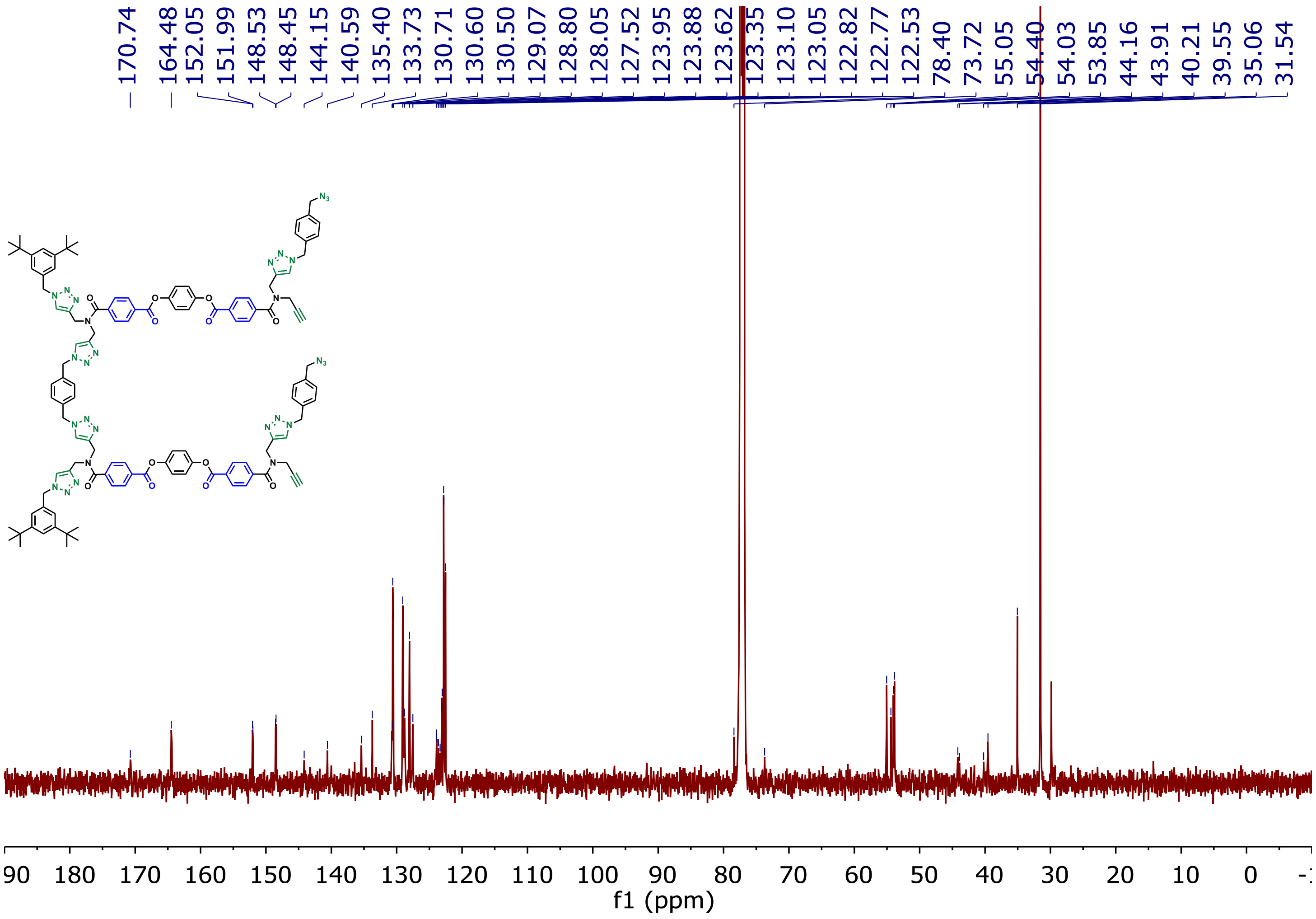

COSY ($CDCl_3$) pre-ZIP 8

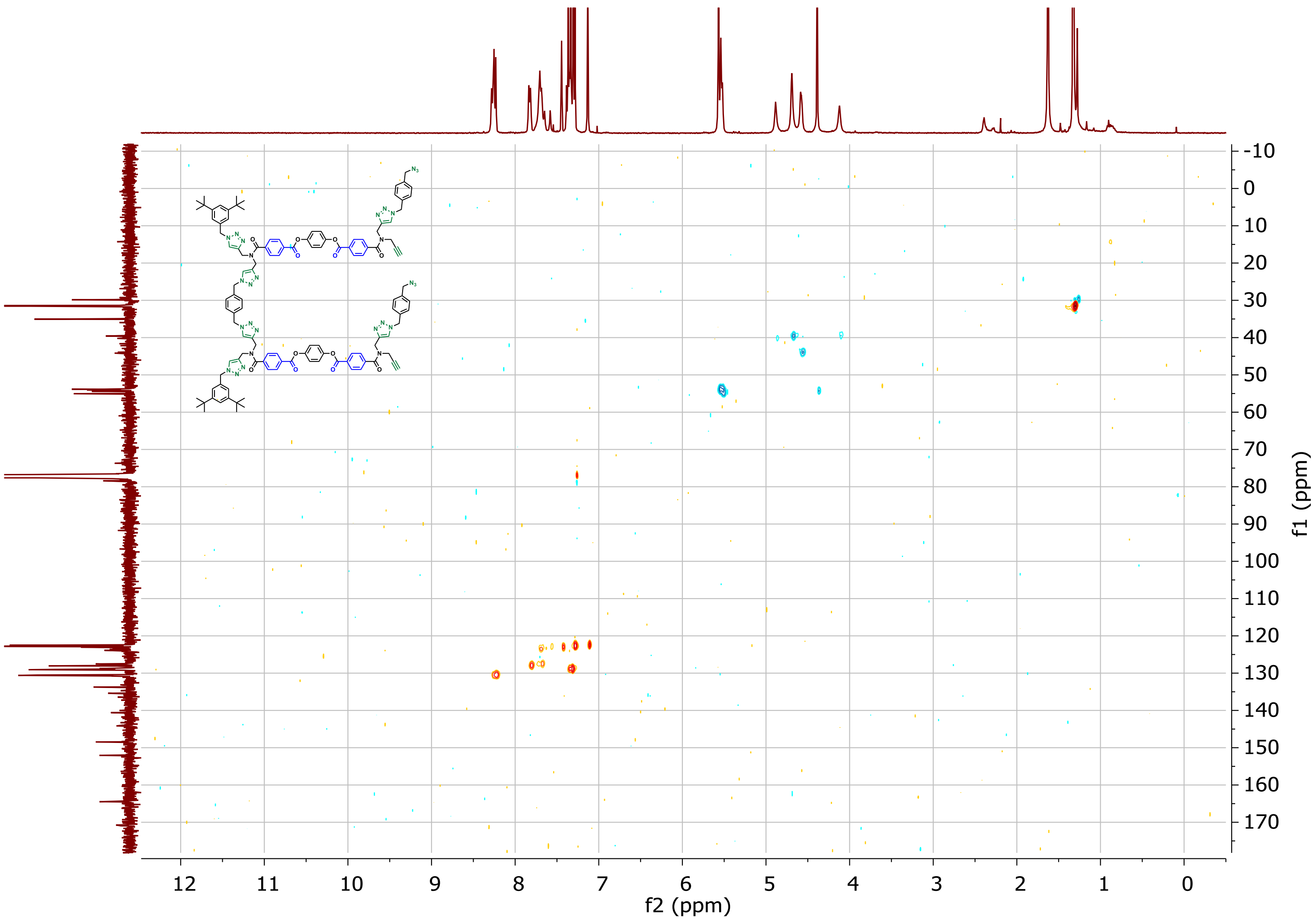

Compound 10.

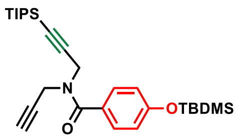

9[S2] (0.470 g, 1.86 mmol), EDC (0.464 g, 2.42 mmol) and DMAP (0.023 g, 0.19 mmol) were dissolved in dry CH_2Cl_2 (10 ml). Amine derivative **2** (0.532 g, 1.86 mmol) and TEA (0.260 mL, 1.86 mmol) were added under N_2 atmosphere and the solution was left stirring at room temperature for 2 h. The crude was diluted with EtOAc (50 mL) and washed with 5% aq. soln. HCl (3x), H_2O (1x) and brine. The organic phase was dried with $MgSO_4$ and concentrated *in vacuo*. The crude material was purified by flash column chromatography on silica gel (gradient from 0% to 10% of EtOAc in Pet. Ether) to afford **10** (0.855 g, 95 %) as a clear oil.

^{1}H NMR (400 MHz, CDCl$_3$): δ_H = 7.51 (d, 2H, *J* = 8.5 Hz), 6.85 (d, 2H, *J* = 8.5 Hz), 4.38 and 4.35 (bs, 4H, splitting is due to the existence of rotamers around the amide bond; only a singlet would be expected in the absence of rotamers), 2.27 (bs, 1H) 1.09 (m, 21H), 0.99 (s, 9H), 0.21 (s, 6H).

^{13}C NMR (100.6 MHz, CDCl$_3$): δ_C = 170.7, 157.7, 129.2, 127.5, 119.9, 101.8, 78.5, 76.2, 72.4, 25.6, 18.6, 18.2, 11.1, -4.4. N-CH_2 peaks are not listed due to broadness caused by the existence of rotamers.

HRMS (ES+): calcd for $C_{28}H_{46}NO_2Si_2$ 484.3067 $[M+H]^+$, found 484.3062 $[M+H]^+$.

FT-IR (ATR): ν_{max} 2941, 2864, 1650, 1605, 1510, 1253, 911 and 841 cm^{-1}.

^{1}H-NMR (400 MHz, $CDCl_3$) Compound 10.

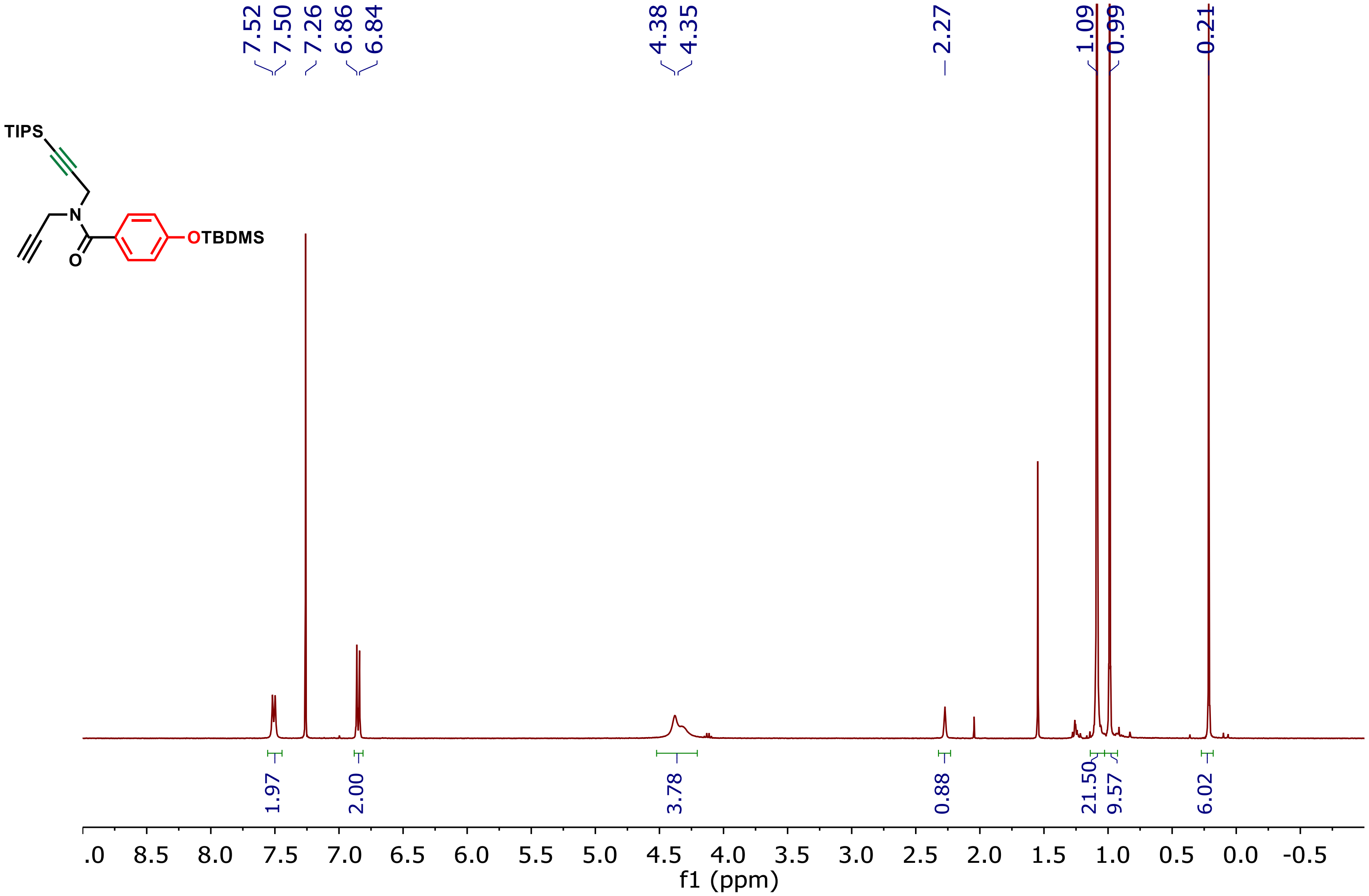

^{13}C-NMR (100.6 MHz, $CDCl_3$) Compound 10.

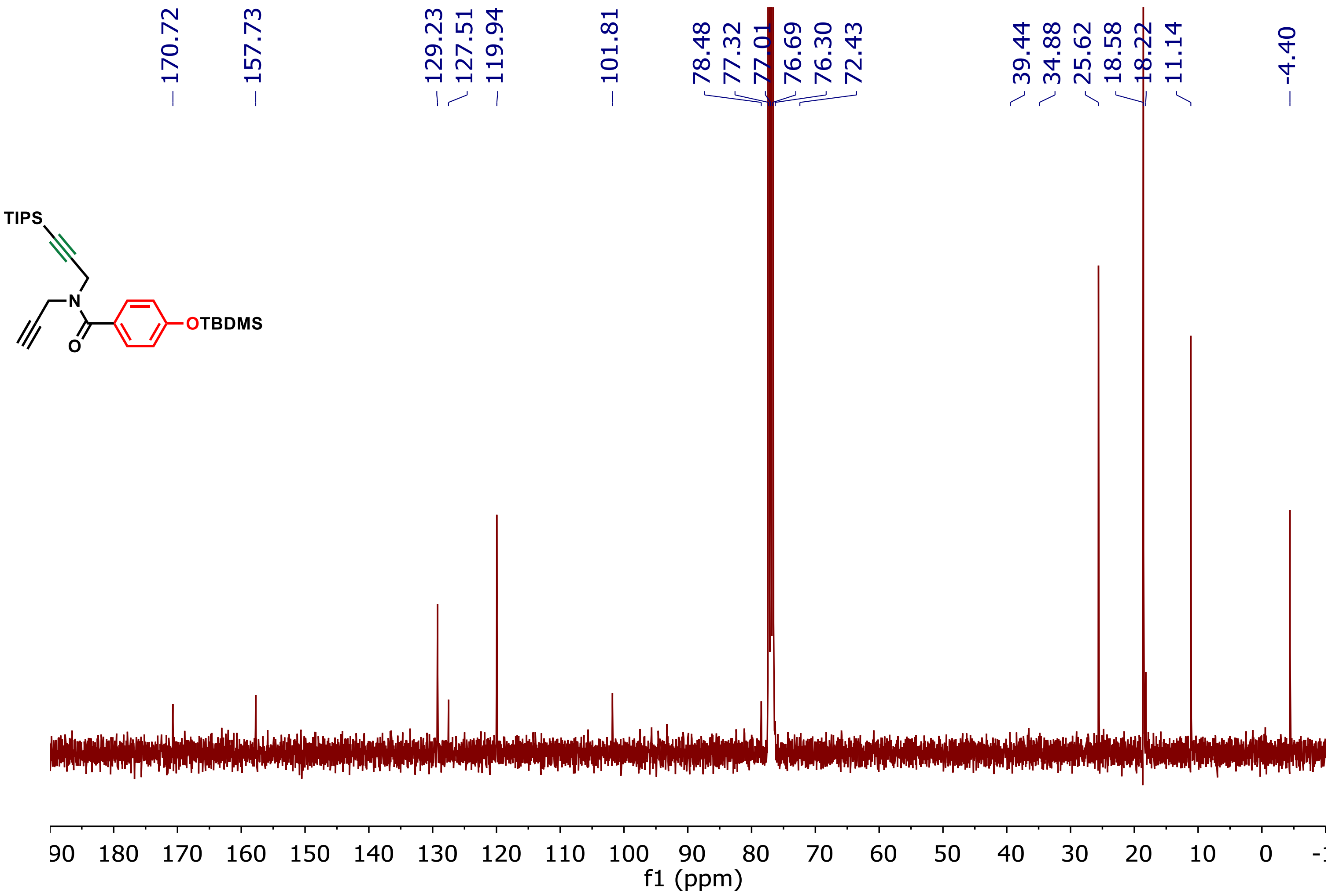

Compound S1.

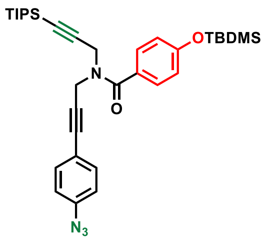

1-Azido-4-iodobenzene[S5] (0.109 g, 0.45 mmol), $Pd(PPh_3)_4$ (0.005 g, 0.006 mmol) and CuI (0.003 g, 0.013 mmol) were mixed under N_2 and THF/TEA 1:1 (5 mL) was added. Then, 10 (0.154 g, 0.32 mmol) dissolved in THF (1 mL) was added. After stirring at 30 °C for 2 h, the reaction was quenched with 0.1 N HCl soln. and extracted with EtOAc (2x). The combined organic phase was washed with 0.02 M EDTA soln. and brine, dried over $MgSO_4$ and the solvent evaporated. The crude material was purified by flash column chromatography on silica gel (gradient from 0% to 5% of EtOAc in Pet. Ether) to afford **S1** (0.118 g, 62%) as a clear oil, recovering starting material **10** (0.015 g, 10%).

^{1}H NMR (400 MHz, CDCl$_3$): δ_H = 7.53 (d, 2H, *J* = 8.5 Hz), 7.43 (d, 2H, *J* = 8.5 Hz), 6.97 (d, 2H, *J* = 8.5 Hz), 6.86 (d, 2H, *J* = 8.5 Hz), 4.60 (bs, 1H), 4.37 (bs, 1H), 1.09 (m, 21H), 0.99 (s, 9H), 0.22 (s, 6H).

^{13}C NMR (100.6 MHz, CDCl$_3$): δ_C = 170.9, 157.9, 161.0, 133.5, 129.4, 127.8, 125.1, 120.1, 119.1, 102.1, 88.7, 84.3, 80.1, 25.8, 18.8, 18.4, 11.3, -4.2. N-CH_2 peaks are not listed due to broadness caused by the existence of rotamers.

HRMS (ES-): calcd for $C_{34}H_{49}N_4O_2Si_2$ 601.3394 $[M+H]^+$, found 601.3432 $[M+H]^+$.

FT-IR (ATR): ν_{max} 2928, 2864, 2128, 2096, 1646, 1605, 1506, 1255, 912 and 840 cm^{-1}.

^{1}H-NMR (400 MHz, $CDCl_3$) Compound S1.

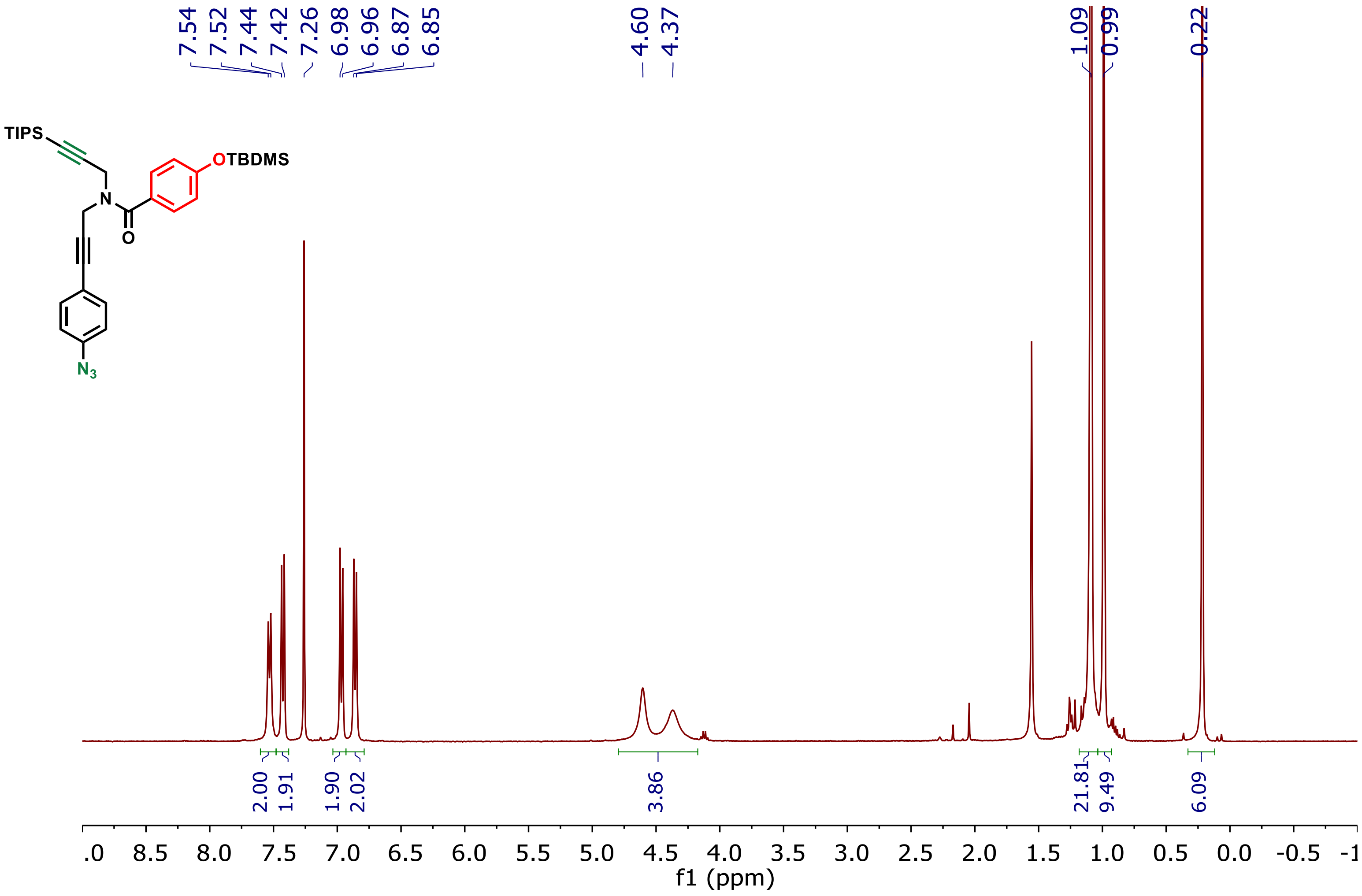

^{13}C-NMR (100.6 MHz, $CDCl_3$) Compound S1.

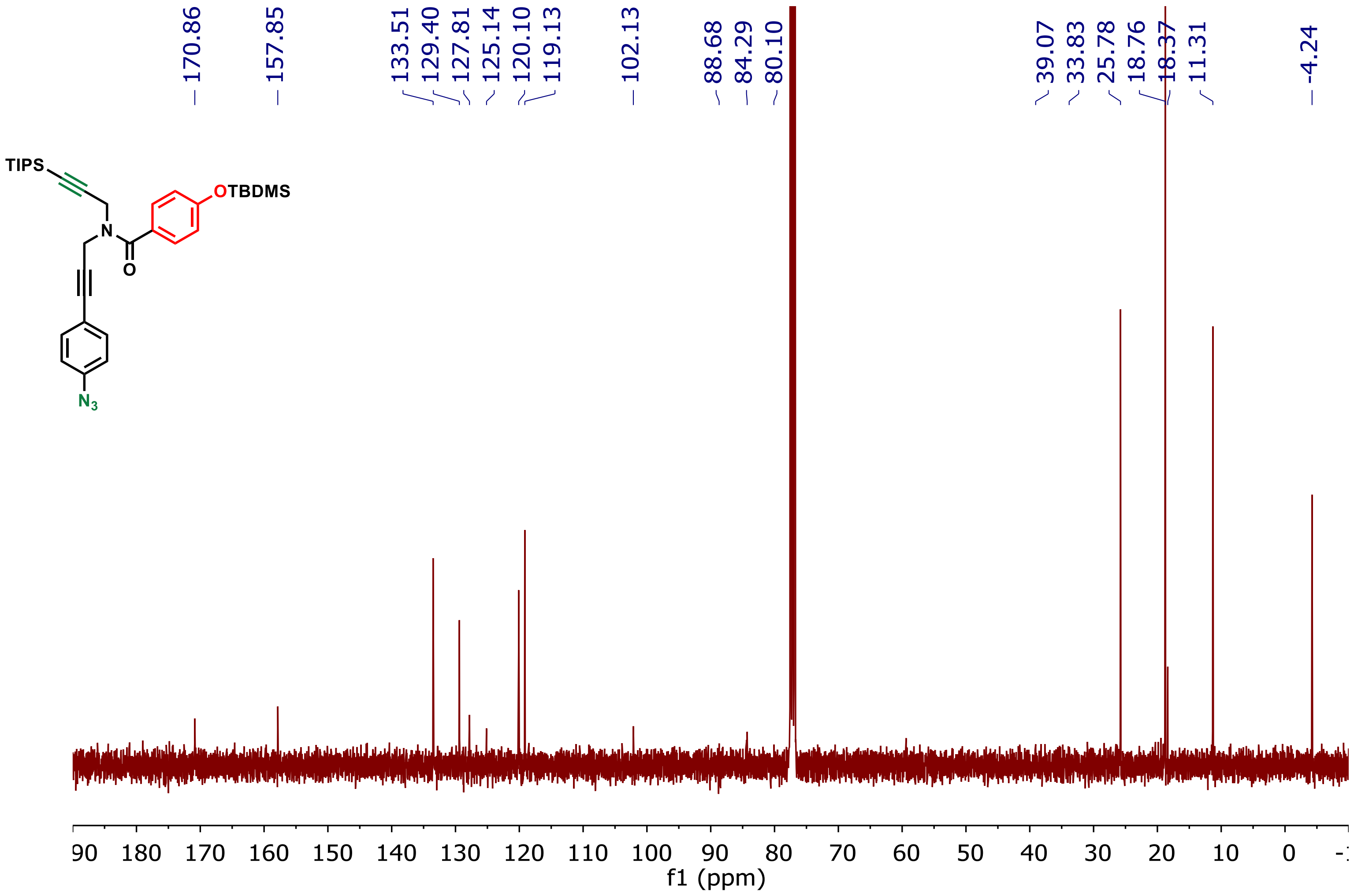

Compound 11.

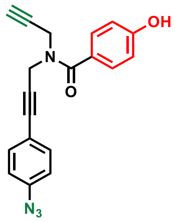

Compound **S1** (0.049 g, 0.08 mmol) was dissolved in dry THF (1 mL) under N_2 atmosphere. TBAF (1M in THF, 0.16 mL, 0.16 mmol) was added and the reactions stirred at room temperature for 15 min. THF was evaporated and the crude dissolved in EtOAc and washed with 0.1 N HCl soln. (3x), H_2O (1x) and brine. The obtained residue was dried under high vacuum for 3 h, yielding **11** as a light-yellow foam (0.027 g, quantitative).

^{1}H NMR (400 MHz, DMSO-*d*$_6$): δ_H = 10.0 (bs, 1H), 7.51 (d, 2H, *J* = 8.5 Hz), 7.40 (d, 2H, *J* = 8.5 Hz), 7.13 (d, 2H, *J* = 8.5 Hz), 6.84 (d, 2H, *J* = 8.5 Hz), 4.46 (bs, 1H), 4.28 (bs, 1H), 3.37 (s, 1H).

^{1}H NMR (100.6 MHz, DMSO-*d*$_6$): δ_C = 169.9, 159.4, 139.9, 133.2, 129.2, 124.9, 119.5, 118.4, 115.1, 85.0, 83.1, 79.3, 75.3, 48.6.

HRMS (ES+): calcd for $C_{19}H_{15}N_4O_2$ 331.1195 $[M+H]^+$, found 331.1216 $[M+H]^+$.

FT-IR (ATR): ν_{max} 3255, 2922, 2852, 2130, 2098, 1631, 1601, 1514 and 1276 cm^{-1}.

.

^{1}H-NMR (400 MHz, DMSO-d_6) Compound 11.

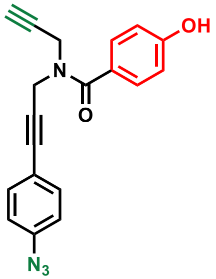

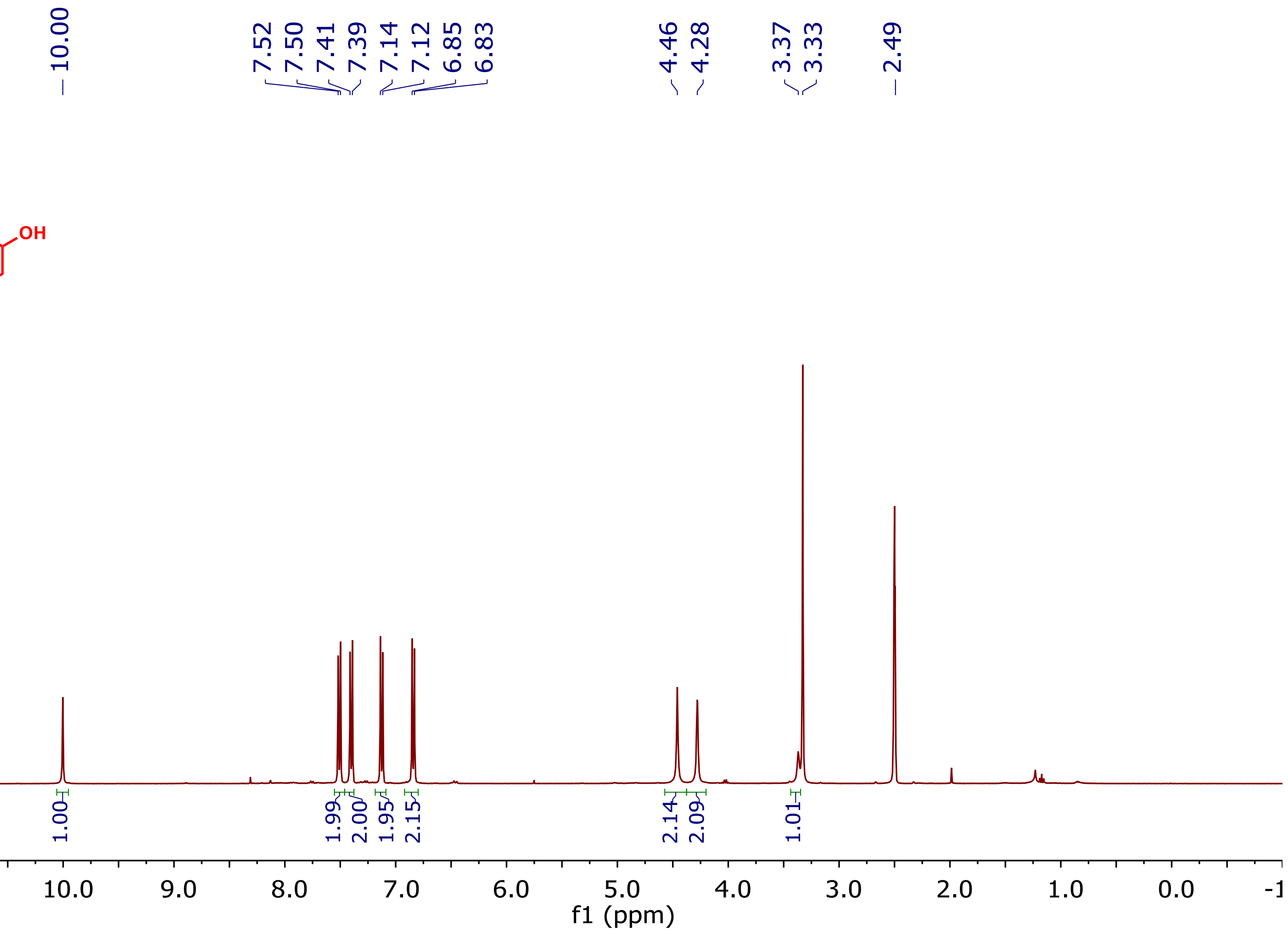

^{1}H-NMR (100.6 MHz, DMSO-d_6) Compound 11.

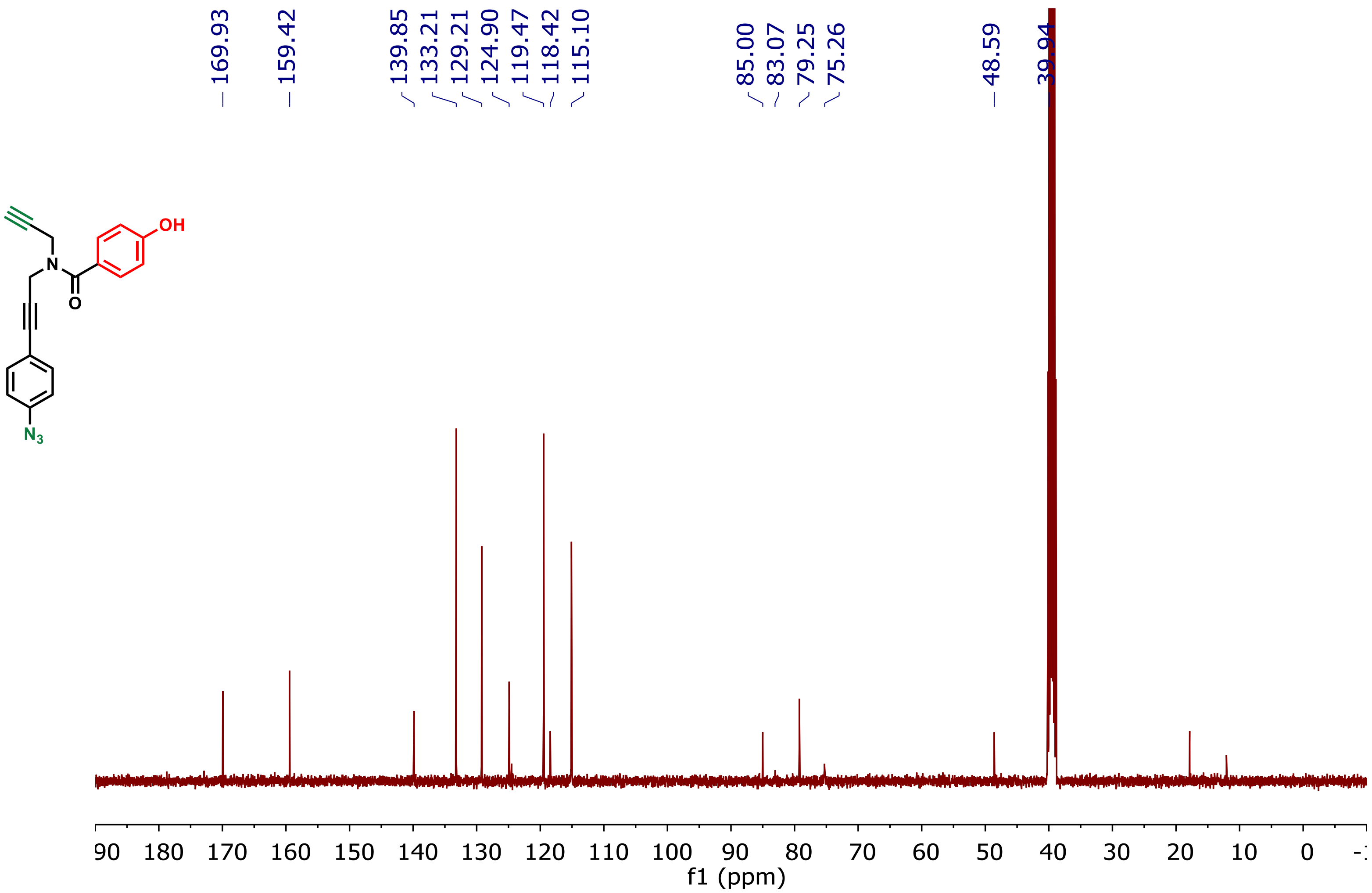

Compound 13.

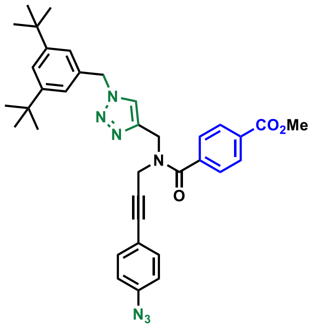

1-Azido-4-iodobenzene[S5] (0.051 g, 0.21 mmol), $Pd(PPh_3)_4$ (2 mg, 0.002 mmol) and CuI (0.3 mg, 0.002 mmol) were mixed under N_2 and THF/TEA 1:1 (4 mL) was added. Then, **12** (0.086 g, 0.17 mmol) dissolved in THF (1 mL) was added. After stirring at 30 °C for 45 min, the reaction was quenched with 0.1 N HCl soln. and extracted with EtOAc (2x). The combined organic phase was washed with 0.02 M EDTA soln. and brine, dried over $MgSO_4$ and the solvent evaporated. The crude material was purified by flash column chromatography on silica gel (gradient from 0% to 40% of EtOAc in Pet. Ether) to afford compound **13** (0.089 g, 84%) as a brown oil.

^{1}H NMR (400 MHz, $CDCl_3$): δ_H = 8.09 (d, 2H, *J* = 7.5 Hz), 7.62 (m, 3H), 7.42 (m, 3H), 7.10 (s, 2H), 6.99 (d, 2H, *J* = 7.5 Hz), 5.50 (s, 2H), 4.90, 4.67, 4.58 and 4.28 (bs, 4H, splitting is due to the existence of rotamers around the amide bond; only two singlets would be expected in the absence of rotamers), 3.93 (s, 3H), 1.30 (s, 18H).

^{13}C NMR (125.7 MHz, $CDCl_3$): δ_C = 170.2, 166.4, 151.9, 143.8, 140.6, 139.4, 133.7, 133.5, 132.9, 129.9, 127.3, 123.6, 123.0, 122.5, 119.2, 84.5, 84.0, 55.0, 52.5, 40.3, 34.5, 35.0, 31.5.

HRMS (ES+): calcd for $C_{36}H_{40}N_7O_3$ 618.3193 $[M+H]^+$, found 618.3201 $[M+H]^+$.

FT-IR (ATR): ν_{max} 2954, 2127, 2095, 1723, 1637, 1275, 1248, 1108 and 750 cm^{-1}.

^{1}H-NMR (400 MHz, $CDCl_3$) Compound 13.

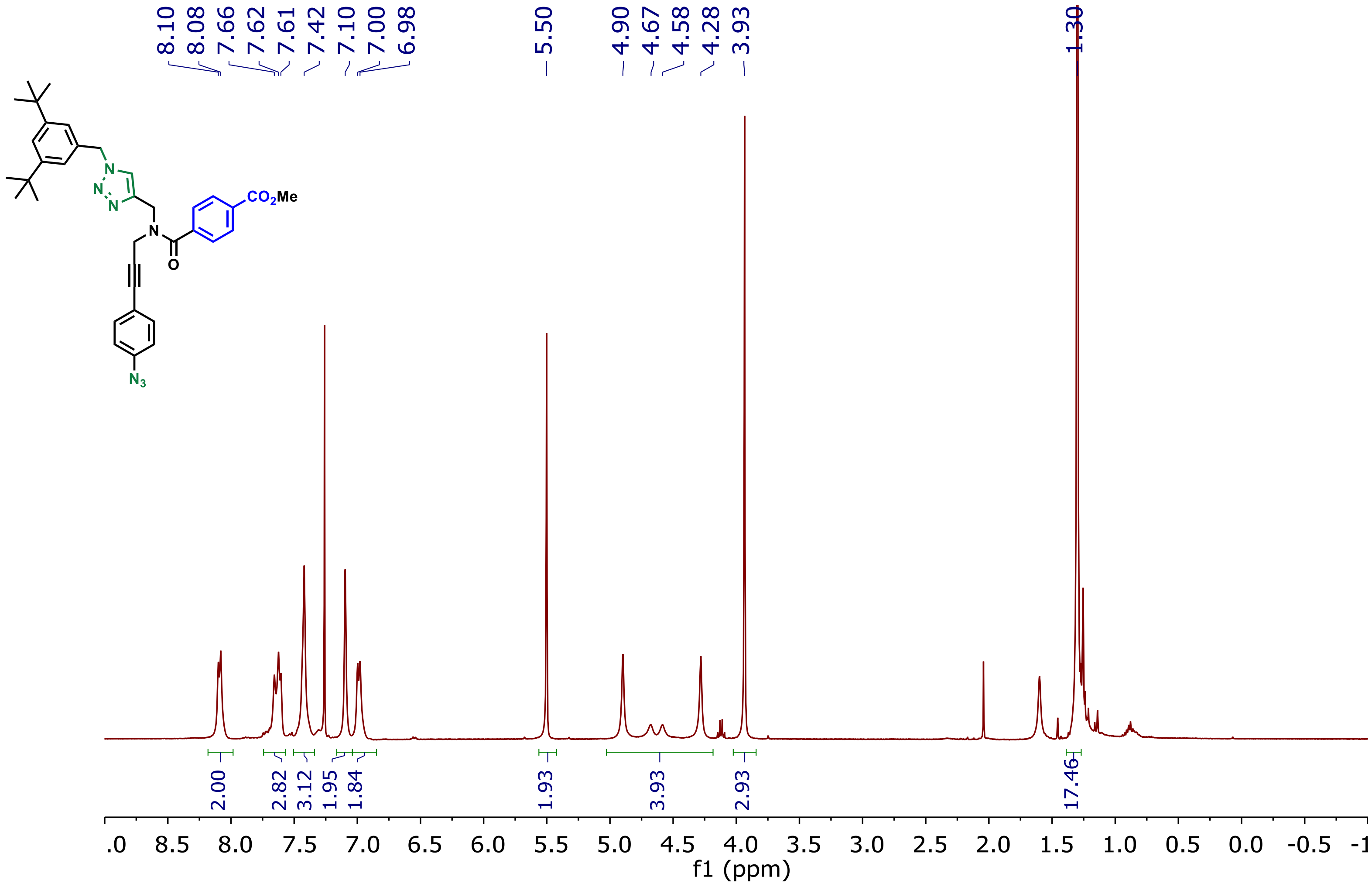

^{13}C-NMR (125.7 MHz, $CDCl_3$) Compound 13.

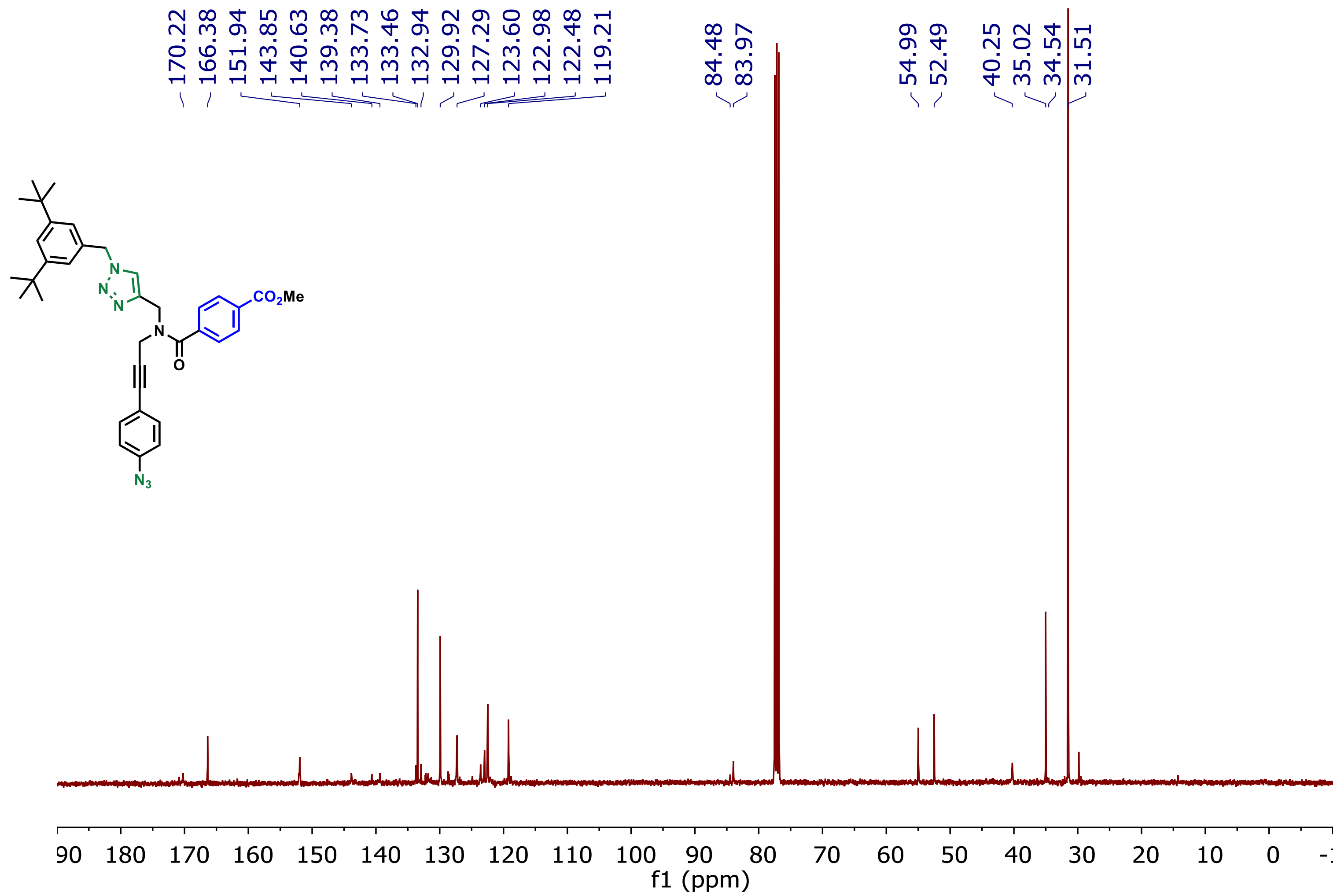

Compound S2.

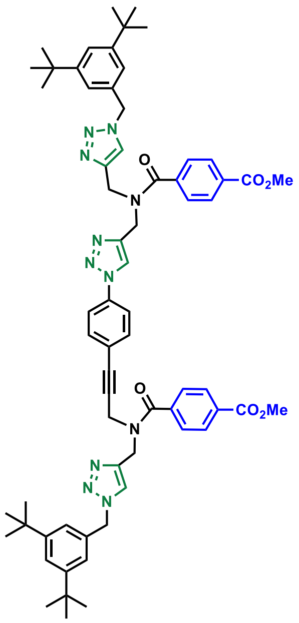

Compound **13** (0.064 g, 0.10 mmol) and compound **9** (0.052 g, 0.10 mmol) were dissolved in anhydrous THF (6 mL) under N_2 atmosphere. Then, TBTA (5 mg, 0.01 mmol) and $Cu(CH_3CN)_4PF_6$ (3.7 mg, 0.01 mmol) were dissolved in THF (2 mL) and this solution added to the reaction mixture. After 16 h of stirring at room temperature, the solvent was evaporated and the crude purified by flash column chromatography on silica gel (gradient from 10% to 100% of EtOAc in Pet. Ether) to afford **S2** (0.113 g, 97 %) as a foam. A minor impurity is observed in the NMR spectra.

^{1}H NMR (400 MHz, CDCl$_3$): δ_H = 8.18 (m, 1H), 8.09 (m, 4H), 7.76 (d, 4H, *J* = 8.0 Hz), 7.62 (m, 6H), 7.43 (s, 2H), 7.11 (s, 4H), 5.51 (s, 4H), 4.92, 4.77, 4.74, 4.65, 4.59, 4.33 (bs, 8H), 3.94 (s, 3H), 3.92 (s, 3H), 1.31 (s, 36H).

^{13}C NMR (100.6 MHz, CDCl$_3$): δ_C = 176.1, 166.5, 166.5, 152.0, 143.8, 143.1, 133.7, 133.5, 1230.0, 127.8, 127.3, 125.1, 123.0, 122.5, 120.4, 96.1, 95.1, 55.1, 52.5, 44.5, 40.7, 39.2, 35.0, 34.5, 31.5.

HRMS (ES+): calcd for $C_{66}H_{76}N_{11}O_6$ 1118.5980 $[M+H]^+$, found 1118.6074 $[M+H]^+$.

FT-IR (ATR): ν_{max} 2955, 1725, 1636, 1434, 1277, 1248 and 751 cm^{-1}.

^{1}H-NMR (400 MHz, $CDCl_3$) Compound S2.

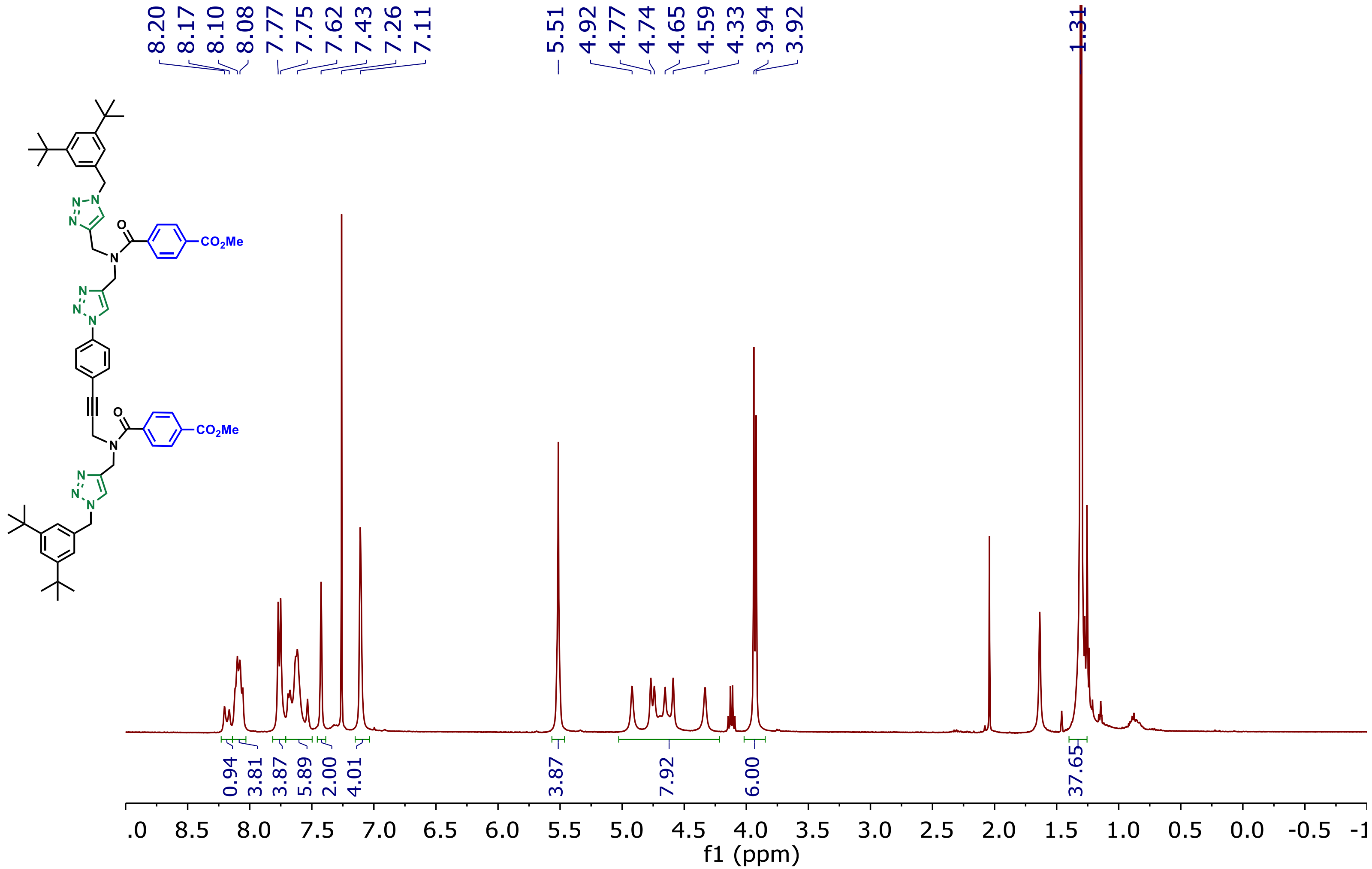

^{13}C-NMR (100.6 MHz, $CDCl_3$) Compound S2.

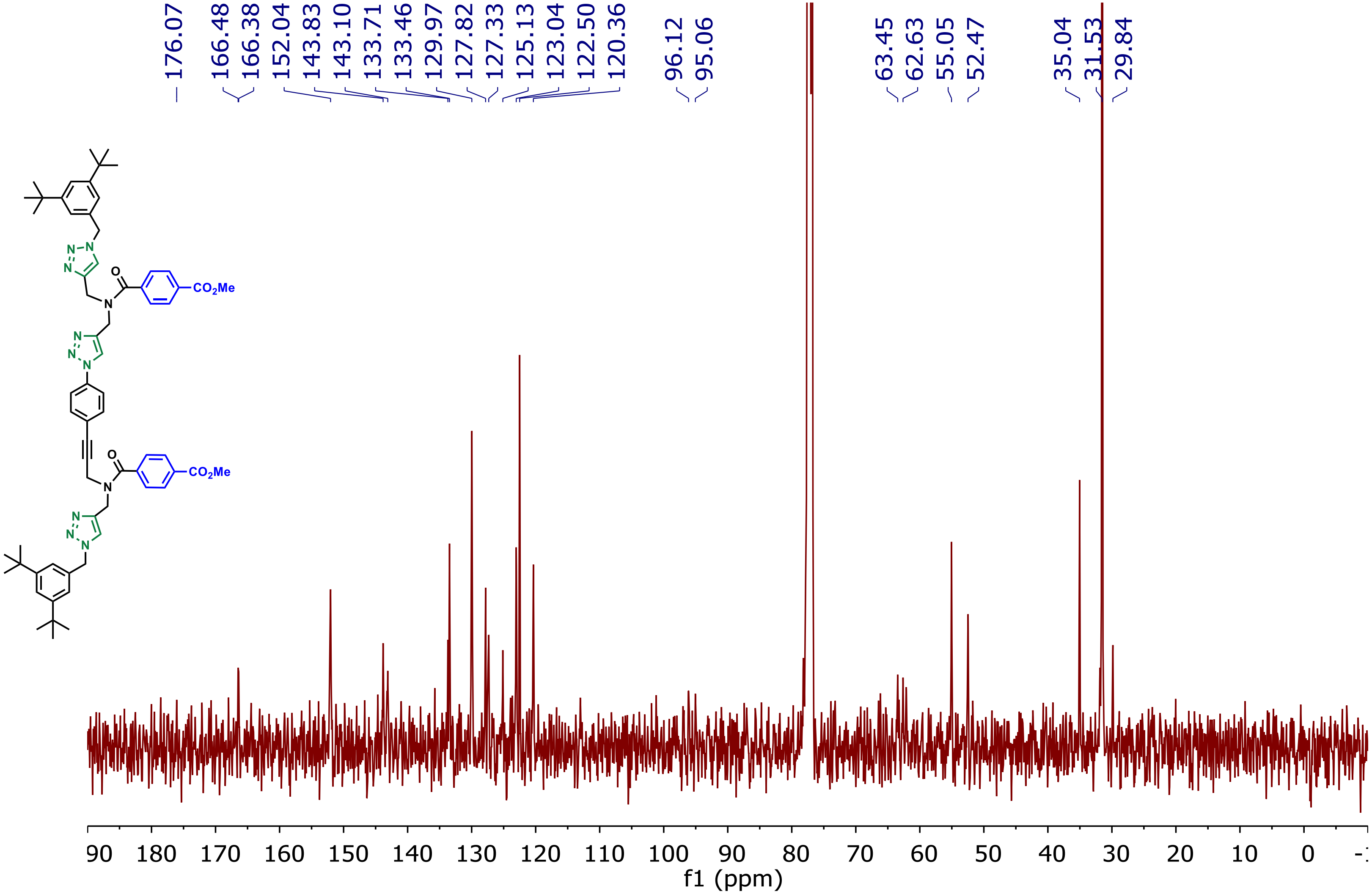

COSY ($CDCl_3$) Compound S2.

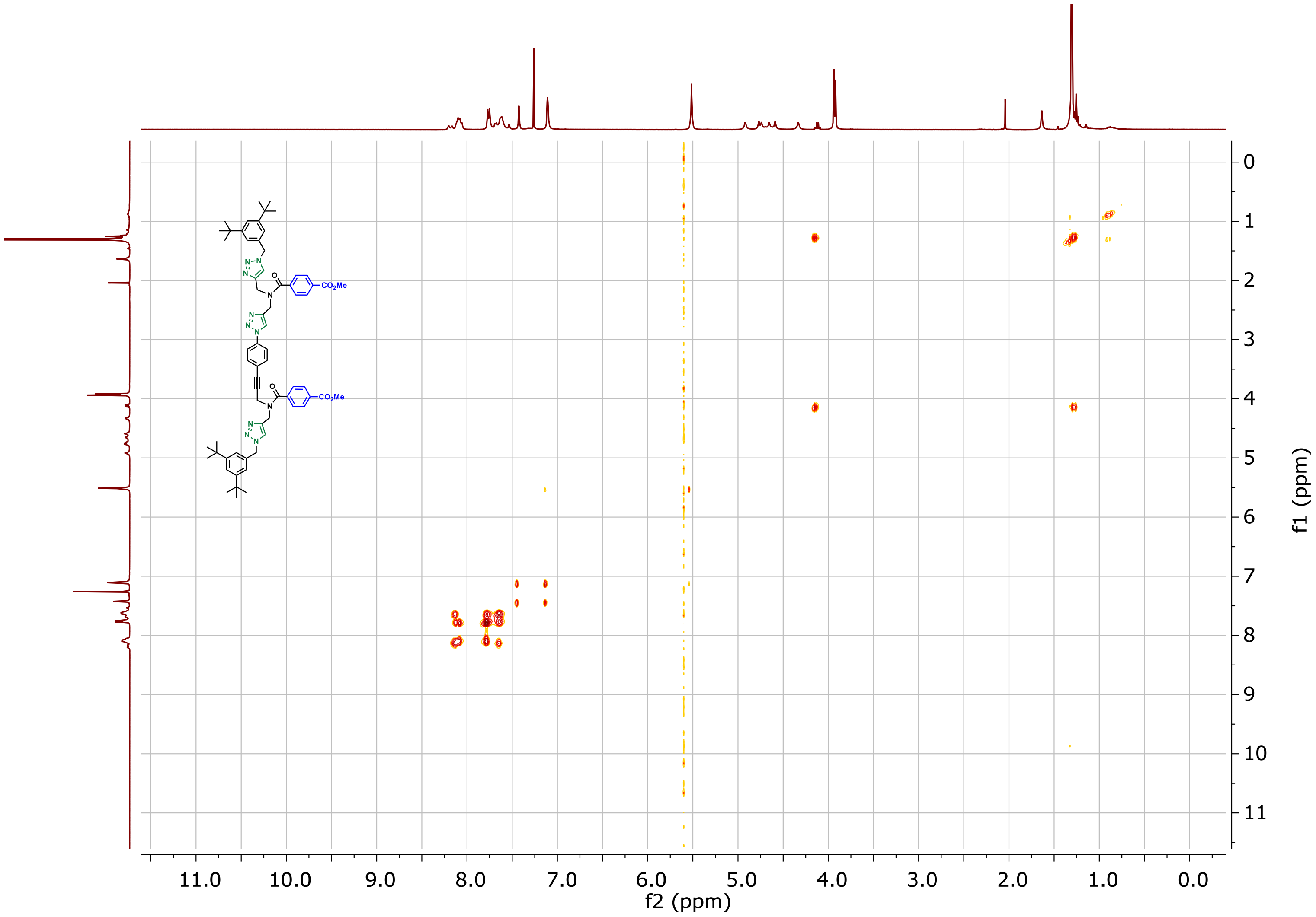

HSQC (CDCl$_3$) Compound S2

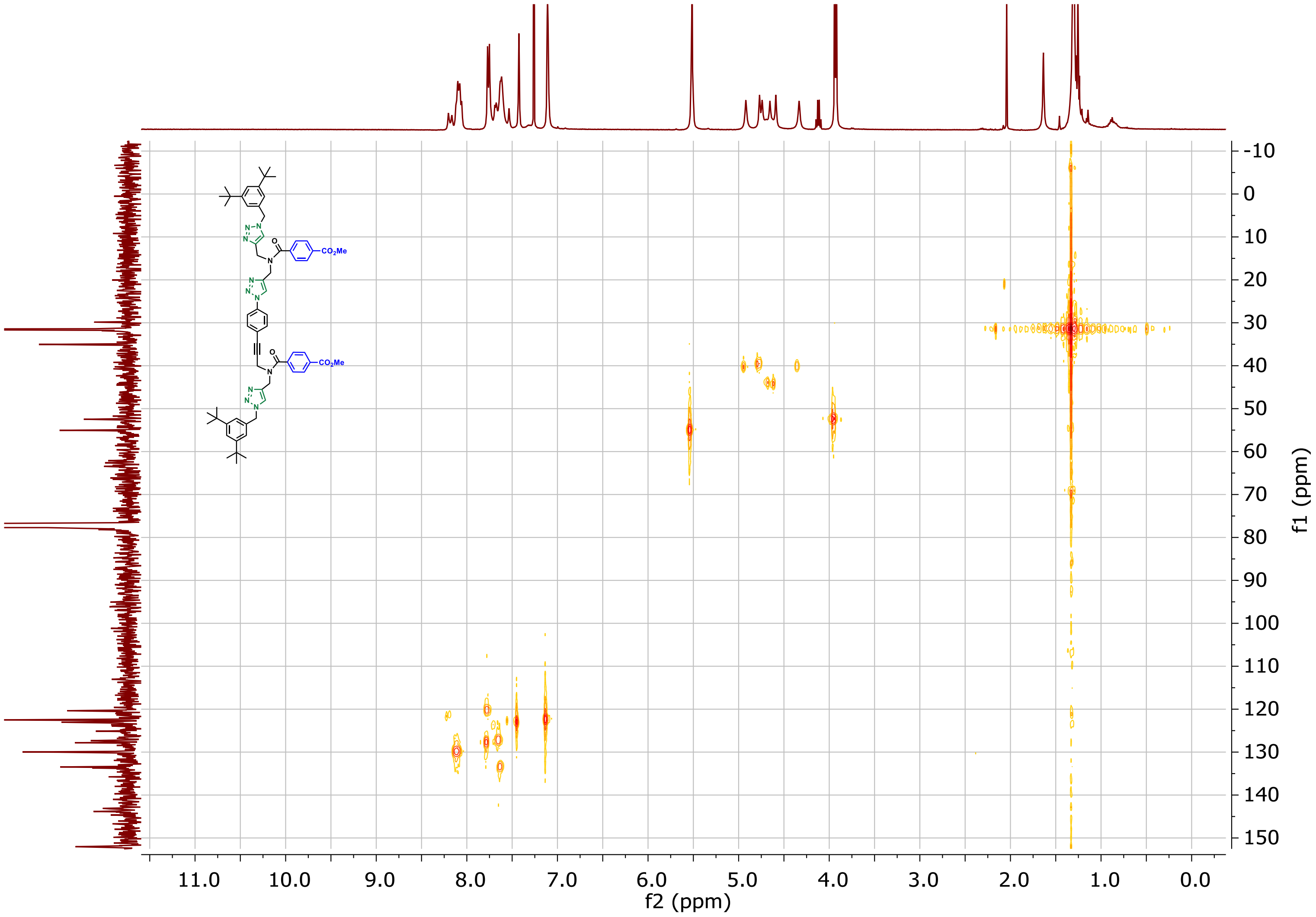

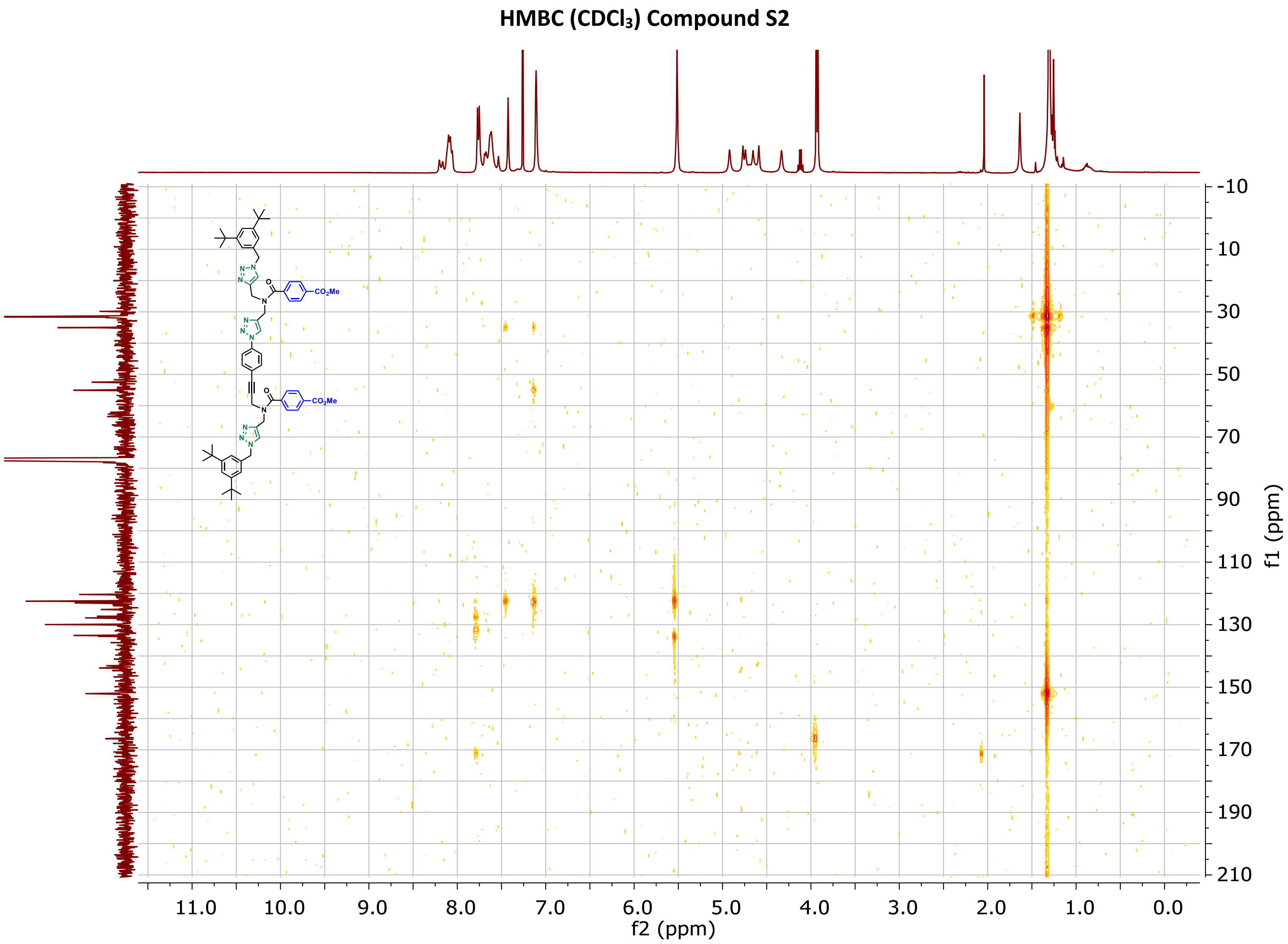
HMBC (CDCl$_3$) Compound S2
CO$_2$Me
CO$_2$Me
f2 (ppm)
f1 (ppm)

Compound 14.

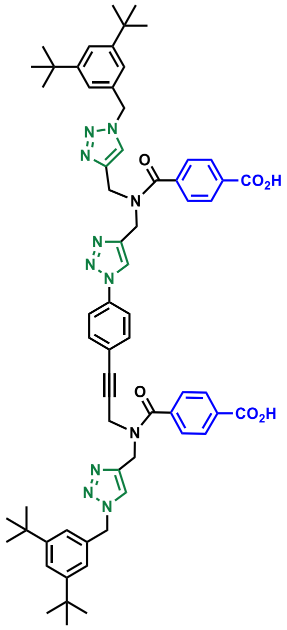

Compound **S2** (0.103 g, 0.09 mmol) was dissolved in MeOH/THF 1:1 (2 mL) and 2 N NaOH soln. (0.275 mL, 0.55 mmol) was added. After 16 h of stirring at room temperature, the solvent was evaporated and crude dissolved in H_2O and acidified with 1 N HCl soln. to pH 2-3. The aqueous phase was extracted with EtOAc (3x) and the combined organic phase was washed with brine, dried over $MgSO_4$ and evaporated to dryness. The obtained yellow solid, **14** (0.100 g, quantitative), was used without further purification.

Melting point: 146-148 ^{o}C.

^{1}H NMR (400 MHz, $CDCl_3$): δ_H = 8.34 (m, 1H), 8.09 (m, 4H), 7.74 (m, 6H), 7.65 (d, 2H, J = 7.5 Hz), 7.53 (d, 2H, J = 7.5 Hz), 7.42 (s, 2H), 7.12 (s, 4H), 5.52 (s, 4H), 4.95, 4.81, 4.70, 4.65 and 4.33 (bs, 8H, splitting is due to the existence of rotamers around the amide bond; only three singlets would be expected in the absence of rotamers), 1.29 (s, 36H).

^{13}C NMR (100.6 MHz, $CDCl_3$): δ_C = 176.4, 171.4, 169.8, 166.6, 133.5, 133.4, 133.4, 130.5, 130.5, 127.7, 127.4, 123.1, 123.1, 122.6, 120.3, 85.6, 82.9, 55.1, 35.0, 33.8, 31.5. N-CH_2 peaks are not listed due to broadness caused by the existence of rotamers.

HRMS (ES+): calcd for $C_{64}H_{72}N_{11}O_8$ 1090.5667 $[M+H]^+$, found 1090.5776 $[M+H]^+$.

FT-IR (ATR): ν_{max} 2962, 1713, 1635, 1603, 1247 and 735 cm^{-1}.

^{1}H-NMR (400 MHz, $CDCl_3$) Compound 14.

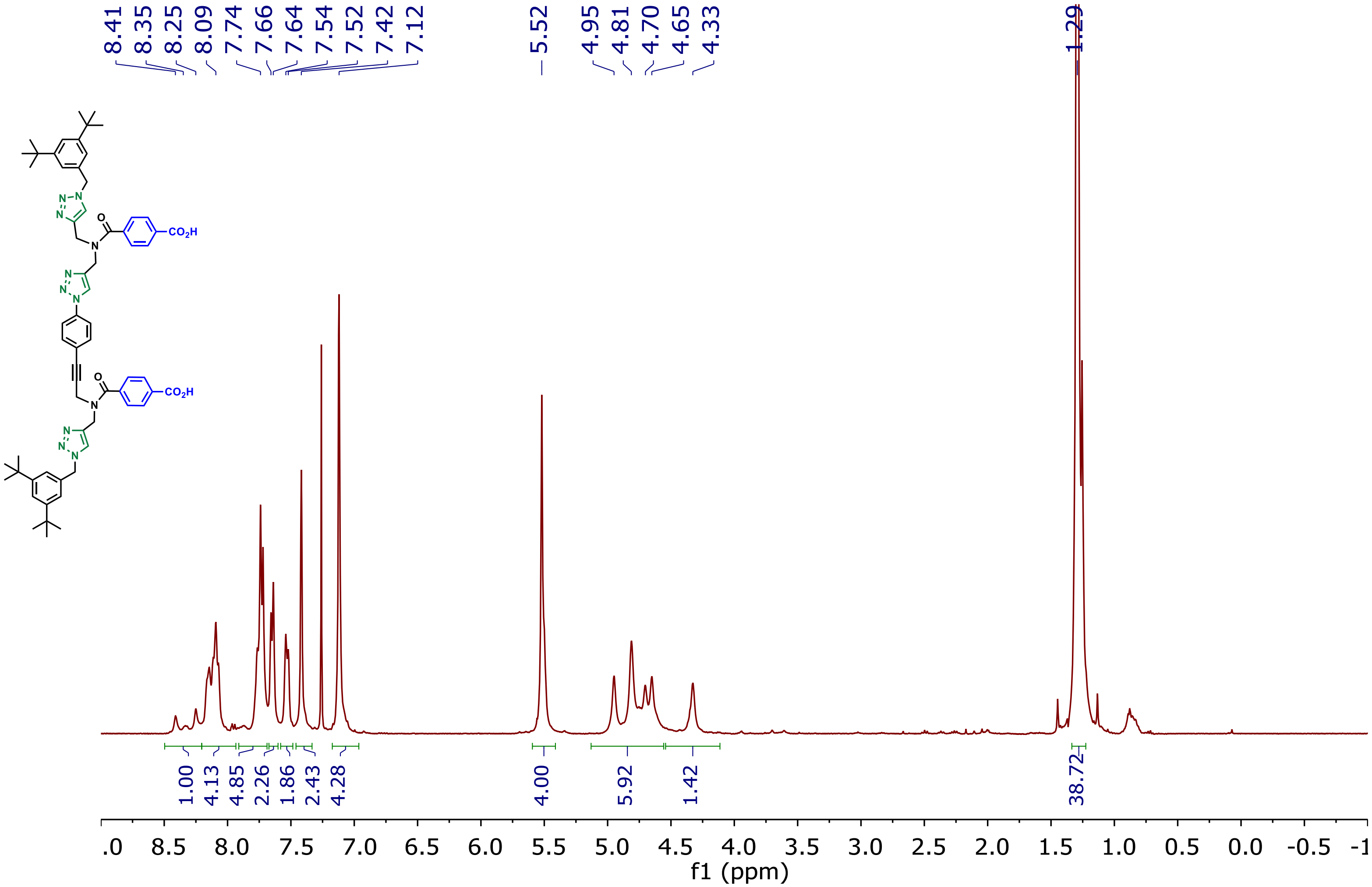

^{13}C-NMR (100.6 MHz, $CDCl_3$) Compound 14.

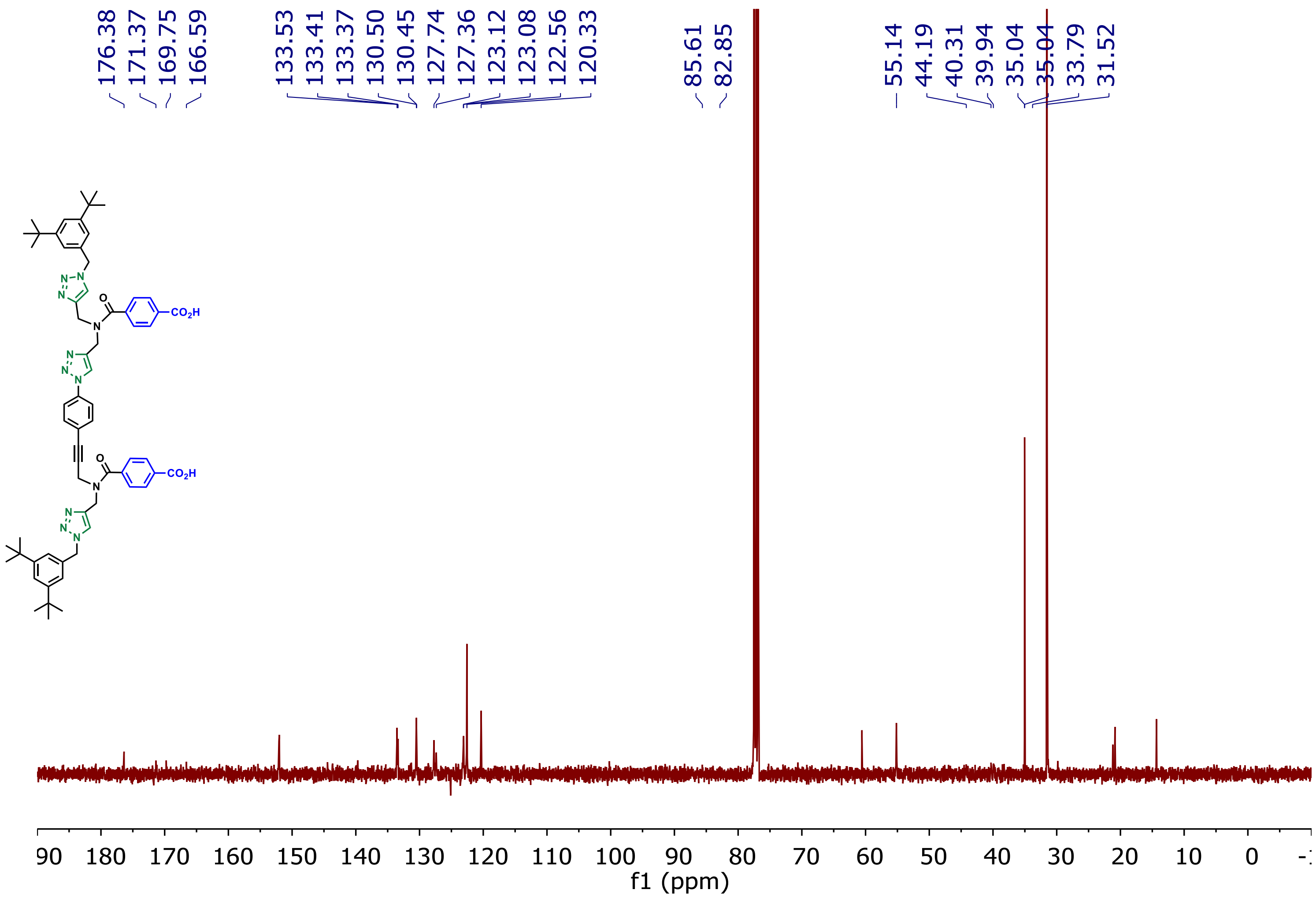

Pre-ZIP 15.

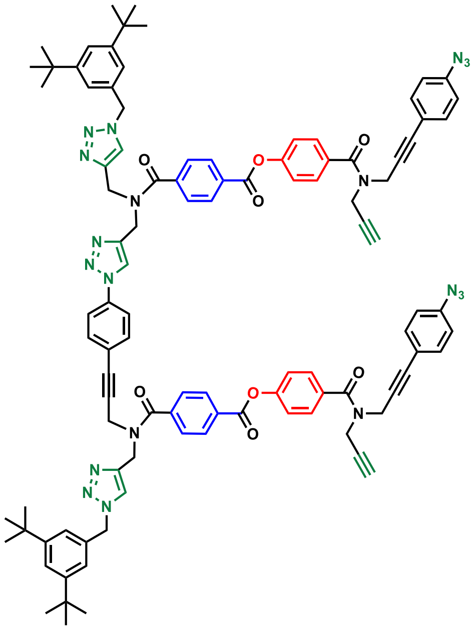

A solution of **14** (0.078 g, 0.072 mmol), **11** (0.052 g, 0.158 mmol), EDC (0.041 g, 0.215 mmol) and DMAP (3 mg, 0.022 mmol) in dry CH_2Cl_2 (5 mL) under N_2 atmosphere was stirred at room temperature for 16 h. The reaction was diluted with EtOAc (5 mL) and washed with 0.1 N HCl soln. (3x), H_2O (1x) and brine. The organic phase was dried with $MgSO_4$ and concentrated *in vacuo*. The crude material was purified by flash column chromatography on silica gel (gradient from 0% to 4% of MeOH in CH_2Cl_2) to afford **15** (0.086 g, 70 %) as a light-yellow foam.

^{1}H NMR (400 MHz, CDCl$_3$): δ_H = 8.24 (m, 6H), 7.98 (m, 2H), 7.86 (d, 2H, *J* = 8.0 Hz), 7.73 (m, 9H), 7.60 (m, 3H), 7.43 (m, 5H), 7.31 (m, 4H), 7.12 (s, 4H), 6.97 (d, 2H, *J* = 8.5 Hz), 5.52 (s, 4H), 4.94-4.37 (m, 16H), 2.36 and 2.04 (bs, 2H), 1.30 (s, 36H).

^{13}C NMR (100.6 MHz, CDCl$_3$): δ_C = 170.8, 170.0, 164.1, 164.0, 152.4, 151.9, 144.5, 143.6, 142.8, 140.6, 140.5, 136.7, 133.6, 133.4, 132.4, 130.6, 130.5, 129.0, 128.1, 123.0, 122.42, 122.0, 120.3, 119.1, 85.5, 84.1, 84.0, 83.6, 78.3, 72.9, 55.0, 44.3, 40.3, 39.6, 38.4, 35.0, 31.5.

HRMS (ES+): calcd for $C_{102}H_{96}N_{19}O_8$ 1714.7684 [M+H]$^+$, found 1714.7640 [M+H]$^+$.

FT-IR (ATR): ν_{max} 2964, 2127, 2095, 1739, 1635, 1249, 1200 and 747 cm^{-1}.

^{1}H-NMR (400 MHz, CDCl$_3$) Compound 15.

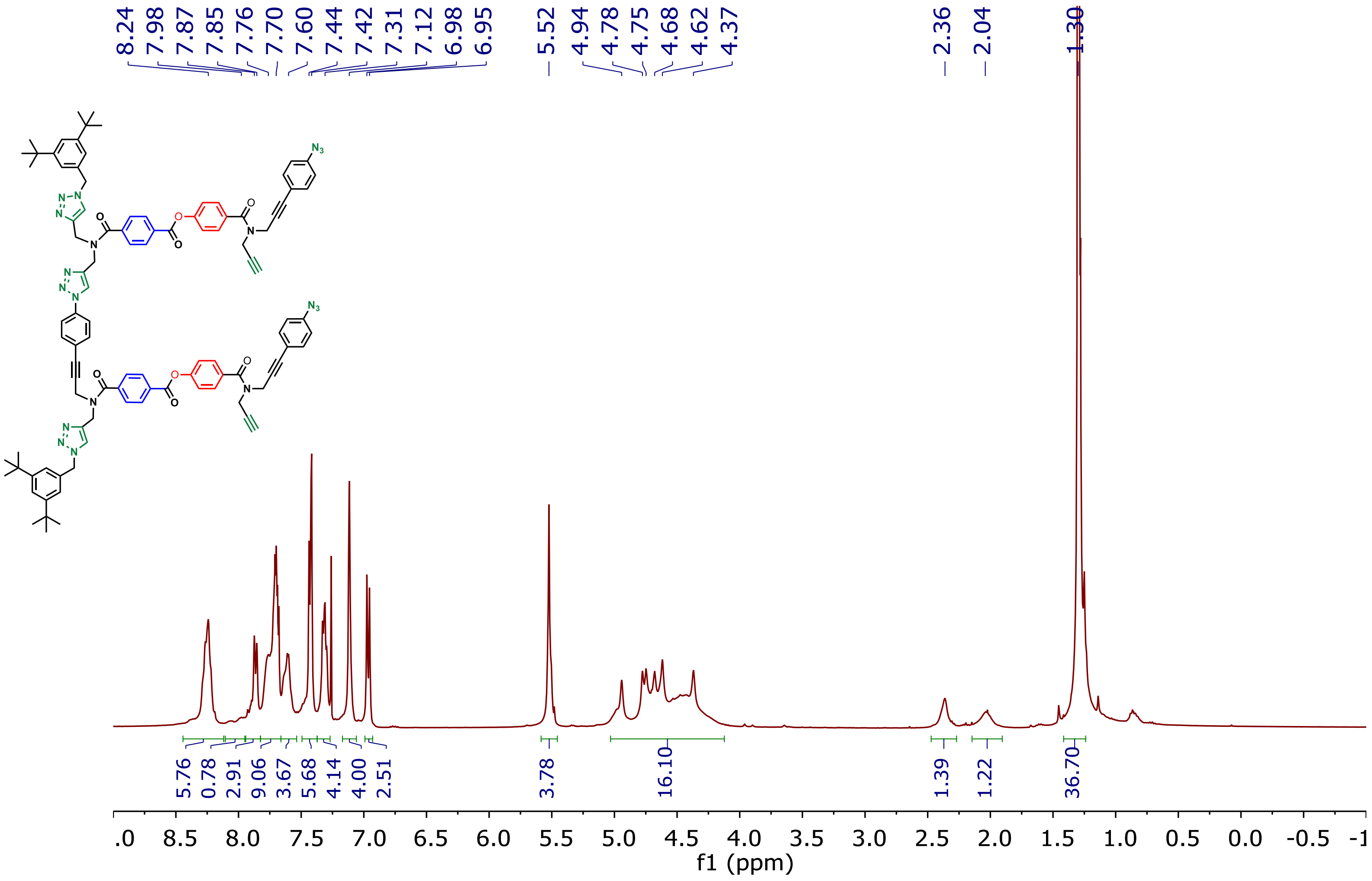

^{13}C-NMR (100.6 MHz, $CDCl_3$) Compound 15.

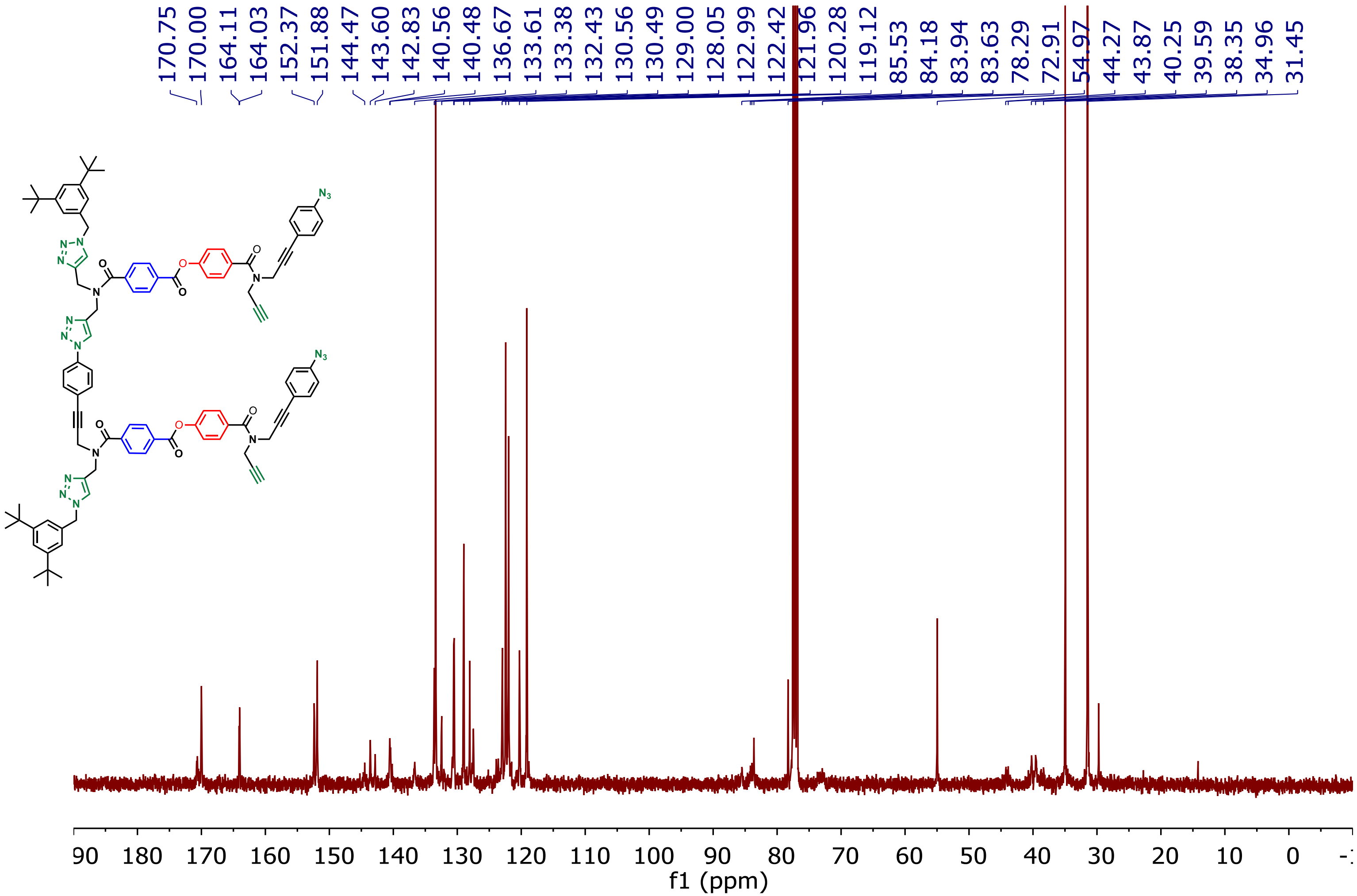

Compound S3.

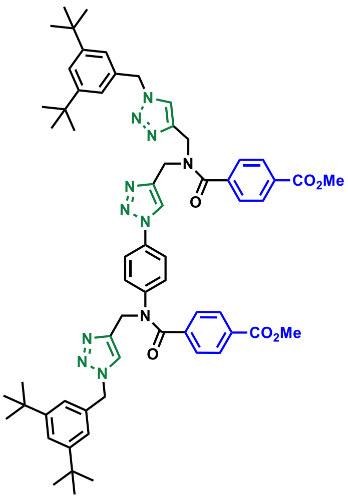

17 (0.035 g, 0.042 mmol), $Cu(CH_3CN)_4PF_6$ (0.002 g, 0.006 mmol) and TBTA (0.003 g, 0.006 mmol) were mixed in a round-bottom flask and, under N_2, THF (2 mL) was added. A solution of 1-(azidomethyl)-3,5-di-*tert*-butylbenzene[S6] (0.016 g, 0.063 mmol) in THF (1 mL) was added and the reaction was stirred overnight at room temperature. The solvent was evaporated to dryness and the crude was purified by flash column chromatography on silica gel (gradient from 5% to 85%of EtOAc in Pet. Ether) to afford **S3** (0.043 g, 94%) as a foam.

^{1}H NMR (400 MHz, CDCl$_3$): δ_H = 8.08 (m, 3H), 7.85 (d, 2H, *J* = 8.0 Hz), 7.74 (d, 2H, *J* = 8.0 Hz), 7.70 (s, 1H), 7.60 (t, 2H, *J* = 7.5 Hz), 7.68 and 7.53 (s, 1H, splitting is due to the existence of rotamers around the amide bond; only a singlet would be expected in the absence of rotamers), 7.42 (m, 1H), 7.40 (m, 1H), 7.32 (m, 4H), 7.10 (s, 2H), 7.08 and 7.07 (s, 2H, splitting is due to the existence of rotamers around the amide bond; only a singlet would be expected in the absence of rotamers), 5.51 (m, 4H), 5.14 (s, 2H), 4.72 and 4.70 (s, 2H, splitting is due to the existence of rotamers around the amide bond; only a singlet would be expected in the absence of rotamers), 4.61 and 4.55 (s, 2H, splitting is due to the existence of rotamers around the amide bond; only a singlet would be expected in the absence of rotamers), 3.91 (s, 3H), 3.85 (s, 3H), 1.30 (s, 18H), 1.28 (s, 18H).

^{13}C NMR (100.6 MHz, CDCl$_3$): δ_C = 169.3, 166.4, 166.2, 152.0, 151.9, 143.8, 139.6, 139.5, 133.8, 133.7, 131.61, 129.9, 129.4, 128.8, 127.8, 124.0, 122. 9, 122.5, 122.3, 121.2, 55.0, 54.9, 52.4, 46.3, 44.2, 35.0, 31.5. N-CH_2 peaks are not listed due to broadness caused by the existence of rotamers.

HRMS (ES+): calcd for $C_{63}H_{74}N_{11}O_6$ 1080.5824 $[M+H]^+$, found 1080.5872 $[M+H]^+$.

FT-IR (ATR): ν_{max} 2954, 2926, 2864, 1725, 1642, 1519, 1278, 1249, 1110 and 755 cm^{-1}.

^{1}H-NMR (400 MHz, $CDCl_3$) Compound S3.

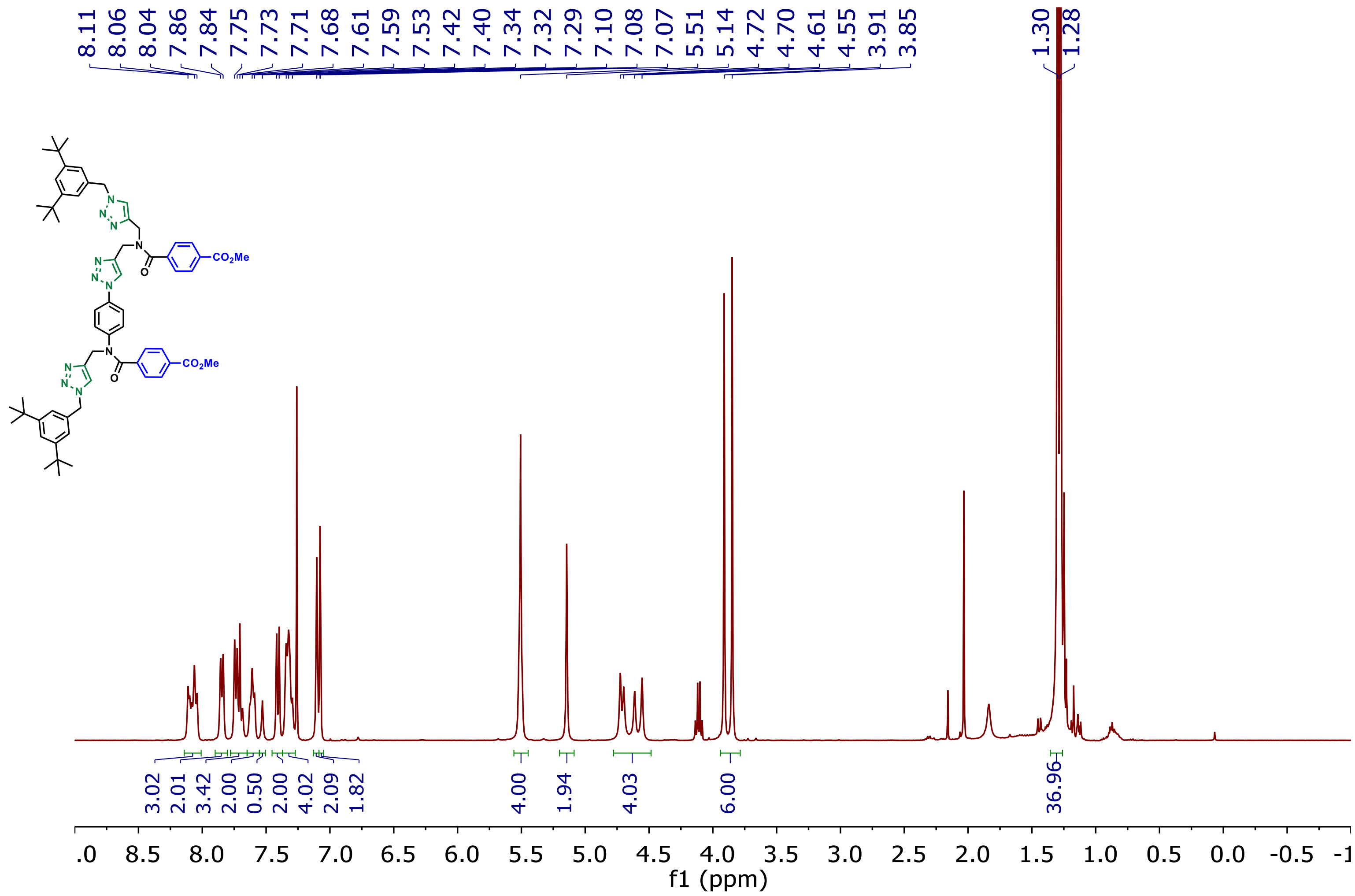

^{13}C-NMR (100.6 MHz, $CDCl_3$) Compound S3.

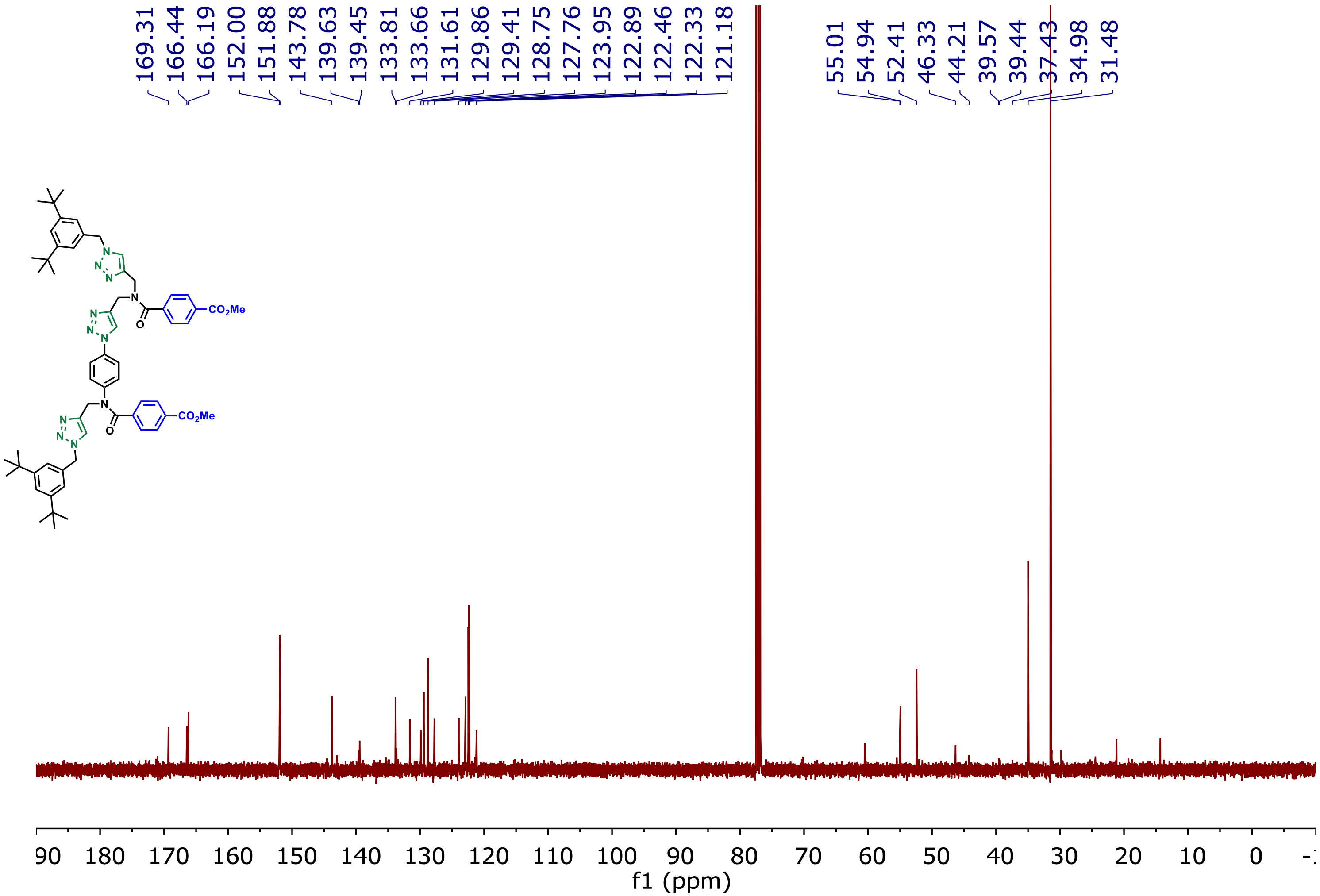

Compound 18.

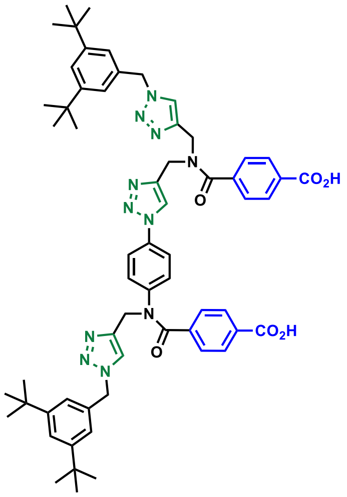

Compound **S1** (0.059 g, 0.054 mmol) was dissolved in THF/MeOH/H_2O 2.5:1:1 (2 mL) and LiOH (0.011 g, 0.273 mmol) was added. After 6 h of stirring at room temperature, the crude diluted with H_2O and acidified with 1 M HCl soln. to pH 2-3. The aqueous phase was extracted with EtOAc (3x) and the combined organic phase was washed with brine, dried over $MgSO_4$ and evaporated to dryness. The obtained yellow solid **18** (0.052 g, 91%) was used without further purification.

^{1}H NMR (500 MHz, DMSO-*d*$_6$): δ_H = 8.68 and 850 (bs, 1H, splitting is due to the existence of rotamers around the amide bond; only a singlet would be expected in the absence of rotamers), 8.23 (s, 1H), 8.19 (s, 1H), 7.91 (bs, 2H), 7.76 (d, 2H, *J* = 7.5 Hz), 7.70 (bs, 2H), 7.53 and 7.38 (bs, 2H, splitting is due to the existence of rotamers around the amide bond; only a singlet would be expected in the absence of rotamers), 7.32 (m, 6H), 7.13 (s, 2H), 7.06 (s, 2H), 5.56 (s, 2H), 5.53 (s, 2H), 5.16 (s, 2H), 4.74, 4.66 and 4.49 (s, 4H, splitting is due to the existence of rotamers around the amide bond; only two singlets would be expected in the absence of rotamers), 1.24 (s, 18H), 1.19 (s, 18H).

^{13}C NMR (126 MHz, DMSO-*d*$_6$): δ_C = 170.4, 169.2, 150.8, 150.7, 144.2, 143.1, 142.8, 137.9, 135.4, 135.3, 134.4, 129.3, 129.2, 129.0, 128.7, 127.8, 125.9, 124.0, 123.9, 121.9, 121.7, 121.6, 121.5, 120.3, 53.3, 53.2, 44.9, 43.8, 39.5, 34.5, 34.4, 31.2, 31.1.

HRMS (ES+): calcd for $C_{61}H_{70}N_{11}O_6$ 1052.5511 $[M+H]^+$, found 1052.5493 $[M+H]^+$.

FT-IR (ATR): ν_{max} 2956, 2924, 2863, 1713, 1636, 1600, 1519, 1407, 1249, 1049 and 1025 cm^{-1}.

^{1}H NMR (500 MHz, DMSO-d_6) Compound 18.

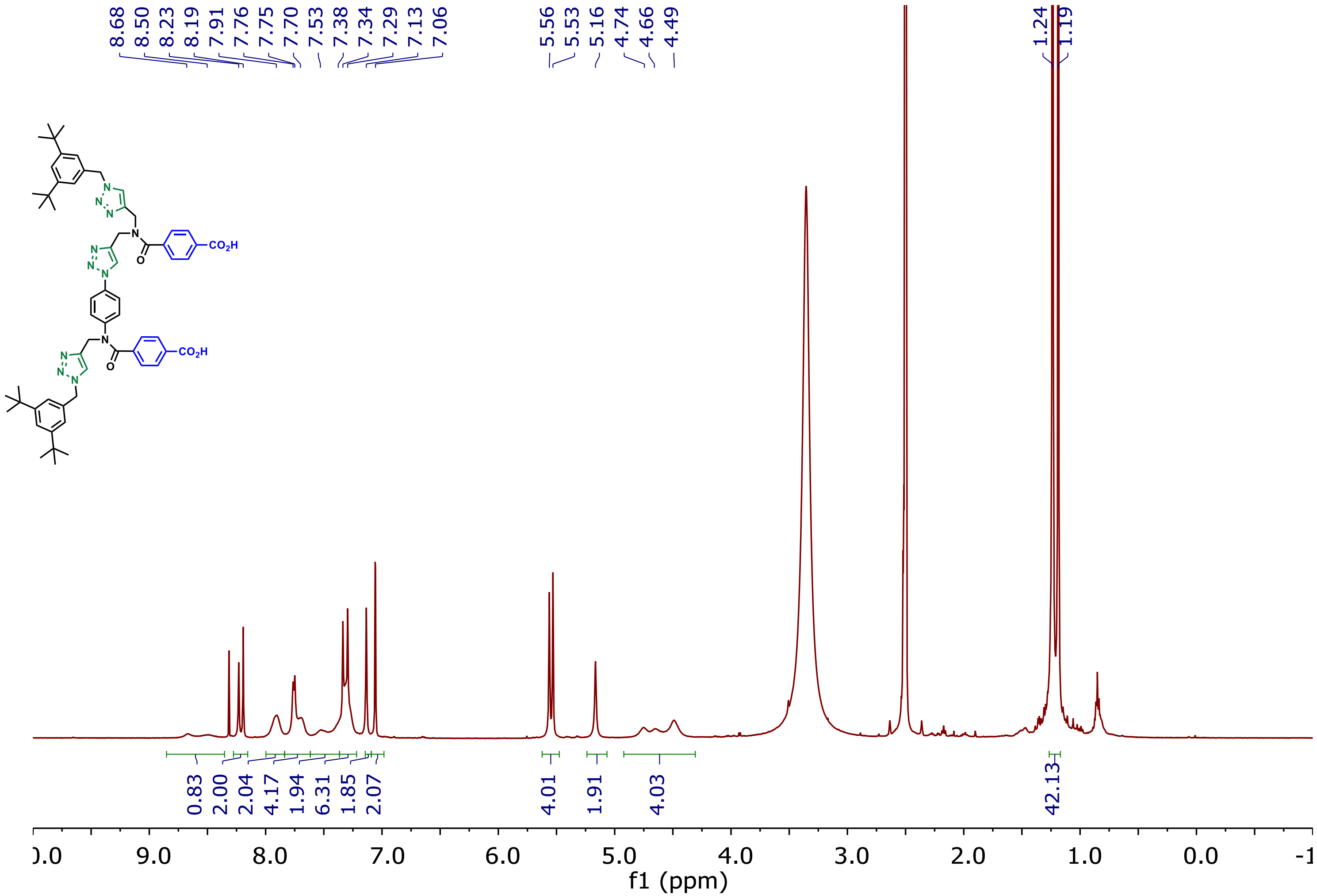

^{13}C NMR (126 MHz, DMSO-d_6) Compound 18.

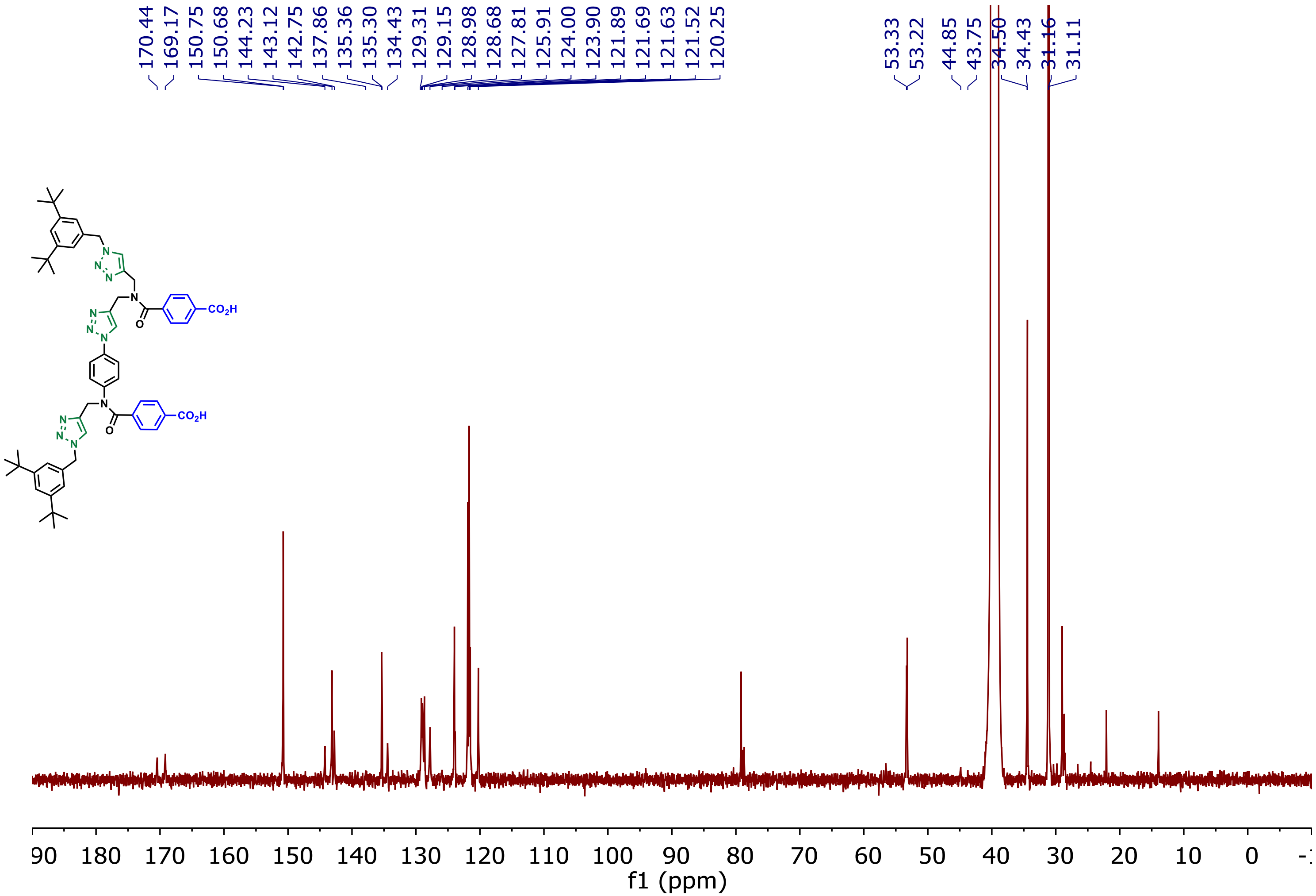

Pre-ZIP 20.

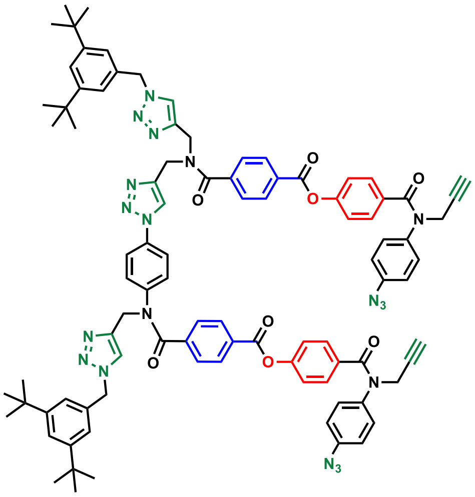

A solution of **18** (0.052 g, 0.050 mmol), **19**[S2] (0.044 g, 0.149 mmol), EDC (0.028 g, 0.149 mmol) and DMAP (5 mg, 0.045 mmol) in dry CH_2Cl_2 (5 mL) under N_2 atmosphere was stirred at room temperature for 16 h. The reaction was diluted with EtOAc (5 mL) and washed with 0.1 N HCl soln. (3x), H_2O (1x) and brine. The organic phase was dried with $MgSO_4$ and concentrated *in vacuo*. The crude material was purified by flash column chromatography on silica gel (gradient from 0% to 4% of MeOH in CH_2Cl_2) to afford **20** (0.025 g, 31 %) as a light-yellow foam. This molecule is not stable upon long-term storage, due to azide-alkyne cycloaddition reaction occurring even in the absence of copper (I) catalysts, yielding a mixture of regioisomers. NMR spectra show the existence of minor impurities corresponding to these adducts.

^{1}H NMR (400 MHz, CDCl$_3$): δ_H = 8.17 (m, 3H), 7.95 (m, 2H), 7.82 (m, 2H), 7.71 (s, 1H), 7.69 and 7.56 (s, 1H, splitting is due to the existence of rotamers around the amide bond; only a singlet would be expected in the absence of rotamers), 7.63 (m, 2H), 7.40 (m, 10H), 7.15 (d, 2H, *J* = 8.5 Hz), 7.10 (m, 8H), 7.03 (d, 2H, *J* = 8.5 Hz), 6.95 (d, 2H, *J* = 8.5 Hz), 6.91 (d, 2H, *J* = 8.5 Hz), 5.52 (m, 4H), 5.15 (s, 2H), 4.72-4.57 (m, 8H, some splitting is due to the existence of rotamers around the amide bond), 2.26 (m, 2H), 1.30 (s, 18H), 1.28 (s, 18H).

^{13}C NMR (100.6 MHz, CDCl$_3$): δ_C = 170.8, 170.7, 169.2, 169.1, 164.0, 163.7, 152.1, 152.0, 151.9, 144.5, 143.7, 142.9, 140.5, 140.5, 140.4, 139.5, 139.5, 139.4, 139.4, 135.4, 135.3, 133.8, 133.7, 132.7, 130.6, 130.6, 130.5, 130.0, 129.3, 129.0, 128. 9, 128.1, 124.0, 123.1, 123.1, 123.0, 122.5, 122.4, 121.3, 121.2, 120.1, 120.0, 78.8, 72.8, 55.1, 55.0, 46.4, 44.2, 43.8, 40.1, 39.6, 39.4, 35.0, 31.5.

HRMS (ES+): calcd for $C_{93}H_{90}N_{19}O_8$ 1601.7253 $[M+H]^+$, found 1601.7174 $[M+H]^+$.

FT-IR (ATR): ν_{max} 2960, 2930, 2865, 2123, 2095, 1642, 1505, 1260, 1201, 1067 and 750 cm^{-1}.

^{1}H-NMR (400 MHz, $CDCl_3$) Compound 20.

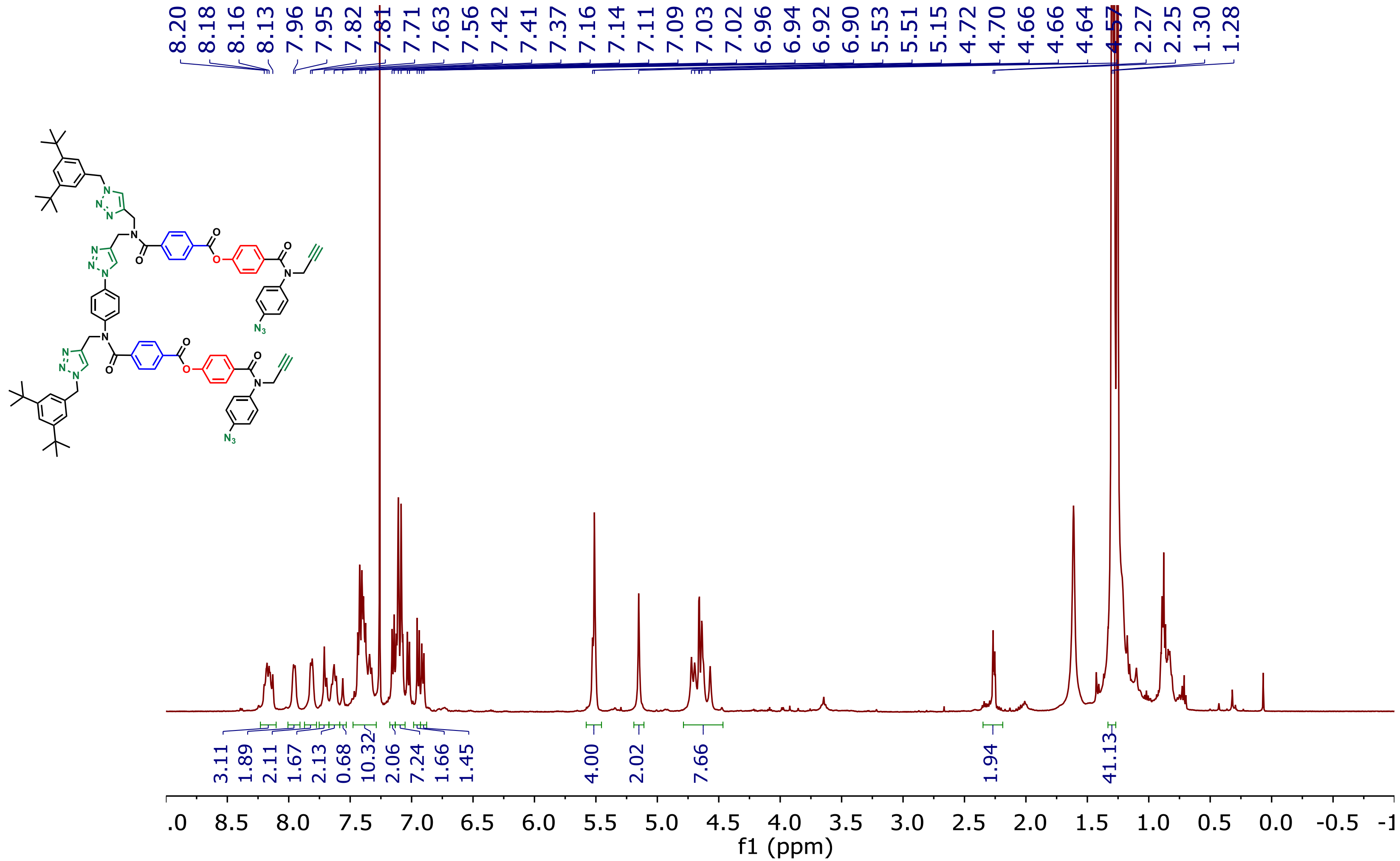

^{13}C-NMR (100.6 MHz, $CDCl_3$) Compound 20.

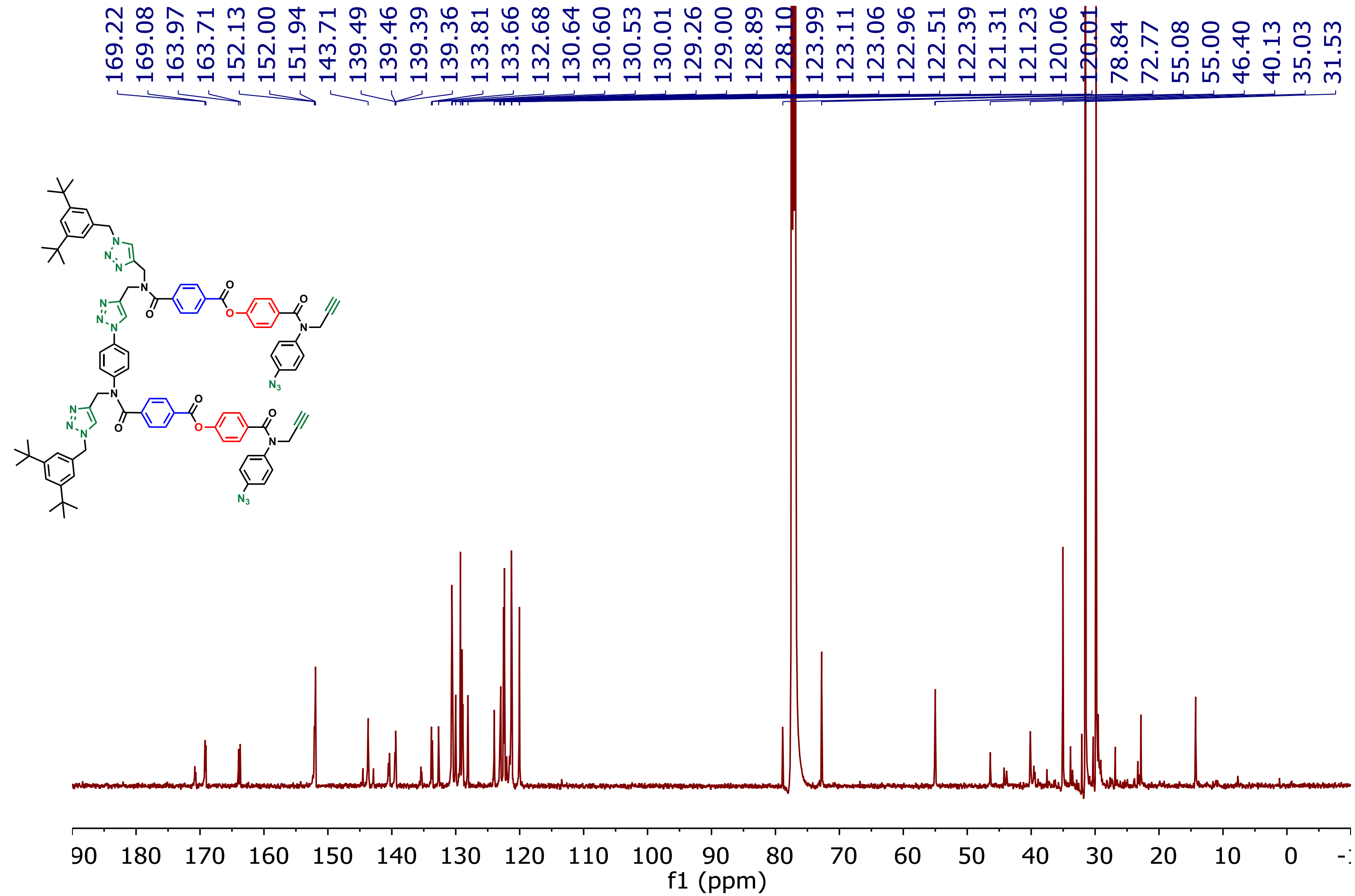

Cyclic duplex 22.

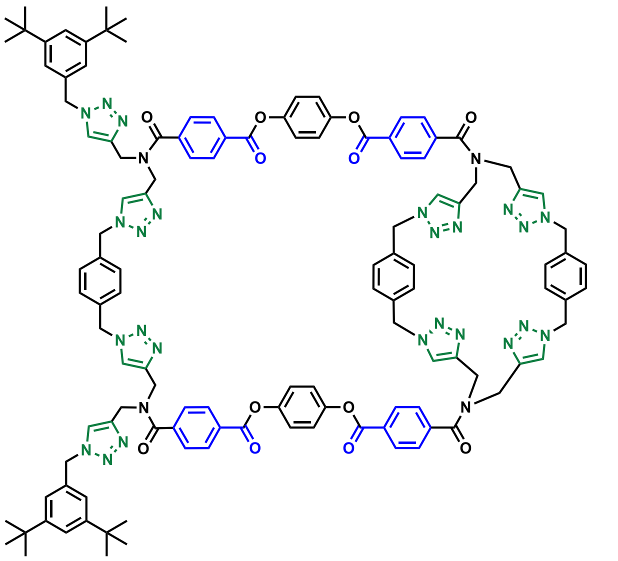

Compound **8** (25 mg, 0.012 mmol) was dissolved, under N_2, in dry THF (50 mL). $Cu(CH_3CN)_4PF_6$ (4 mg, 0.012 mmol) and TBTA (6 mg, 0.012 mmol) were added and the reaction was stirred overnight at room temperature. The reaction was diluted with EtOAc and washed with 0.02 M NaEDTA neutral aq. soln.(2x) and brine (1x). The organic layer was dried over $MgSO_4$ and the solvent evaporated to dryness. The crude was purified by flash column chromatography on silica gel (gradient from 0% to 15% of MeOH in CH_2Cl_2) to afford compound **22** (14 mg, 57%) as a white solid.

Melting point: 201-202 °C.

^{1}H NMR (500 MHz, CD_3CN): δ_H = 8.13 (bs, 8H), 7.82-7.54 (m, 18H), 7.43 (s, 2H), 7.32-7.23 (m, 18H), 7.11 (s, 4H), 5.48 (m, 16 H), 4.63-4.43 (bs, 16H), 1.27 (s, 36H).

^{13}C NMR (125.7 MHz, CD_3CN): δ_C = 171.2, 171.1, 165.5, 165.5, 152.6, 149.6, 145.0, 144.7, 144.1, 142.3, 137.1, 136.2, 136.1, 131.3, 131.1, 129.8, 129.5, 129.4, 128.3, 124.4, 124.2, 123.9, 123.4, 123.4, 55.0, 54.0, 53.9, 45.0, 44.9, 40.8, 40.7, 35.6, 31.6.

HRMS (ES+): calcd for $C_{122}H_{119}N_{28}O_{12}$ 2167.9562 $[M+H]^+$, found 2167.9565 $[M+H]^+$.

FT-IR (ATR): ν_{max} 3017, 2961, 1736, 1633, 1262, 1217, 1174 and 751 cm^{-1}.

^{1}H-NMR (500 MHz, CD_3CN) Compound 22.

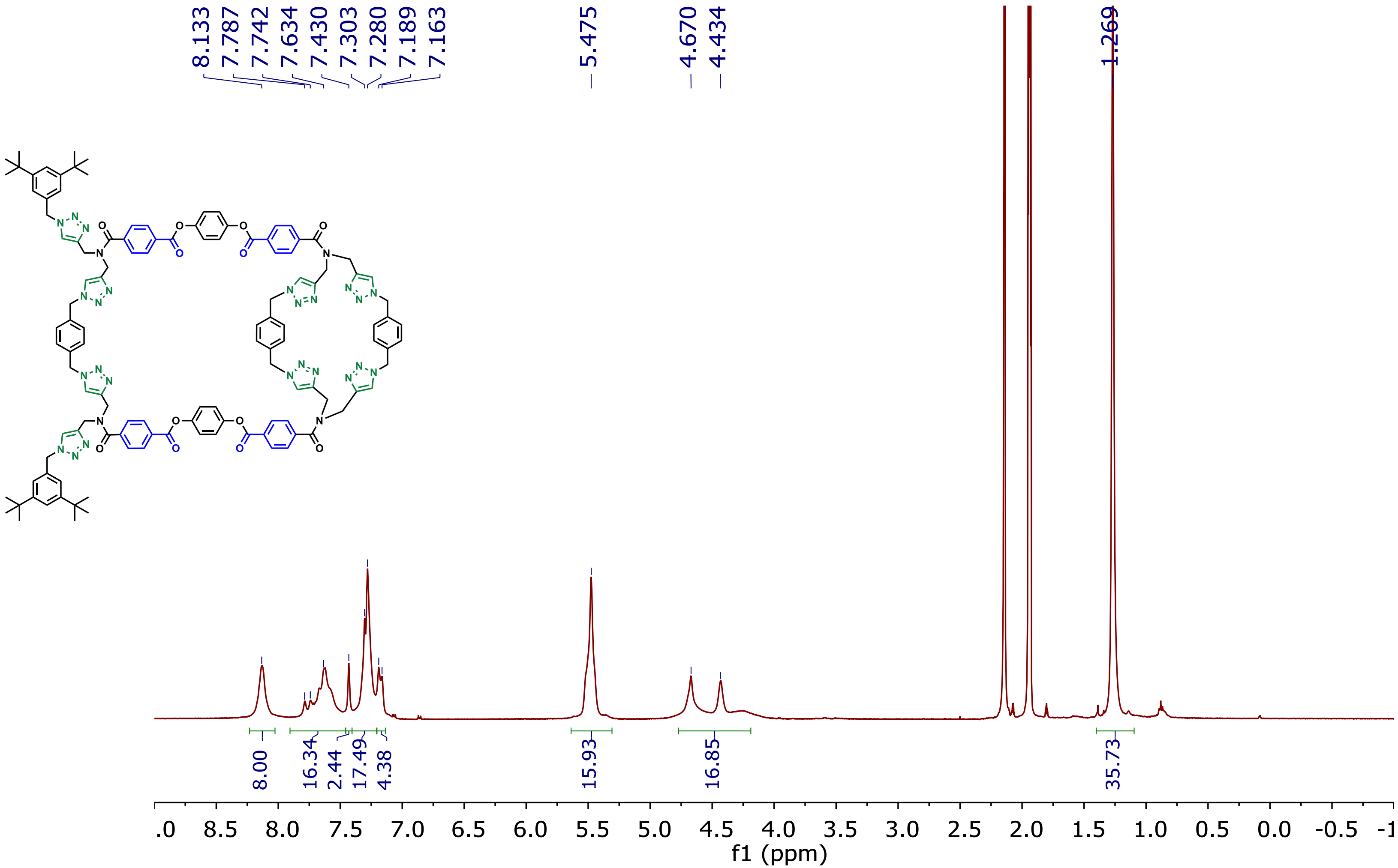

^{13}C-NMR (125.7 MHz, CD_3CN) Compound 22.

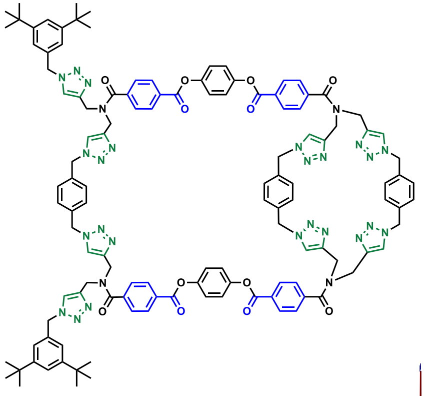

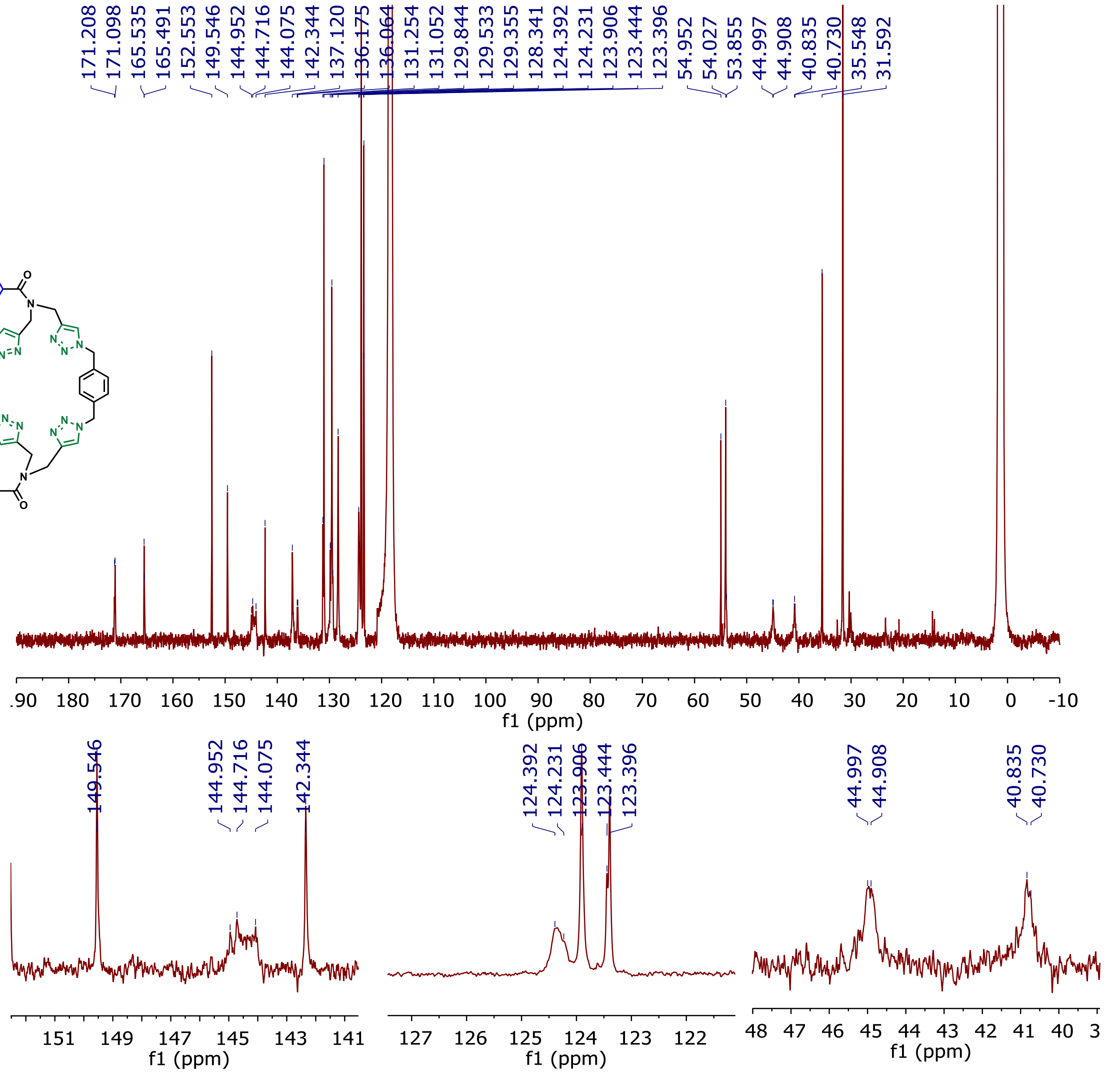

COSY (CD_3CN) Compound 22.

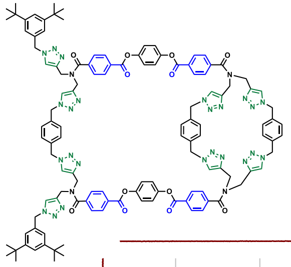

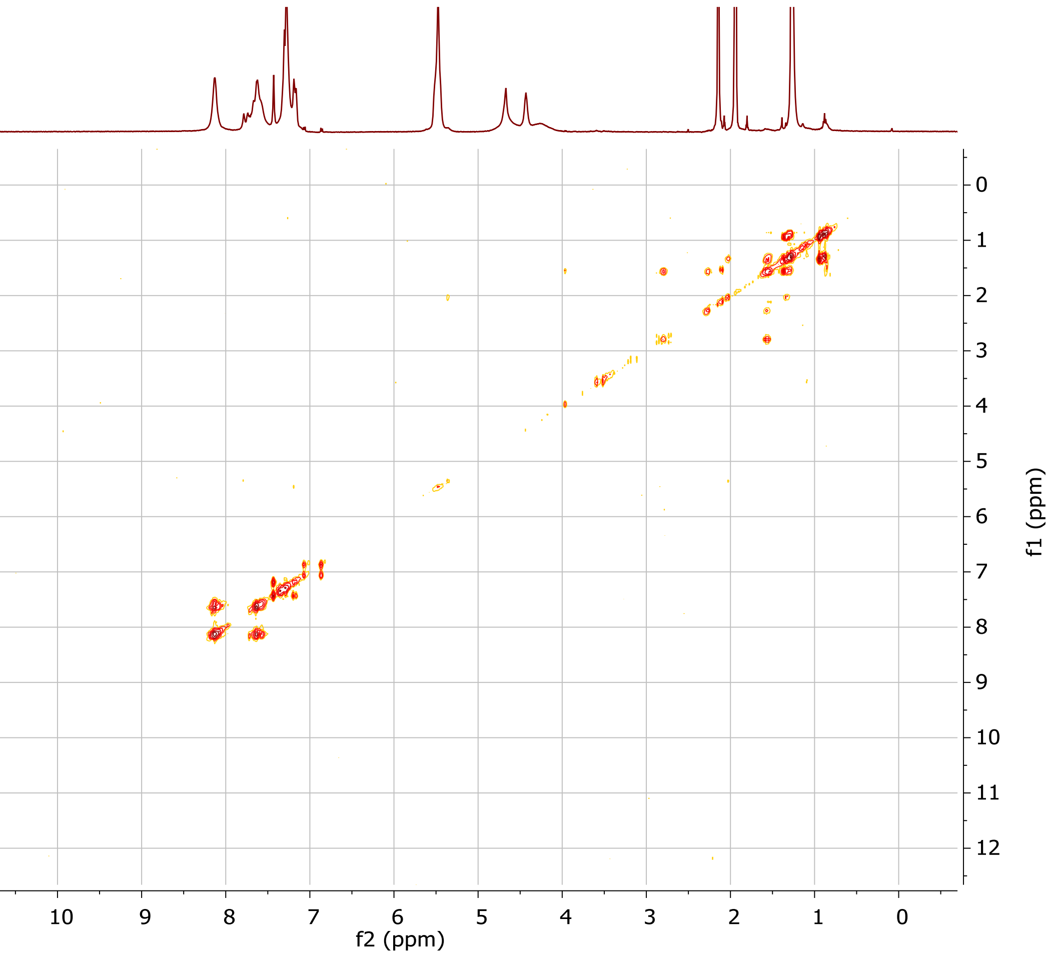

HSQC (CD_3CN) Compound 22.

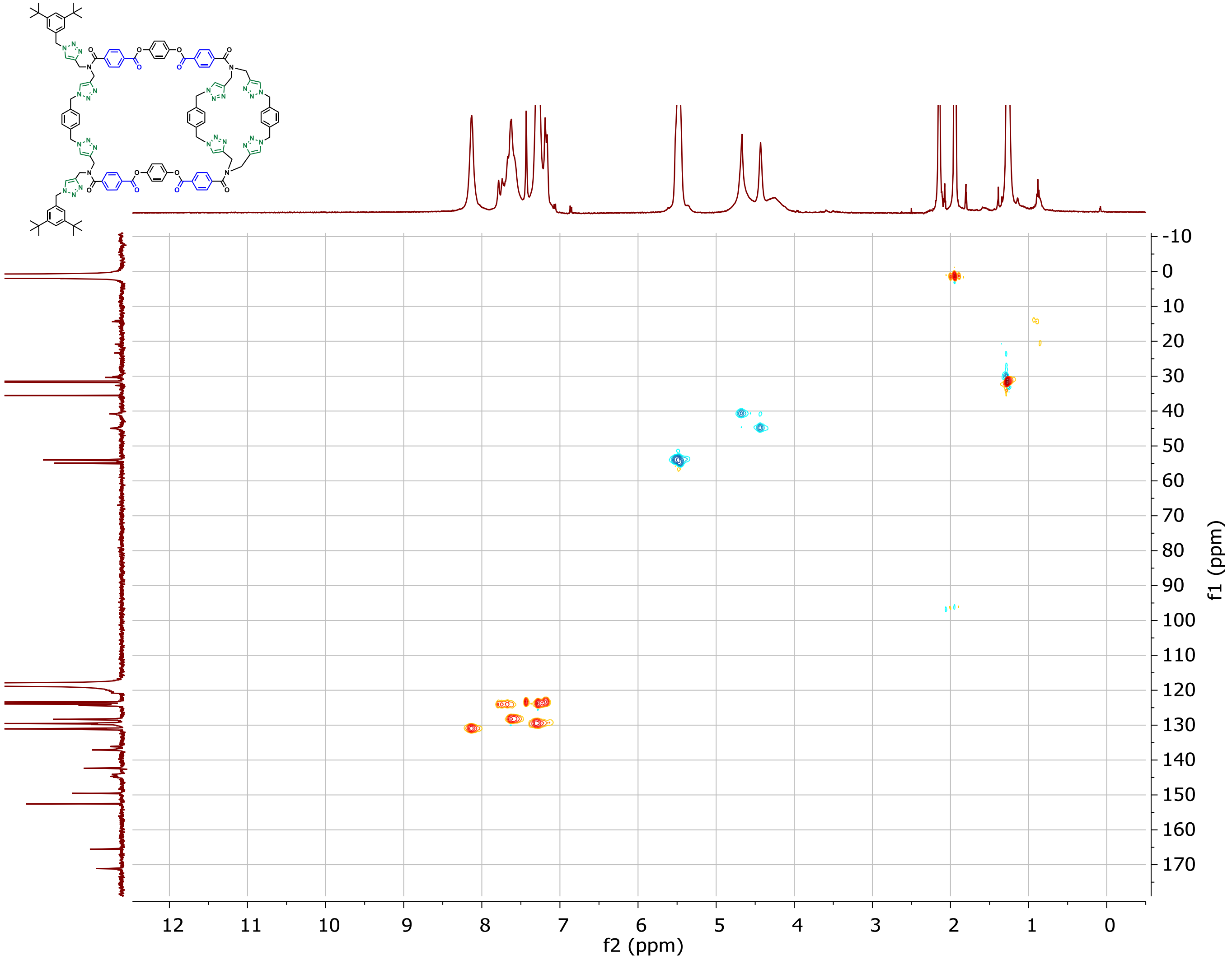

HMBC (CD_3CN) Compound 22.

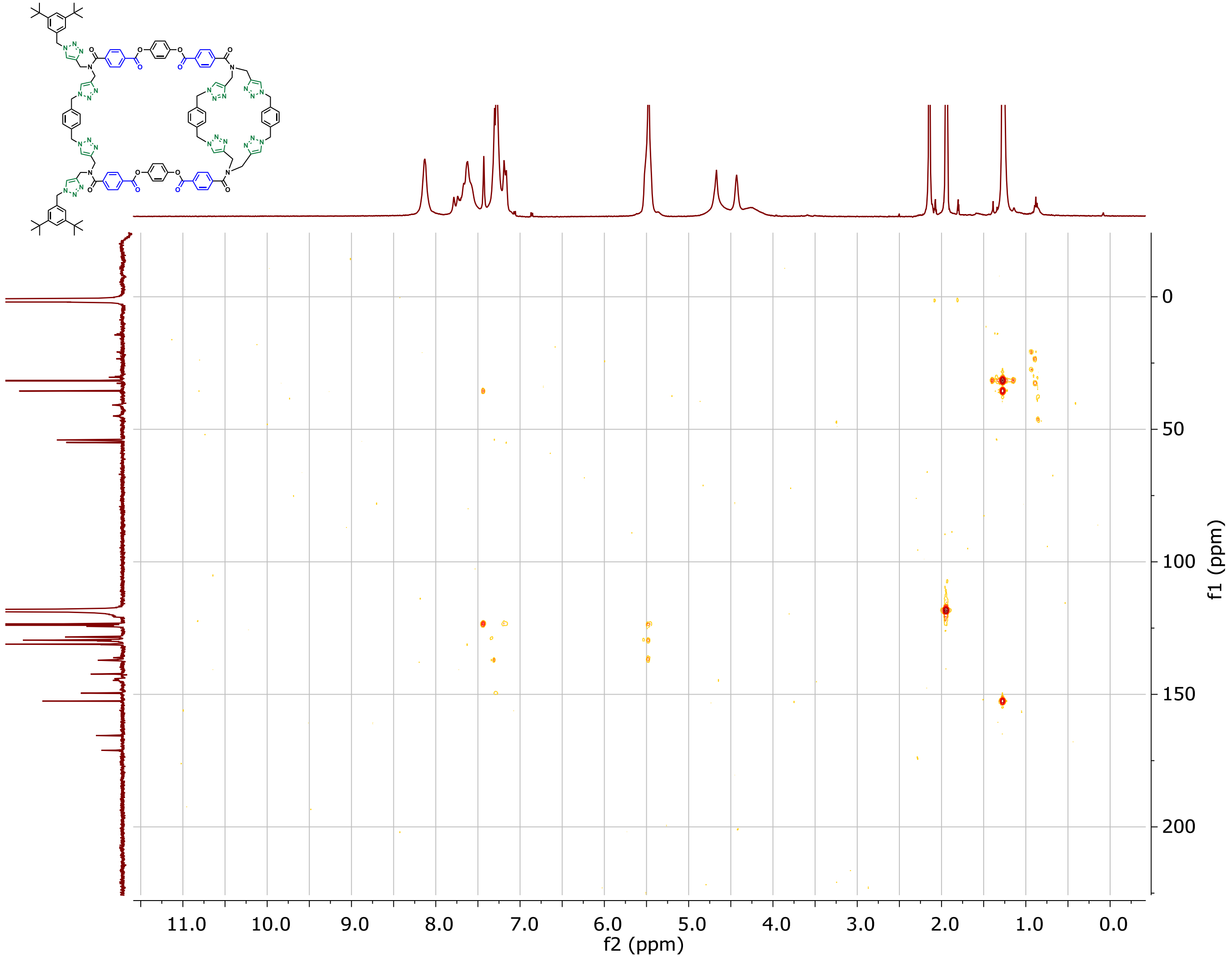

Cyclic duplex 25.

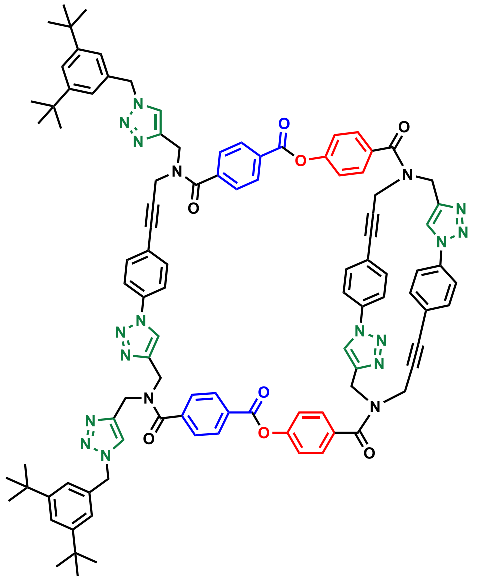

Pre-ZIP **15** (10 mg, 5.8·10^{-3} mmol) was dissolved, under N_2, in dry THF (120 mL). $Cu(CH_3CN)_4PF_6$ (4 mg, 0.012 mmol) and TBTA (6 mg, 0.012 mmol) were added and the reaction was stirred overnight at room temperature. The solvent was evaporated and the reaction mixture was purified by flash column chromatography on silica gel (gradient from 0% to 5% of MeOH in CH_2Cl_2). The fractions corresponding to the product were combined, dried and filtered through a pad of silica using EtOAc as solvent, to remove traces of copper. Cyclic duplex **25** (7 mg, 70%) was afforded as a white solid, containing TBTA as a minor impurity.

^{1}H NMR (500 MHz, DMSO-*d*$_6$): δ_H = 8.84 (m, 3H), 8.29 (s, 1H), 8.26 (s, 1H), 8.24 (m, 4H), 7.91 (m, 2H), 7.85 (m, 5H), 7.74 (m, 3H), 7.60 (d, 2H, *J* = 8.0 Hz), 7.50 (m, 8H), 7.35 (m, 2H), 7.16 (s, 2H), 7.12 (m, 6H), 5.59 (s, 2H), 5.58 (s, 2H), 5.25-4.19 (m, 16H), 1.25 (s, 18H), 1.23 (s, 18H).

^{13}C NMR (125.7 MHz, DMSO-*d*$_6$): δ_C = 169.7, 169.6, 169.4, 169.4, 169.2, 169.2, 163.7, 163.7, 151.5, 151.4, 150.7, 143.7, 143.0, 143.0, 141.0, 140.5, 136.2, 135.9, 135.8, 133.0, 132.2, 132.0, 130.2, 130.0, 129.6, 128.7, 128.0, 127.7, 127.1, 124.2, 124.1, 124.0, 122.0, 121.9, 121.7, 120.0, 119.1, 87.9, 87.9, 83.5, 53.3, 47.1, 43.7, 40.8, 40.1, 39.6, 39.5, 34.5, 34.5, 31.2, 31.1.

HRMS (ES+): calcd for $C_{102}H_{95}N_{19}O_8Na$ 1736.7509 $[M+Na]^+$, found 1736.7529 $[M+Na]^+$.

FT-IR (ATR): ν_{max} 2956, 2925, 2856, 1740, 1639, 15118, 1455, 1260, 1204 and 841 cm^{-1}.

^{1}H-NMR (500 MHz, DMSO-d_6) Compound 25.

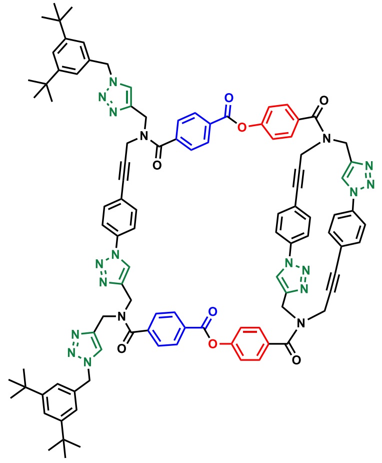

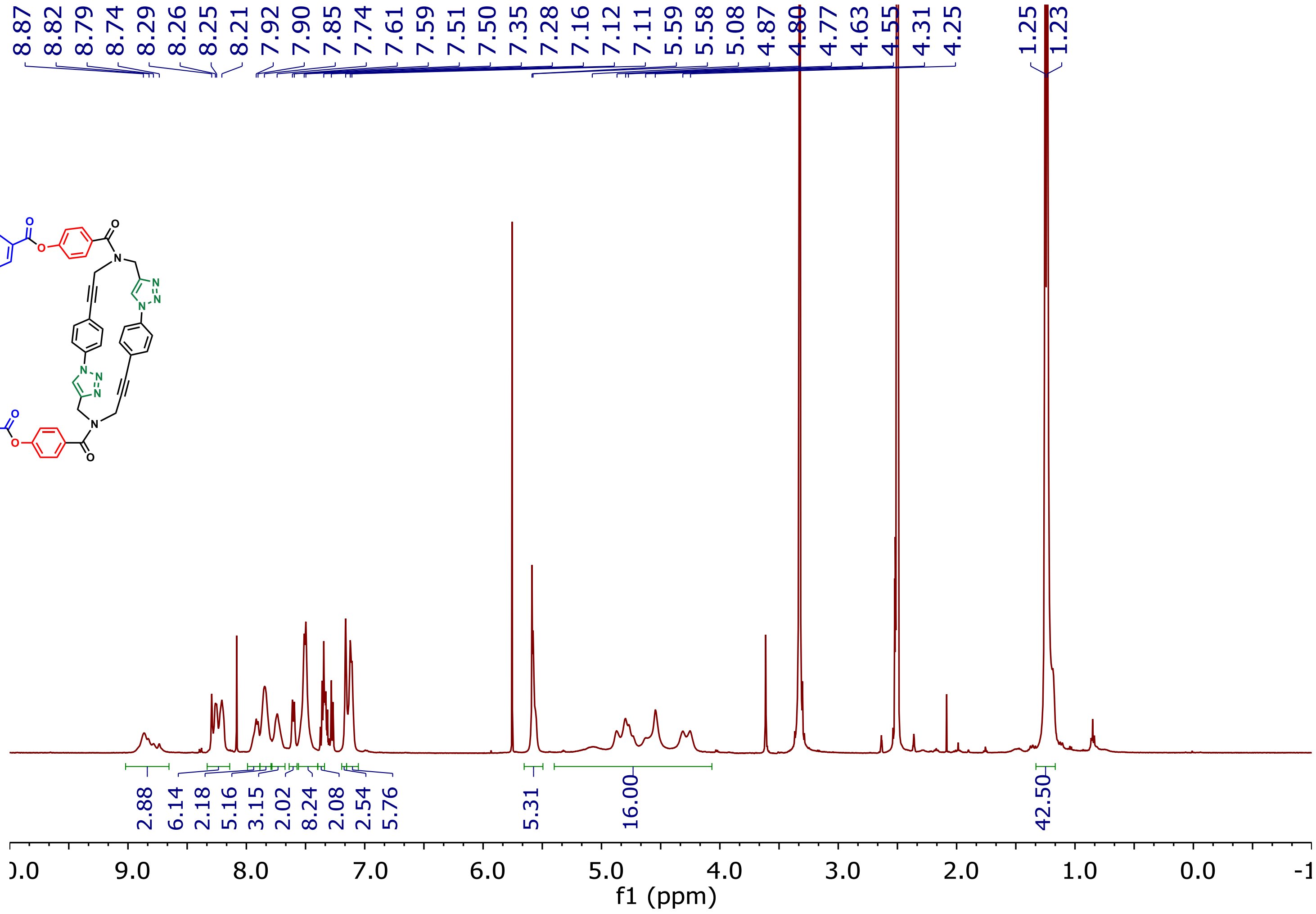

^{13}C NMR (125.7 MHz, DMSO-d_6) Compound 25.

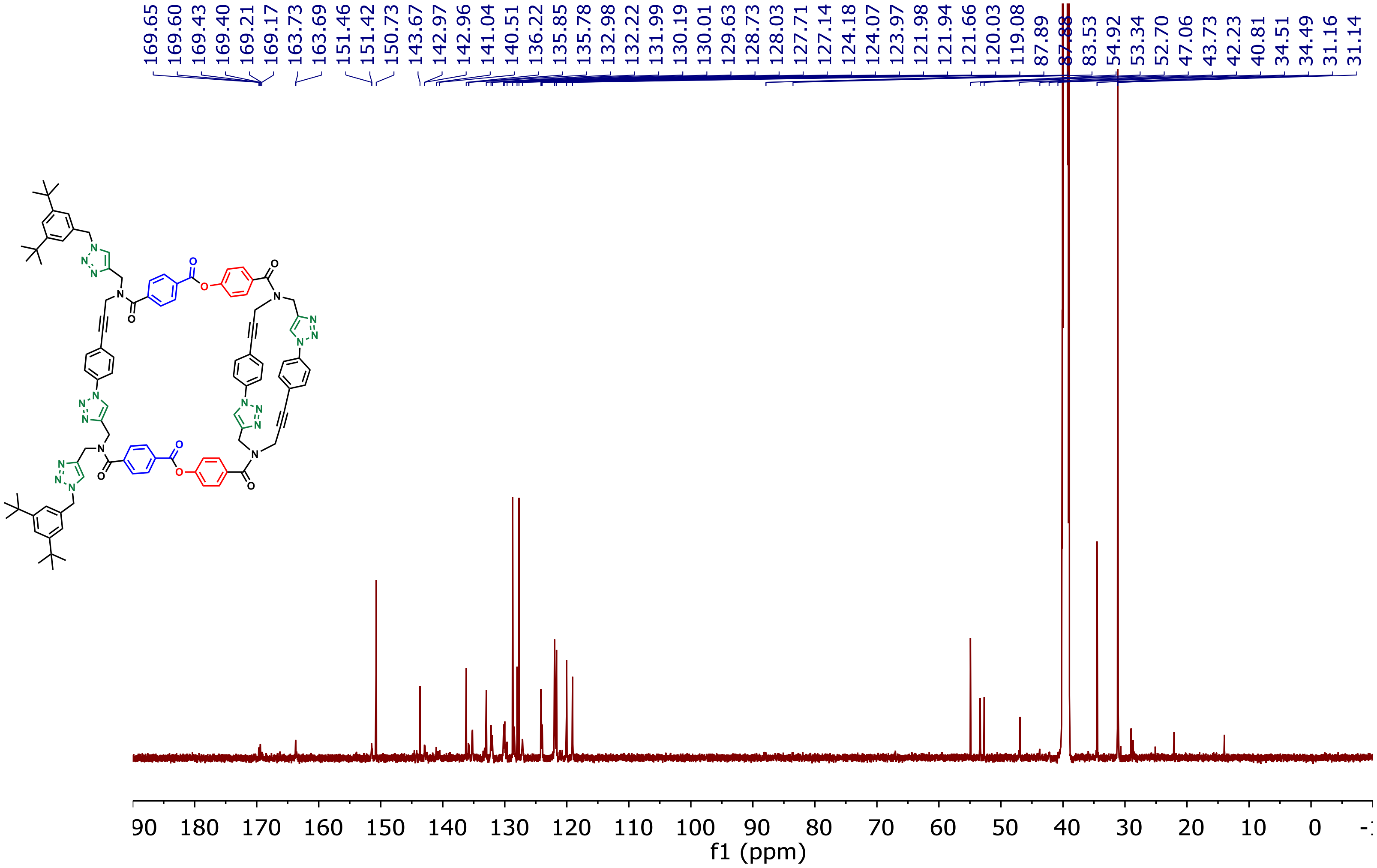

COSY (DMSO-d_6) Compound 25.

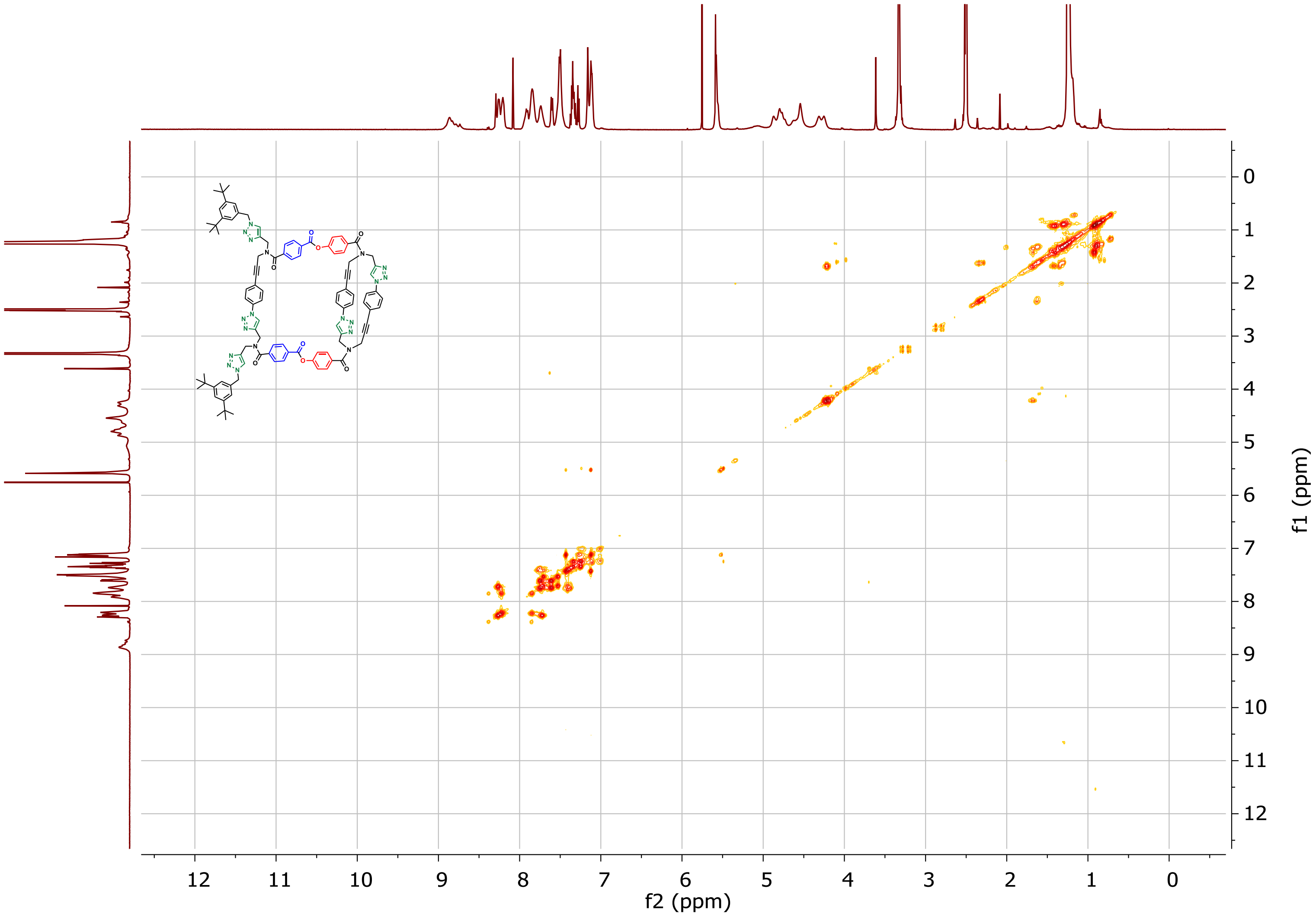

HSQC (DMSO-d_6) Compound 25.

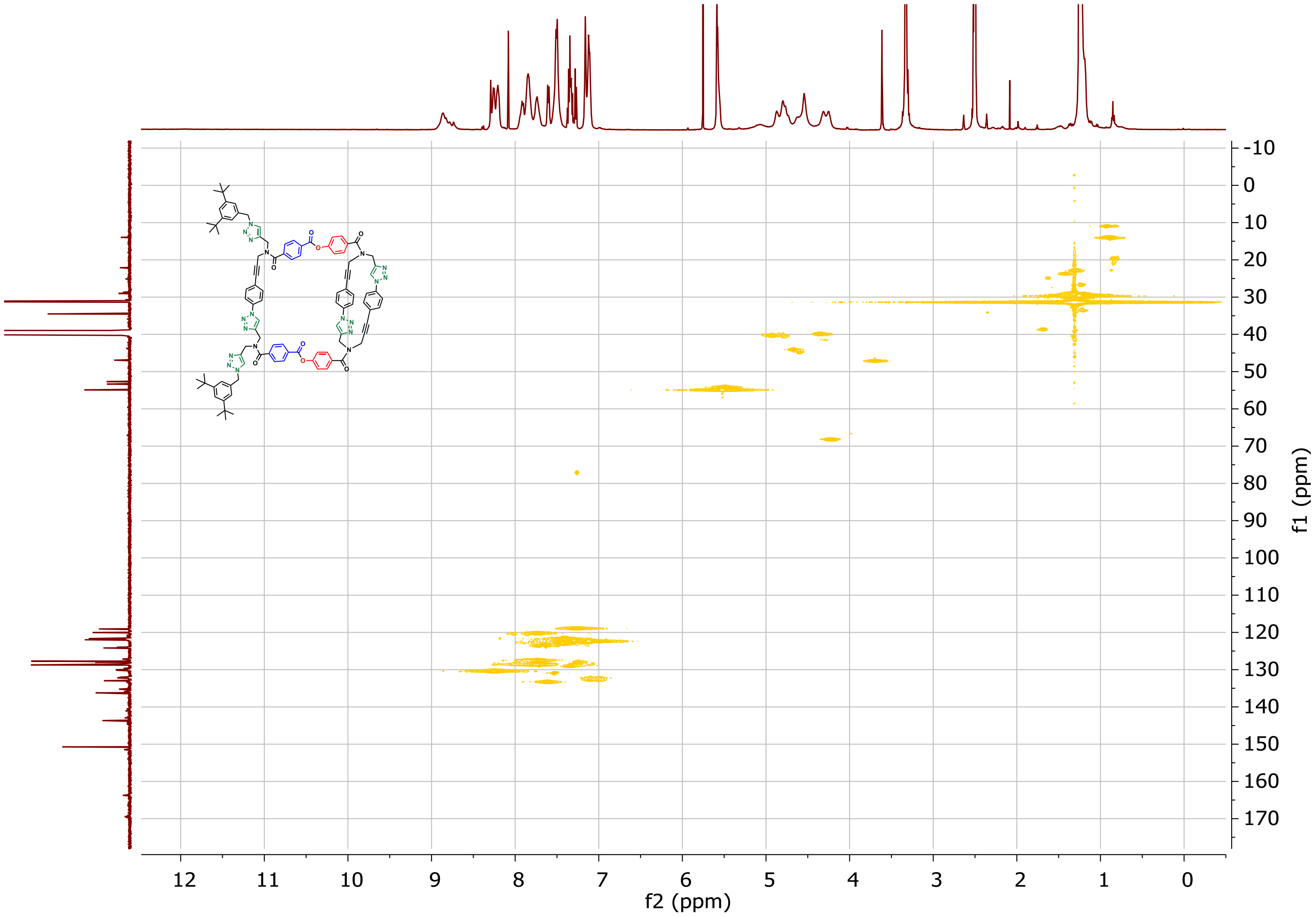

HMBC (DMSO-d_6) Compound 25.

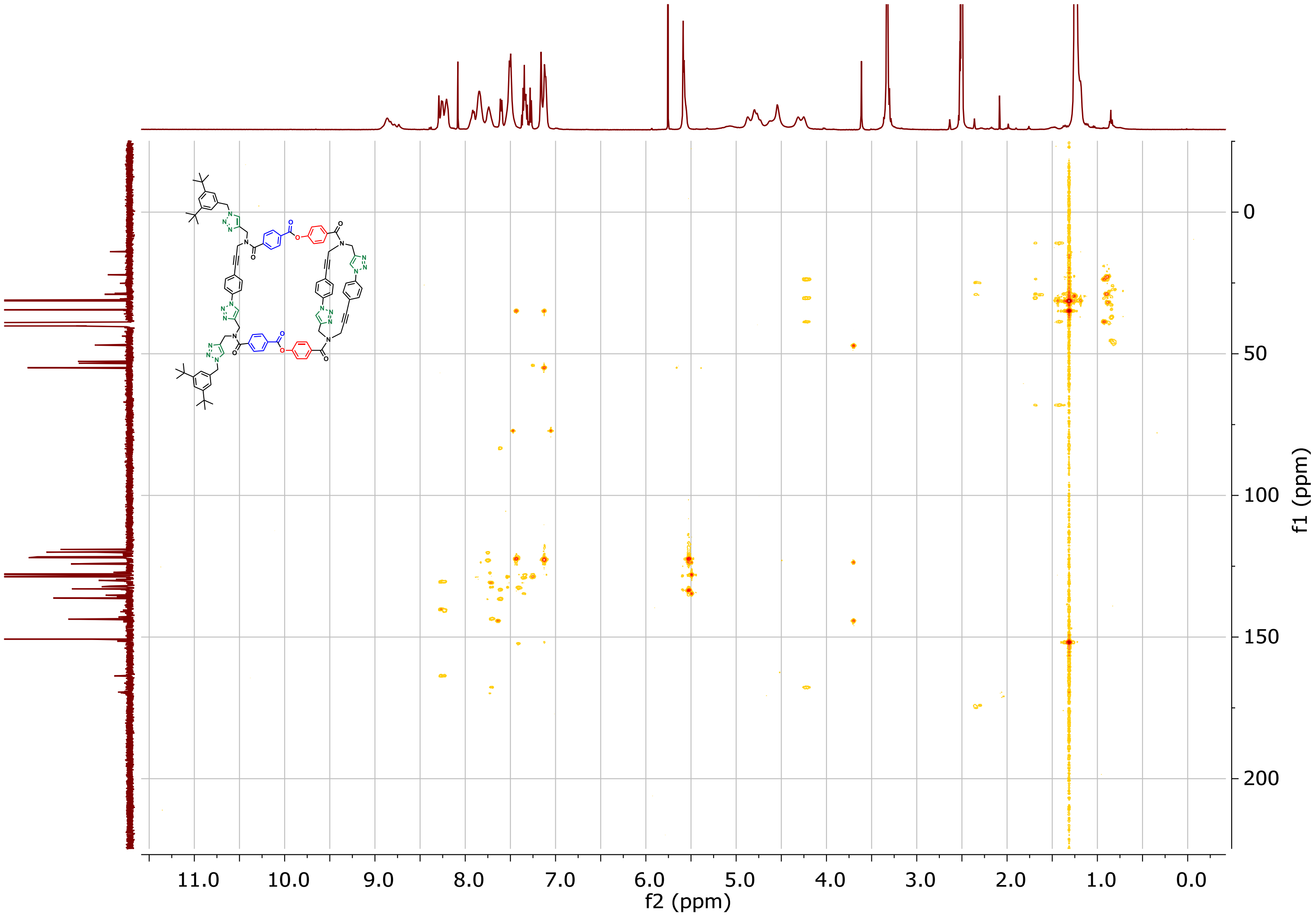

Synthesis of trimer pre-ZIP S7 (backbone B)

Compound S4 was synthesized in moderate yield by palladium-catalysed coupling of **3** and 4-azido-1-iodobenzene (Scheme S1). The CuAAC reaction of two equivalents of S4 with methyl 4-(di(prop-2-yn-1-yl)carbamoyl)benzoate[S3] yielded 3-mer **S5** in very good yield. Hydrolysis of the methyl ester, followed by cleavage of the alkyne protecting group and capping with 1-(azidomethyl)-3,5-di-*tert*-butylbenzene afforded 3-mer template **S6**. The coupling of **S6** with three equivalents of **11** gave pre-ZIP **S7** in good yield..

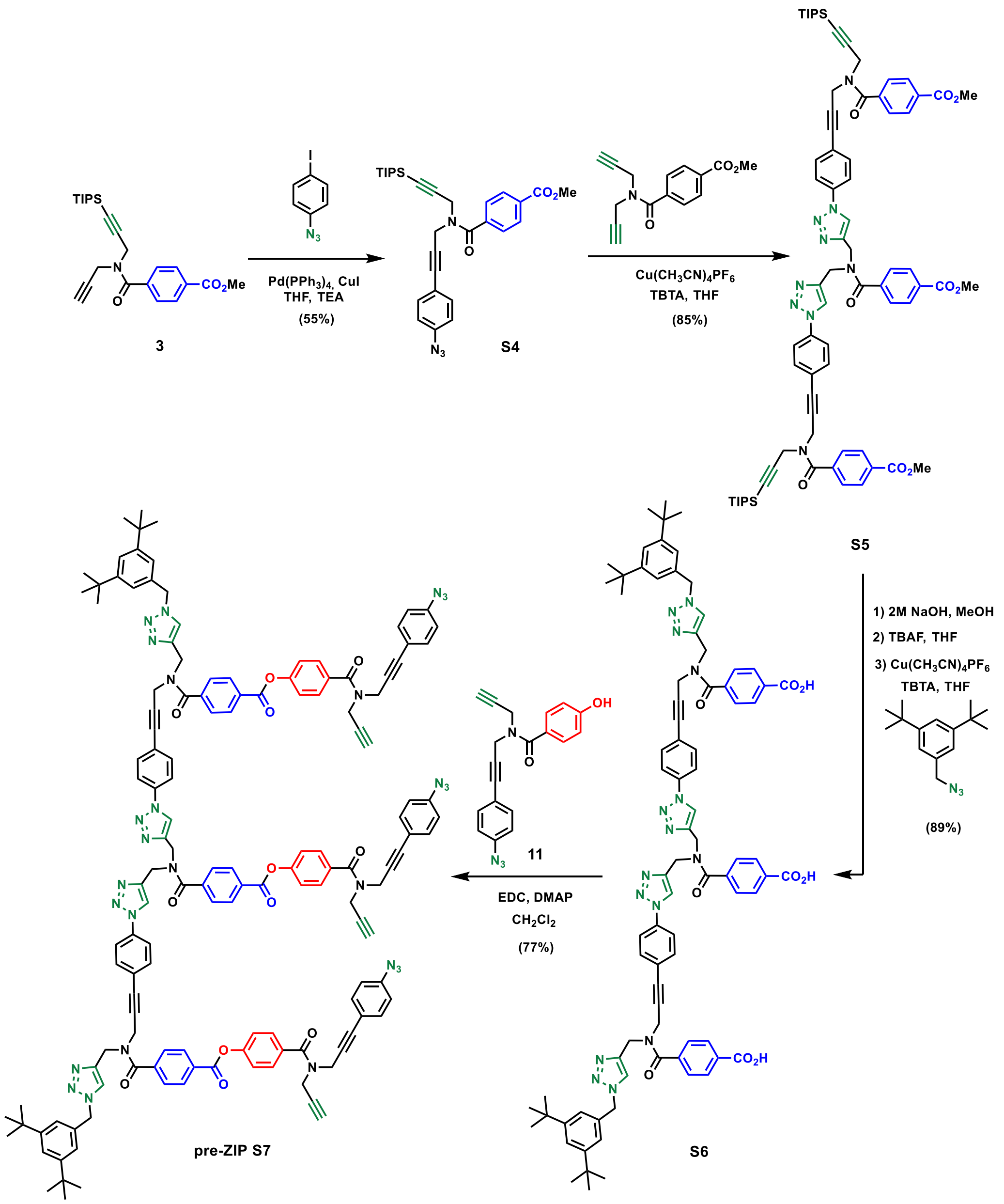

Scheme S1

Compound S4.

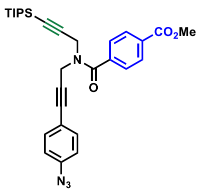

1-Azido-4-iodobenzene (0.278 g, 1.14 mmol), $Pd(PPh_3)_4$ (0.012 g, 0.001 mmol) and CuI (0.002 g, 0.001 mmol) were mixed under N_2 and THF/TEA 1:1 (5 mL) was added. Then, **3** (0.425 g, 1.03 mmol) dissolved in THF (1 mL) was added. After stirring at room temperature for 2 h, the reaction was quenched with 0.1 N HCl soln. and extracted with EtOAc (2x). The combined organic phase was washed with 0.02 M EDTA soln. and brine, dried over $MgSO_4$ and the solvent evaporated. The crude material was purified by flash column chromatography on silica gel (gradient from 0% to 15% of EtOAc in Pet. Ether) to afford **S4** (0.300 g, 55%) as a clear oil, recovering starting material **1** (0.085 g, 20%).

^{1}H NMR (400 MHz, CDCl$_3$): δ_H = 8.10 (d, 2H, *J* = 8.0 Hz), 7.66 (d, 2H, *J* = 8.0 Hz), 7.43 (d, 2H, *J* = 8.0 H), 6.97 (d, 2H, *J* = 8.0 Hz), 4.72 and 4.60 (bs, 2H, splitting is due to the existence of rotamers around the amide bond; only a singlet would be expected in the absence of rotamers), 4.38 and 4.19 (bs, 2H, splitting is due to the existence of rotamers around the amide bond; only a singlet would be expected in the absence of rotamers), 3.94 (s, 3H), 1.09 (s, 21H).

^{13}C NMR (100.6 MHz, CDCl$_3$): δ_C = 169.9, 166.4, 161.0, 133.5, 131.9, 130.0, 127.3, 125.1, 119.2, 101.4, 87.3, 83.7, 79.3, 52.5, 39.5, 34.7, 18.7, 11.3.

HRMS (ES+): calcd for $C_{30}H_{37}N_4O_3Si$ 529.2635 $[M+H]^+$, found 529.2614 $[M+H]^+$.

FT-IR (ATR): ν_{max} 2923, 2865, 2130, 2094, 1729, 1649 and 1278 cm^{-1}.

^{1}H-NMR (400 MHz, $CDCl_3$) Compound S4.

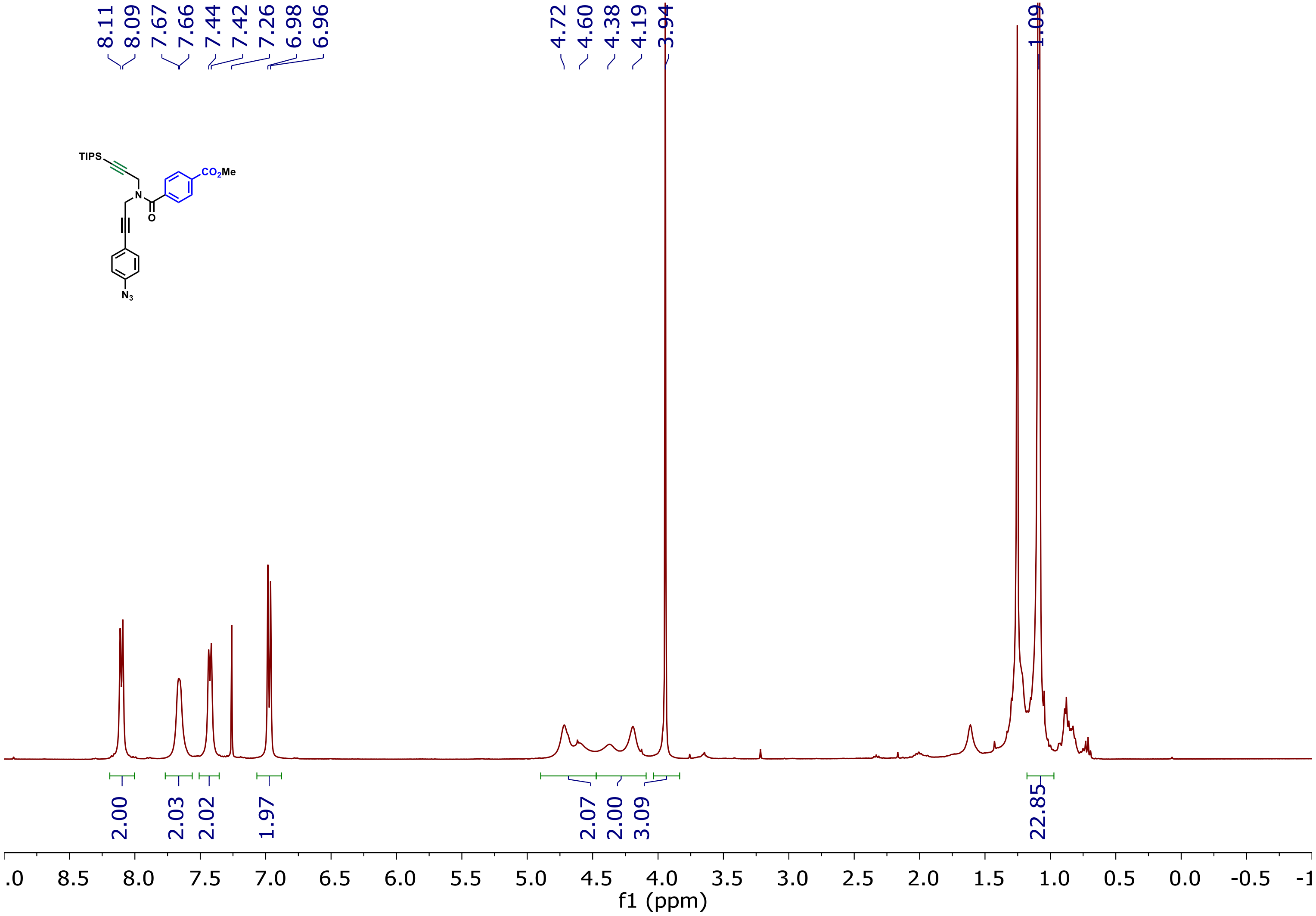

^{13}C-NMR (100.6 MHz, $CDCl_3$) Compound S4.

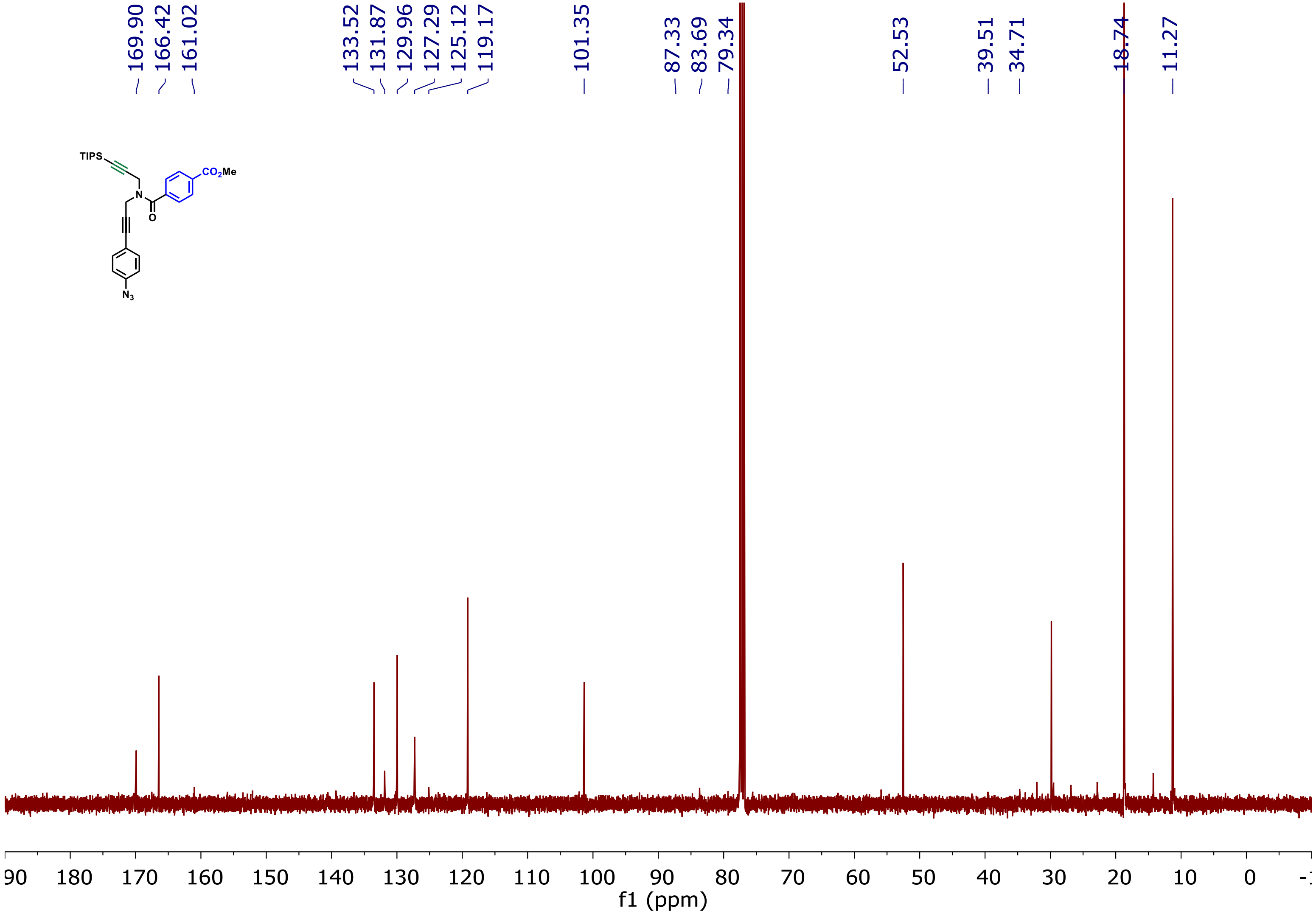

Compound S5.

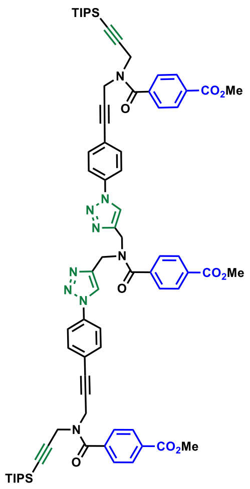

S4 (0.408 g, 0.77 mmol) and methyl 4-(di(prop-2-yn-1-yl)carbamoyl)benzoate[S3] (0.099 g, 0.39 mmol) were dissolved in anhydrous THF (5 mL) under N_2 atmosphere. Then, TBTA (20 mg, 0.04 mmol) and $Cu(CH_3CN)_4PF_6$ (15 mg, 0.04 mmol) were dissolved in THF (2 mL) and this solution added to the reaction mixture. After 16 h of stirring at room temperature, the solvent was evaporated and the crude purified by flash column chromatography on silica gel (gradient from 0% to 70% of EtOAc in Pet. Ether) to afford **S5** (0.431 g, 85 %) as a foam.

^{1}H NMR (400 MHz, CDCl$_3$): δ_H = 8.23 and 8.14 (bs, 2H, splitting is due to the existence of rotamers around the amide bond; only a singlet would be expected in the absence of rotamers), 8.11 (d, 6H, *J* = 8.5 Hz, 3-H), 7.81 (d, 2H, *J* = 8.0 Hz, 2-H), 7.75 (d, 4H, *J* = 8.5 Hz, 2-H), 7.68 (m, 4H, 2'-H), 7.63 (m, 4H, 3'-H), 4.84, 4.76, 4.71, 4.62, 4.41 and 4.33 (bs, 12H, N-CH_2, splitting is due to the existence of rotamers around the amide bond; only three signa would be expected in the absence of rotamers)), 3.95 (s, 6H, OCH_3), 3.93 (s, 3H, OCH_3), 1.10 (s, 42H, TIPS).

^{13}C NMR (100.6 MHz, CDCl$_3$): δ_C = 171.2, 170.0, 166.4, 139.5, 139.2, 133.5, 132.0, 131.8, 130.0, 127.9, 127.3, 122.2, 121.3, 120.3, 101.2, 86.8, 85.6, 83.1, 52.6, 44.8, 44.4, 39.9, 39.6, 34.9, 18.8, 11.3.

HRMS (ES+): calcd for $C_{75}H_{86}N_9O_9Si_2$ 1312.6087 $[M+H]^+$, found 1312.5963 $[M+H]^+$.

FT-IR (ATR): ν_{max} 2944, 2864, 1723, 1638, 1434, 1276, 1248 and 751 cm^{-1}.

^{1}H-NMR (400 MHz, $CDCl_3$) Compound S5.

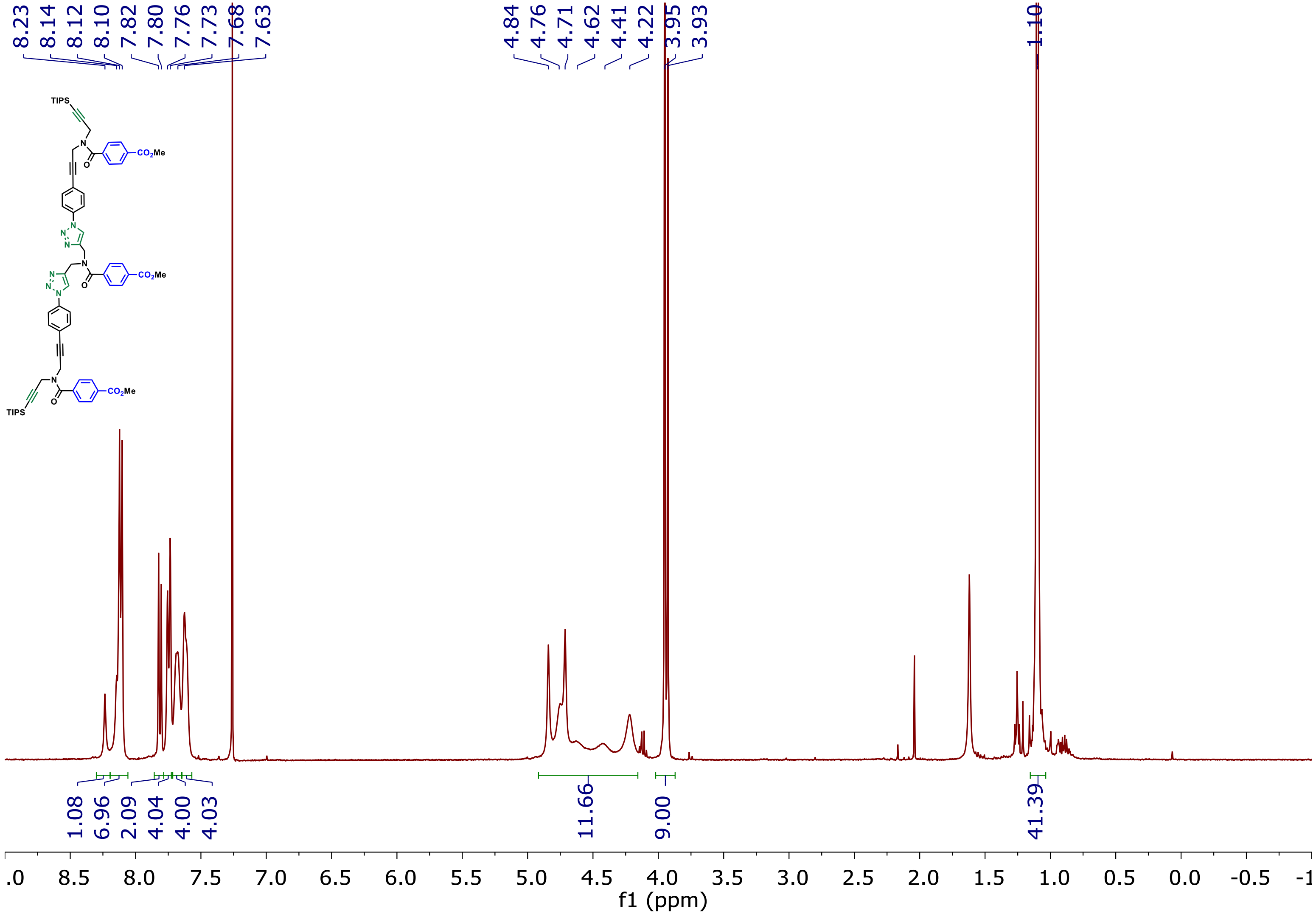

^{13}C-NMR (100.6 MHz, $CDCl_3$) Compound S5.

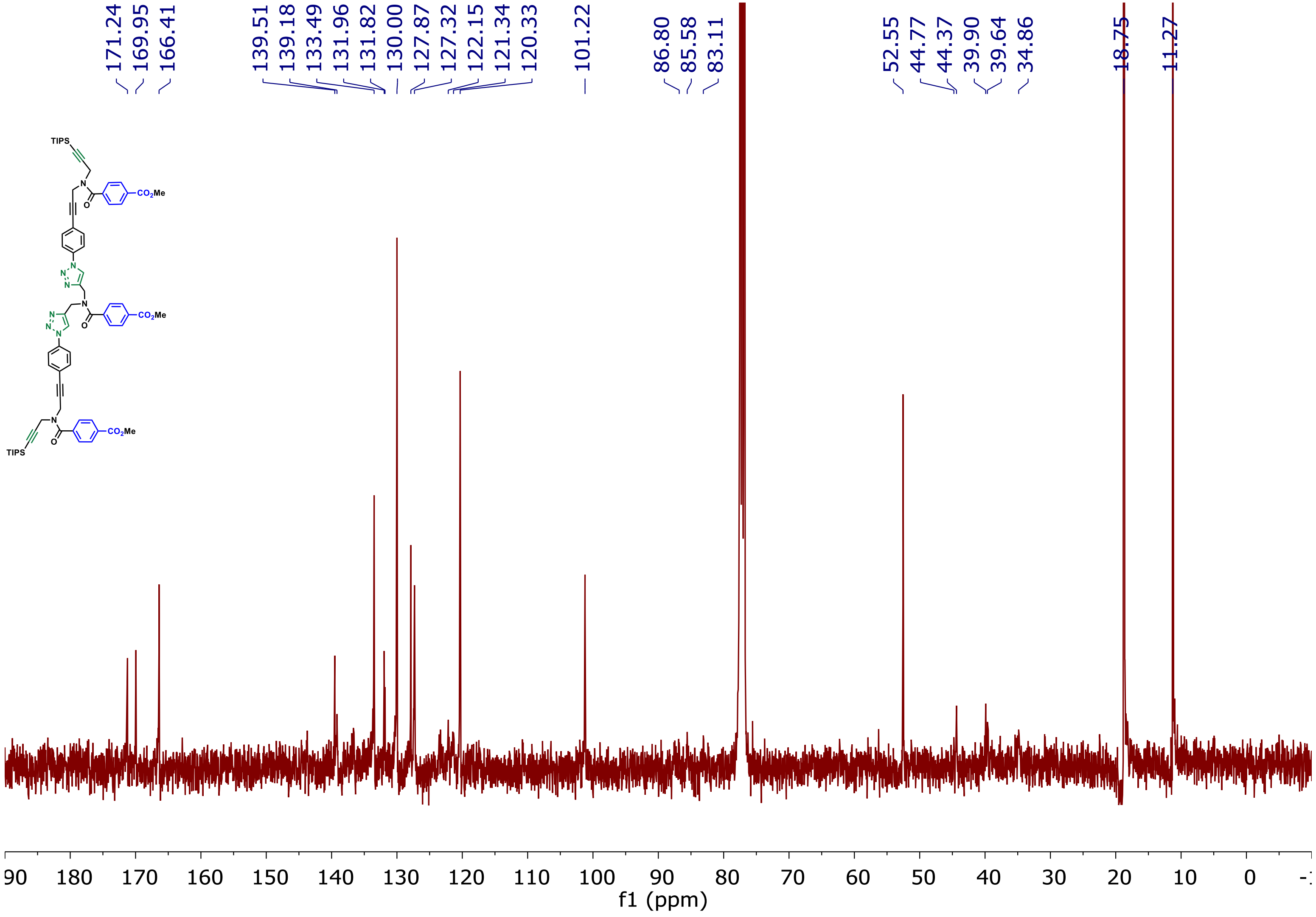

Compound S6.

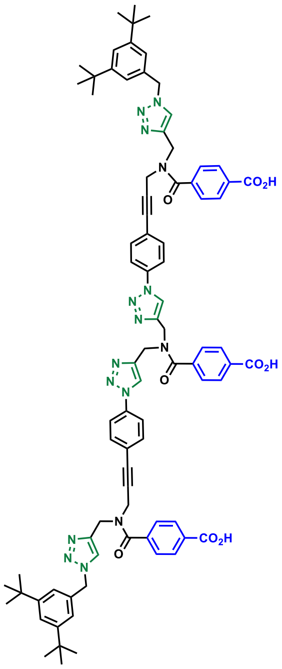

To a solution of **S5** (0.134 g, 0.10 mmol) in MeOH (2.5 mL) was added NaOH 2 N solution (0.500 mL, 1.00 mmol). The reaction was stirred overnight at room temperature and then the solution was carefully quenched with 5% dilute HCl and extracted with EtOAc (3x) followed by washing with H_2O and brine. The organic layer was dried over anhydrous $MgSO_4$ and concentrate under vacuum, affording carboxylic acid 3-mer template (0.130 g, quantitative), used without further purification. This derivative was dissolved in THF (2 mL) and treated with TBAF solution (1 M in THF, 0.210 mL, 0.210 mmol). The reaction was stirred for 48h and quenched with 5% soln. HCl and extracted with EtOAc (3x) followed by washing with H_2O and brine. The organic layer was dried over anhydrous $MgSO_4$ and concentrate under vacuum. The crude was dissolved in dry THF (5 mL) and $Cu(CH_3CN)_4PF_6$ (0.008 g, 0.02 mmol) and TBTA (0.011 g, 0.02 mmol) were added. A solution of 1-(azidomethyl)-3,5-di-*tert*-butylbenzene (0.075 g, 0.31 mmol) in THF (2 mL) was added and the reaction was stirred overnight at room temperature. The solvent was evaporated to dryness and the crude was purified by flash column chromatography on silica gel (gradient from 0% to 10% of MeOH with 1% AcOH in CH_2Cl_2) to afford **S6** (0.131 g, 89%) as a light-yellow solid.

Melting point: 159-160 °C.

^{1}H NMR (400 MHz, CD_3OD): δ_H = 8.44 (m, 2H), 8.07 (m, 6H), 7.72 (m, 4H), 7.60 (d, 4H, J = 8.0 Hz), 7.46 (d, 4H, J = 8.5 Hz), 7.38 (s, 2H), 7.31 (m, 4H), 7.15 (s, 4H), 5.55 (m, 4H), 4.72, 4.57, 4.30 and 3.75 (bs, 12H, partially overlapped by solvent signal), 1.22 (s, 36H).

^{13}C NMR (100.6 MHz, CD_3OD): δ_C = 173.3, 168.9, 152.8, 140.8, 136.7, 135.9, 134.2, 131.1, 130.7, 130.0, 129.6, 129.0, 128.4, 128.1, 125.7, 123.5, 123.3, 121.3, 86.4, 84.9, 55.5, 54.9, 45.8, 44.5, 41.8, 41.4, 36.2, 35.7, 31.8.

HRMS (ES+): calcd for $C_{84}H_{86}N_{15}O_9$ 1449.6766 $[M+H]^+$, found 1449.6733 $[M+H]^+$.

FT-IR (ATR): ν_{max} 2959, 2925, 2854, 1711, 1634, 1258, 1047 and 753 cm^{-1}.

^{1}H-NMR (400 MHz, CD_3OD) Compound S6.

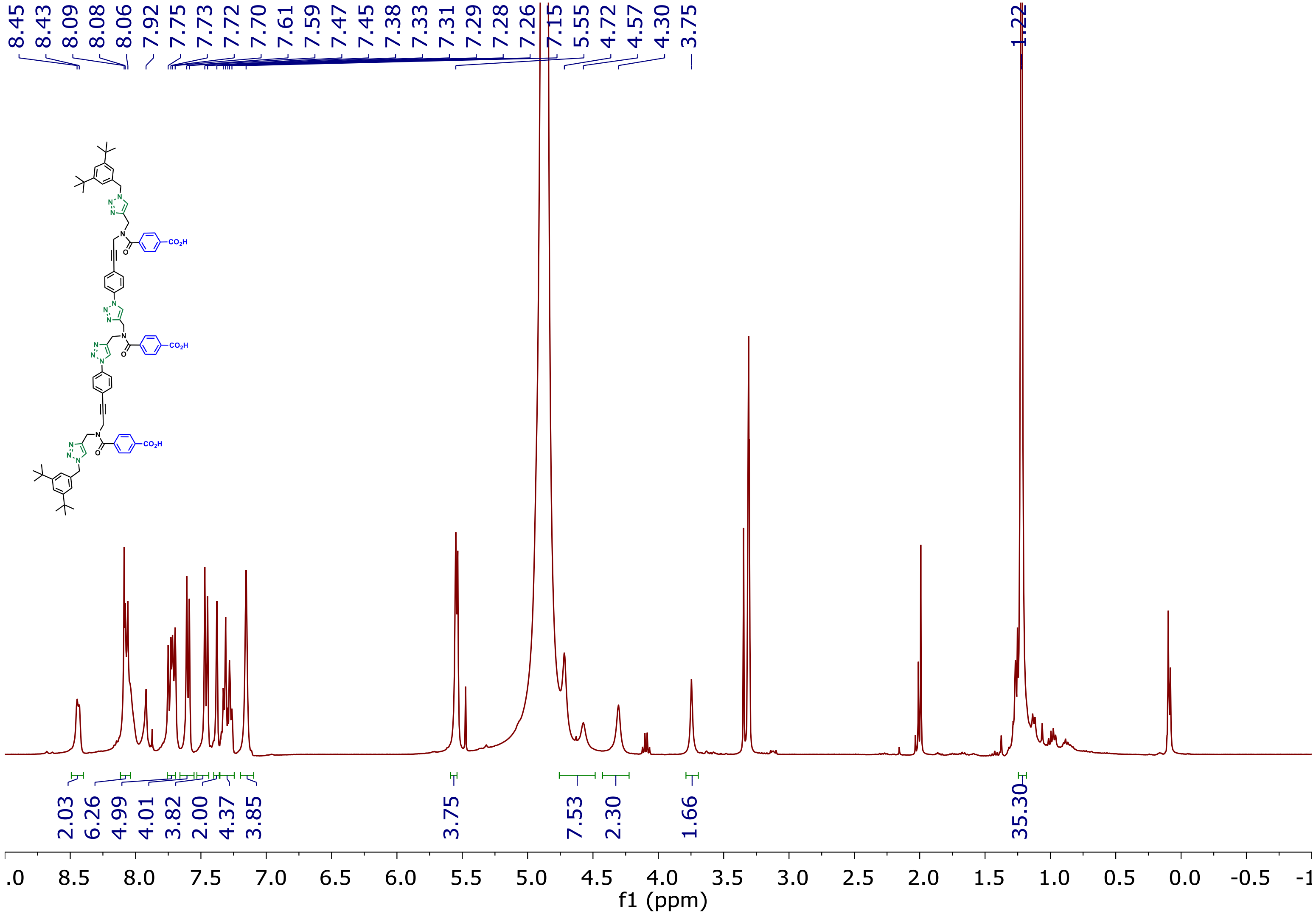

^{13}C-NMR (100.6 MHz, CD_3OD) Compound S6.

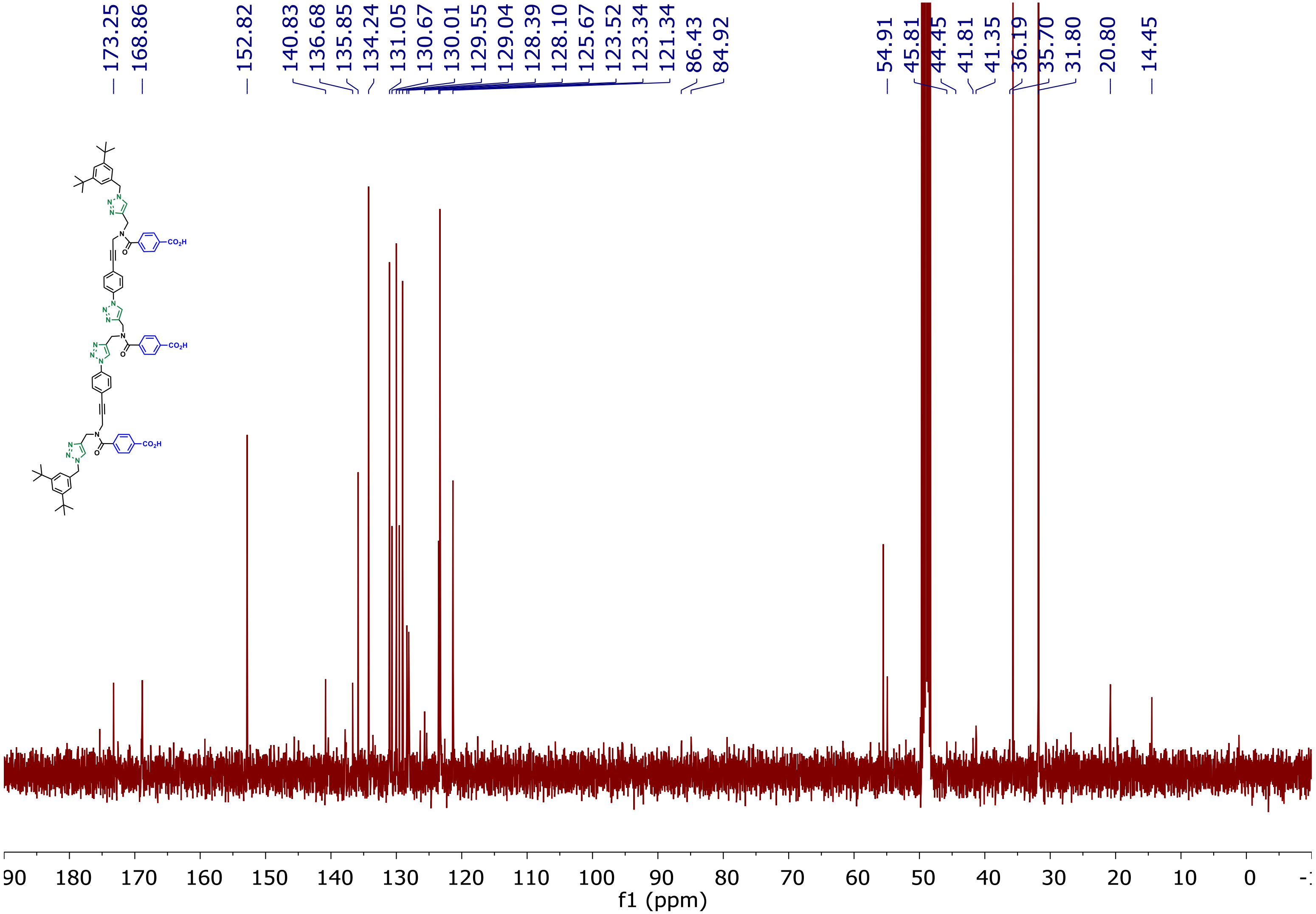

Pre-ZIP S7

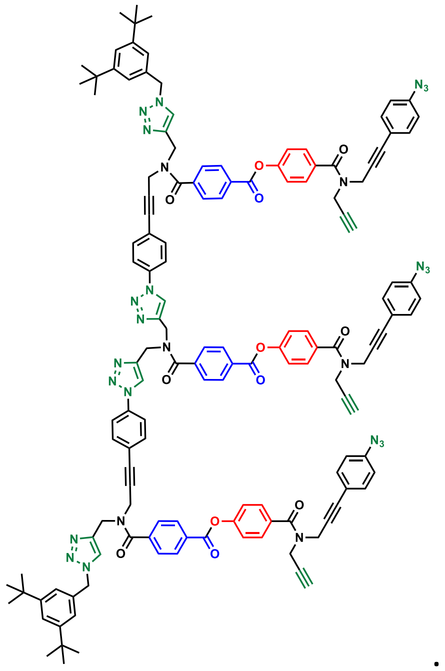

A solution of **S6** (0.026 g, 0.018 mmol), **11** (0.016 g, 0.048 mmol), EDC (0.013 g, 0.069 mmol) and DMAP (0.8 mg, 0.007 mmol) in dry CH_2Cl_2 (2 mL) under N_2 atmosphere was stirred at room temperature for 16 h. The reaction was diluted with EtOAc (5 mL) and washed with 0.1N HCl soln. (3x), H_2O (1x) and brine. The organic phase was dried with $MgSO_4$ and concentrated *in vacuo*. The crude material was purified by flash column chromatography on silica gel (gradient from 0% to 4% of MeOH in CH_2Cl_2) to afford **S7** (0.033 g, 77 %) as a light-yellow foam.

^{1}H NMR (400 MHz, CDCl$_3$): δ_H = 8.28 (m, 8H), 7.93 (d, 2H, *J* = 8.5 Hz),7.78 (m, 6H), 7.70 (m, 10H), 7.63 (m, 6H), 7.44 (m, 7H), 7.32 (m, 7H), 7.12 (s, 4H), 6.98 (d, 4H, *J* = 8.0 Hz), 5.53 (s, 4H), 4.97-4.21 (m, 24H), 2.35 (m, 3H), 1.31 (s, 36H).

^{13}C NMR (100.6 MHz, CDCl$_3$): δ_C = 171.0, 170.1, 170.0, 164.2, 164.1, 152.5, 152.0, 144.5, 143.7, 140.6, 140.5, 133.7, 133.5, 132.6, 132.6, 130.7, 129.1, 128.2, 127.6, 123.7, 123.1, 122.5, 122.1, 120.4, 119.2, 85.7, 84.0, 83.9, 83.7, 55.1, 40.3, 37.5, 35.1, 33.9, 33.5, 31.5.

HRMS (ES+): calcd for $C_{141}H_{123}N_{27}O_{12}$ 1193.4939 [M+2H]$^{+2}$, found 1193.4987 [M+2H]$^{+2}$.

FT-IR (ATR): ν_{max} 2957, 2923, 2853, 2127, 2096, 1739, 1639, 1258, 1202 and 755 cm^{-1}.

^{1}H-NMR (400 MHz, $CDCl_3$) Pre-ZIP S7.

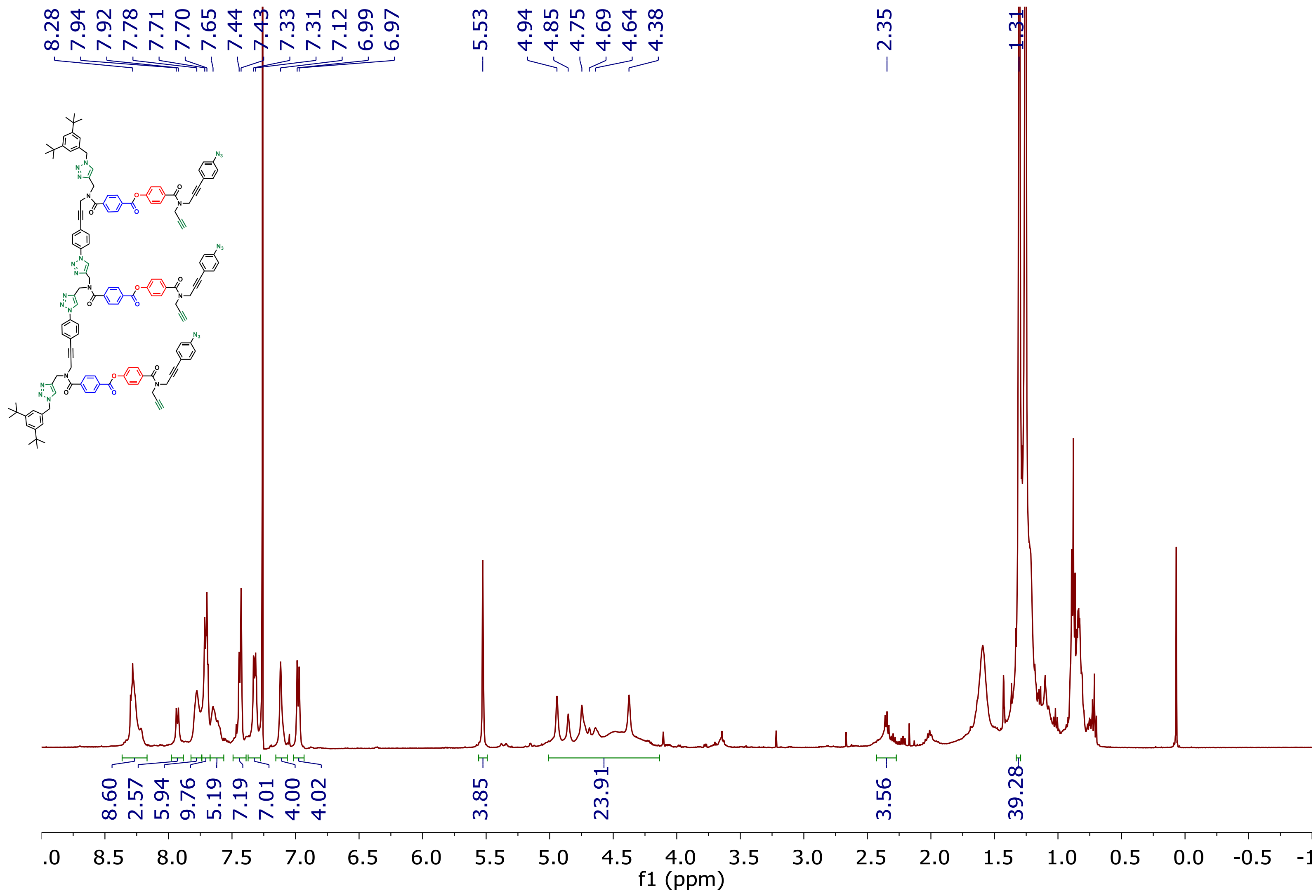

^{13}C-NMR (100.6 MHz, $CDCl_3$) Pre-ZIP S7.

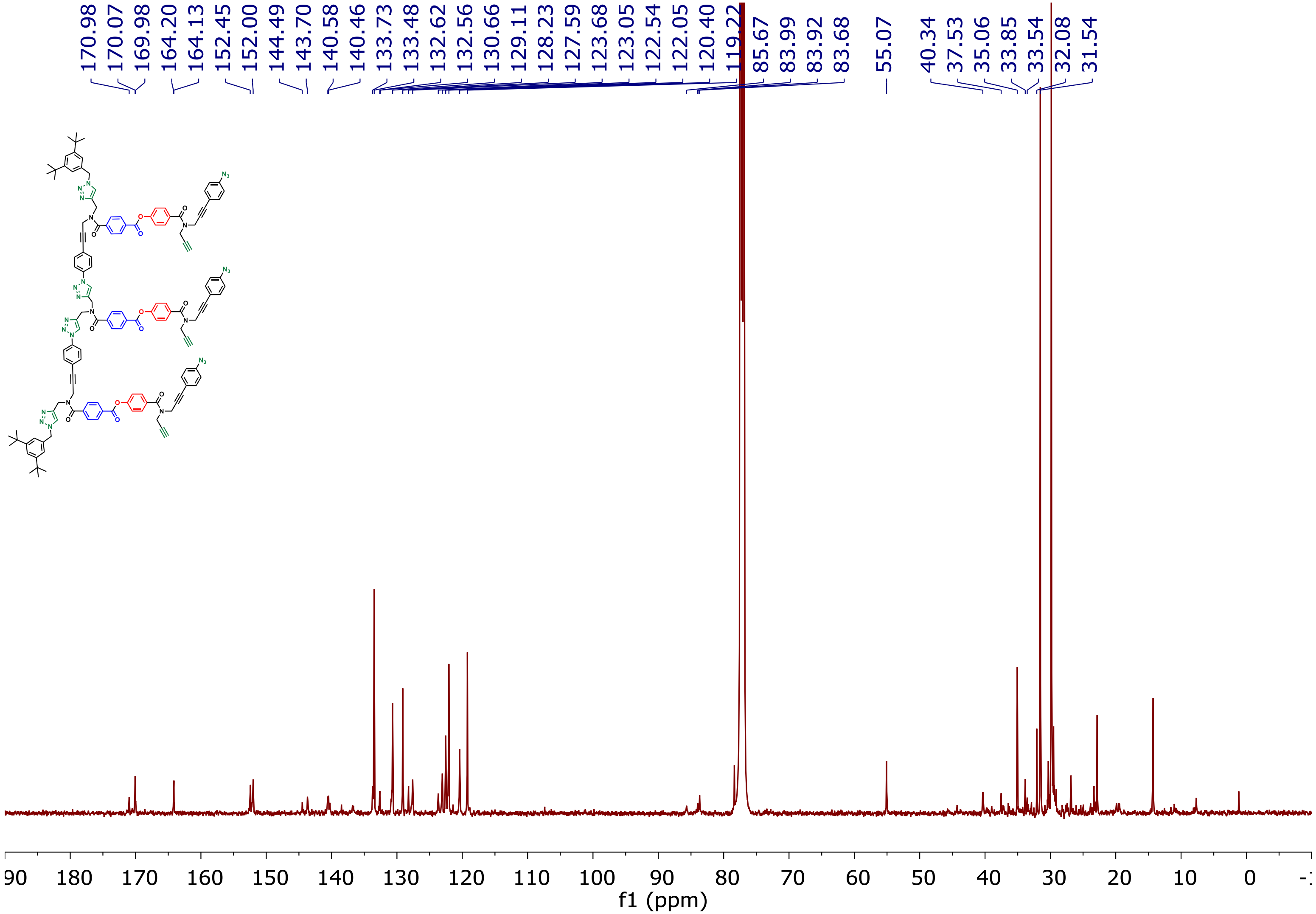

CuAAC reaction of pre-ZIP 8.

Scheme S2 shows the possible products for the CuAAC reaction of pre-ZIP **8** performed at high dilution. Firstly, the linear duplex **21** must be formed and then cyclised to form cyclic duplex **22**.

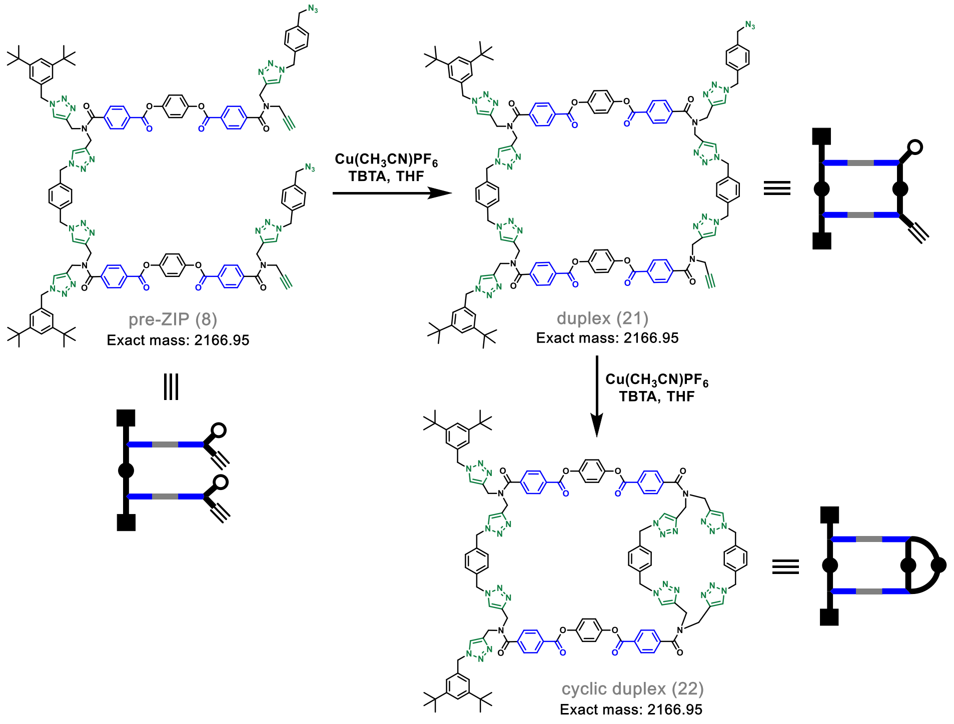

Scheme S2.

The reaction was performed as follow: Compound **8** (25 mg, 0.012 mmol) was dissolved, under N_2 atmosphere, in dry THF (50 mL). $Cu(CH_3CN)_4PF_6$ (4 mg, 0.012 mmol) and TBTA (6 mg, 0.012 mmol) were added and the reaction was stirred at room temperature under N_2 atmosphere. Aliquots of the reaction were taken at different times, diluted with EtOAc and washed with 0.2 M EDTA soln. and then analysed by UPLC to assess the progress of the reaction (Figure S1). After 21 h of reaction, the solution was diluted with EtOAc and washed with 0.02 M NaEDTA neutral aq. soln.(2x) and brine (1x). The organic layer was dried over $MgSO_4$ and the solvent evaporated to dryness. The crude was purified by flash column chromatography on silica gel (gradient from 0% to 15% of MeOH in CH_2Cl_2) to afford compound **22** (14 mg, 57%) as a white solid.

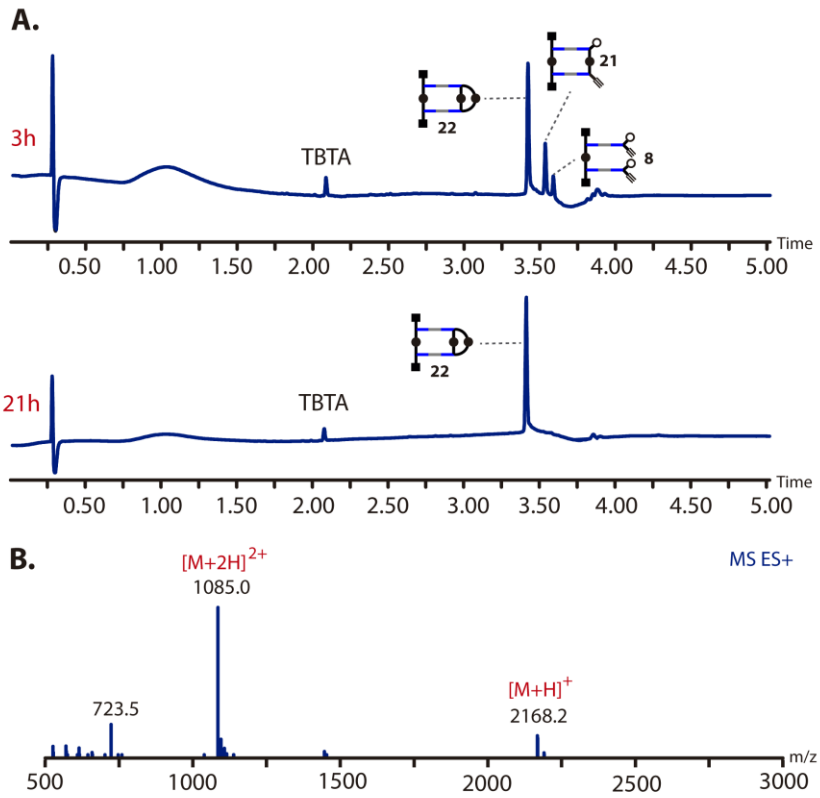

Figure S1. A. UPLC traces for the CuAAC reaction of 1,4-bis(triazolyl)methylbenzene derivative **8** (250 µM of **8** and Cu-TBTA in THF) after 3h and 21h. *Conditions:* C18 column at 40 °C (254 nm) using water + 0.1% formic acid (A) and CH_3CN + 0.1% formic acid (B) with flow rate of 0.6 ml/min; Gradient of 0-3 minutes 5% -95%B + 2 minutes 95% B. **B.** Mass spectra of **22**.

Compound **22** was fully characterised by ^1H NMR, ^{13}C NMR, IR and HRMS (see page S91). Key evidence of the formation of a cyclic product was the absence of acetylene protons in ^1H NMR and azide stretching bands in IR, when compared to the starting material **8** (Figure S2).

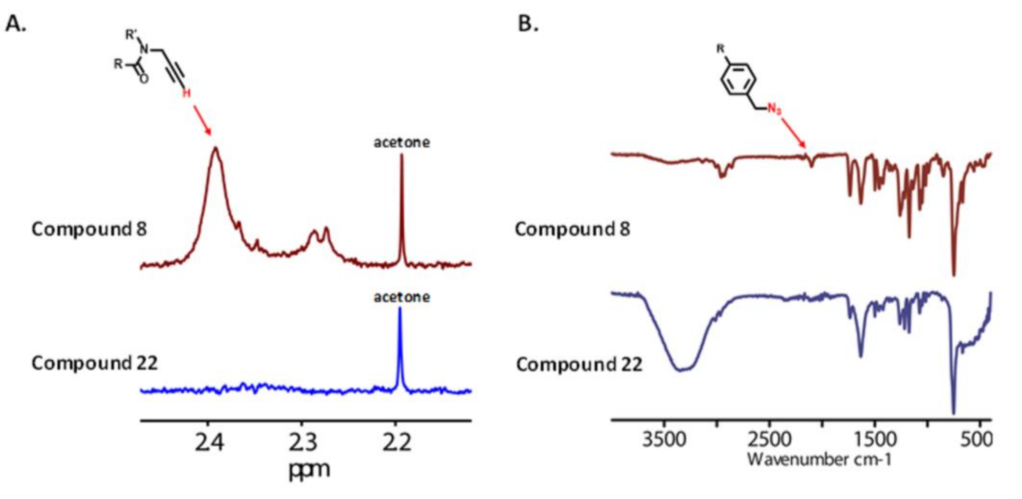

Figure S2. A. Partial ^1H NMR (400 MHz, $CDCl_3$) of pre-ZIP **8** (top) and cyclic duplex **22** (bottom), showing the absence of acetylene protons in the latter. **B.** FT-IR (ATR) of pre-ZIP **8** (top) and cyclic duplex **22** (bottom), showing the absence of azide stretching band in the latter.

As shown in Scheme S3, basic hydrolysis of **22** provided template **7** and cyclic 2-mer **23**. The reaction was performed as follow: A solution of **22** (5 mg, $2.3{\cdot}10^{-3}$ mmol) in MeOH (2 mL) was treated with 2 M NaOH solution (0.020 mL, $4.6{\cdot}10^{-2}$ mmol). After 16 h of stirring at room temperature, the solution was acidified with 1 M HCl soln. to pH 2-3 and extracted with EtOAc (3x). The combined organic phase was washed with brine, dried over $MgSO_4$ and evaporated to dryness. The obtained residue was analysed without further purification, corresponding to a mixture of cyclic-2-mer **23** and template **7** (Figure S3).

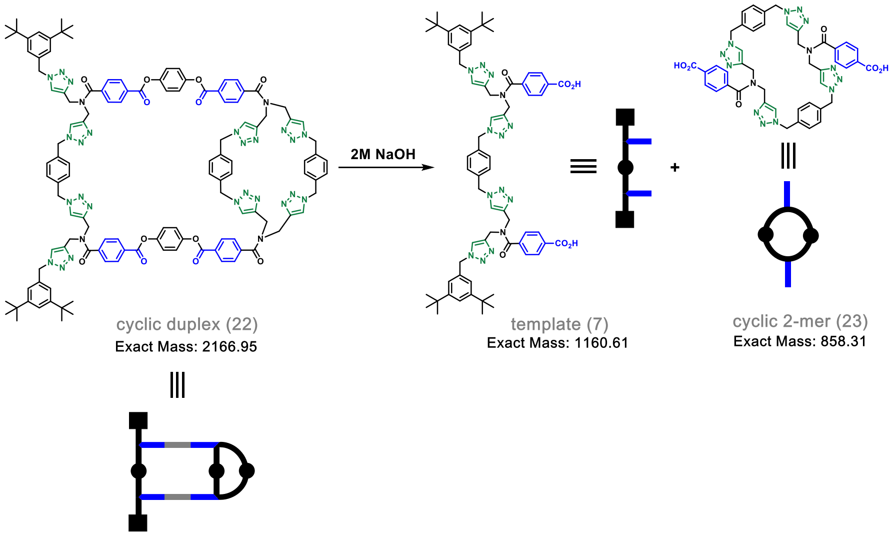

Scheme S3.

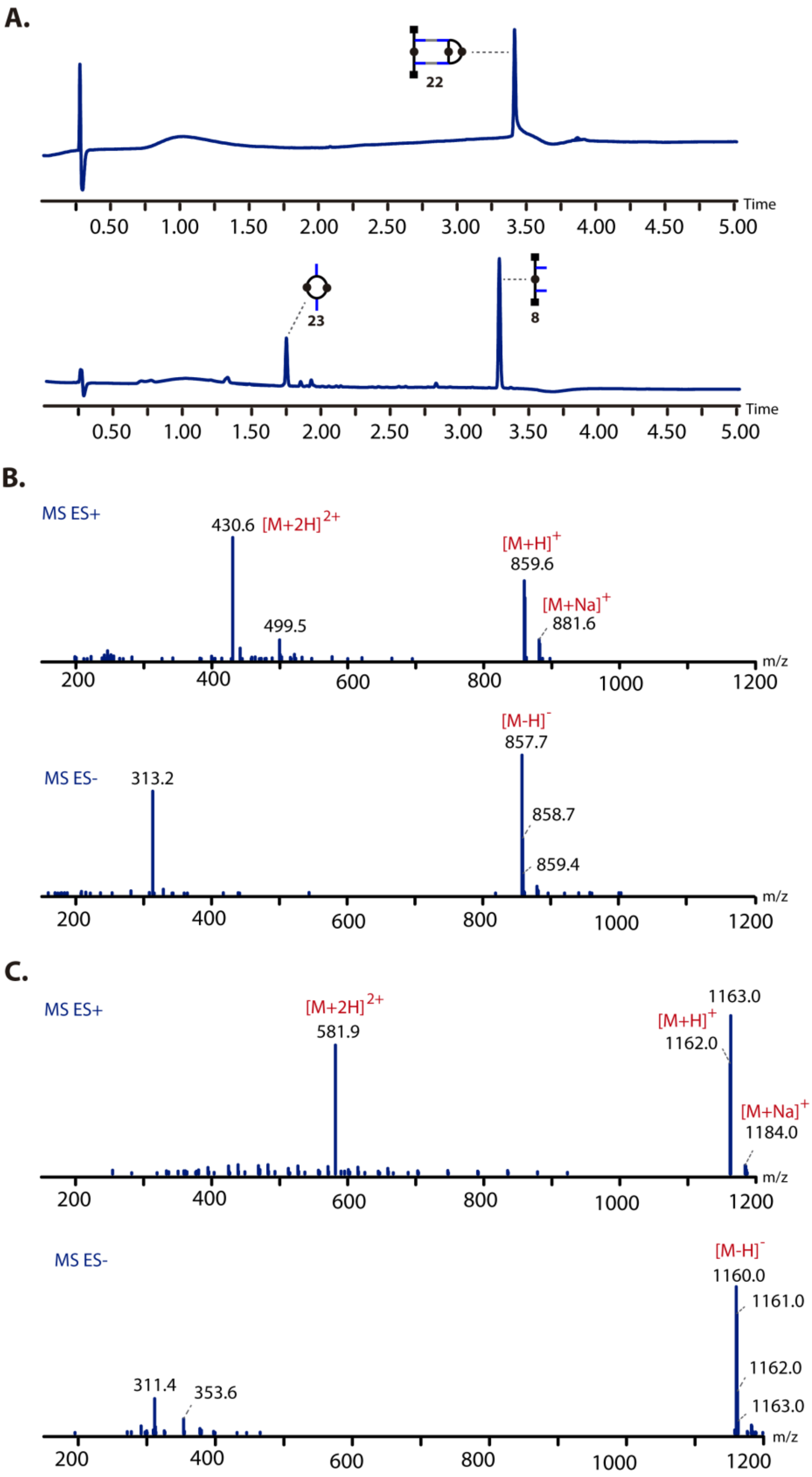

Figure S3. A. UPLC traces of cyclic duplex **22** (top) and crude of the hydrolysis of **22** (bottom). *Conditions:* C18 column at 40 °C (254 nm) using water + 0.1% formic acid (A) and CH_3CN + 0.1% formic acid (B) with flow rate of 0.6 ml/min; Gradient of 0-3 minutes 5% -95%B + 2 minutes 95% B. **B.** Mass spectra of cyclic 2-mer **23**. **C.** Mass spectra of template **8**.

CuAAC reaction of pre-ZIP 15.

Scheme S4 shows the possible products for the CuAAC reaction of pre-ZIP **15** performed at high dilution. Firstly, the linear duplex **24** must be formed both in the parallel and antiparallel arrangement as the backbone is directional. Then, further CuAAC reaction of **24** would afford cyclic duplex **25**.

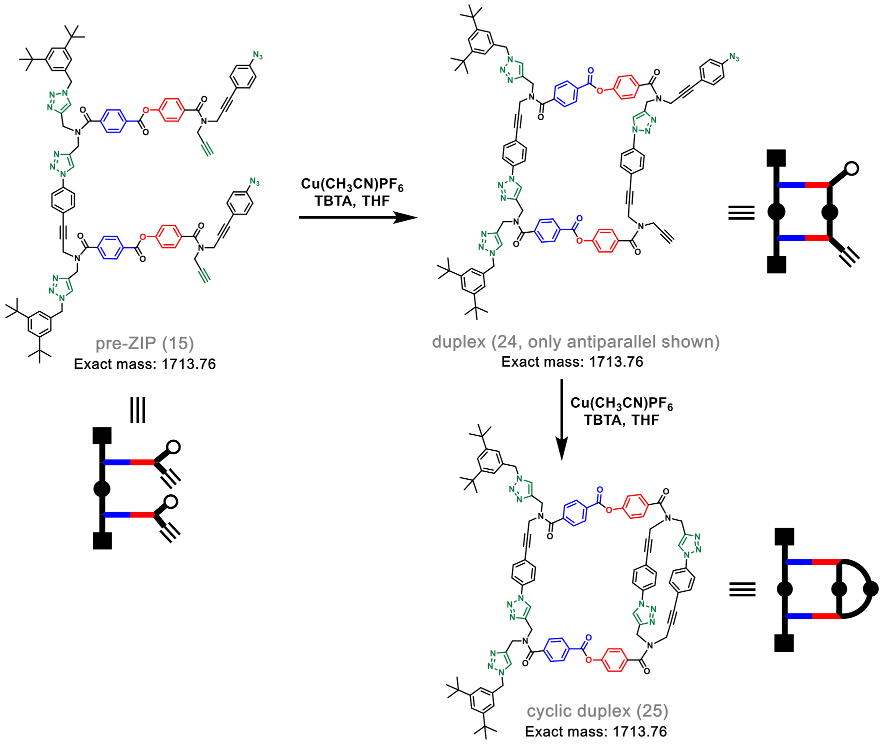

Scheme S4.

The reaction was performed as follow: Compound **15** (10 mg, $5.8{\cdot}10^{-3}$ mmol) was dissolved, under N_2 atmosphere, in dry THF (120 mL). $Cu(CH_3CN)_4PF_6$ (2 mg, $5.8{\cdot}10^{-3}$ mmol) and TBTA (3 mg, $5.8{\cdot}10^{-3}$ mmol) were added and the reaction was stirred at room temperature under N_2 atmosphere. Aliquots of the reaction were taken at different times, diluted with EtOAc and washed with 0.2 M EDTA soln. and then analysed by UPLC to assess the progress of the reaction (Figure S4). After 96 h of reaction, the solvent was evaporated and the reaction mixture was purified by flash column chromatography on silica gel (gradient from 0% to 5% of MeOH in CH_2Cl_2). The fractions corresponding to the product were combined, dried and filtered through a pad of silica using EtOAc as solvent, affording cyclic duplex **25** (7 mg, 70%).

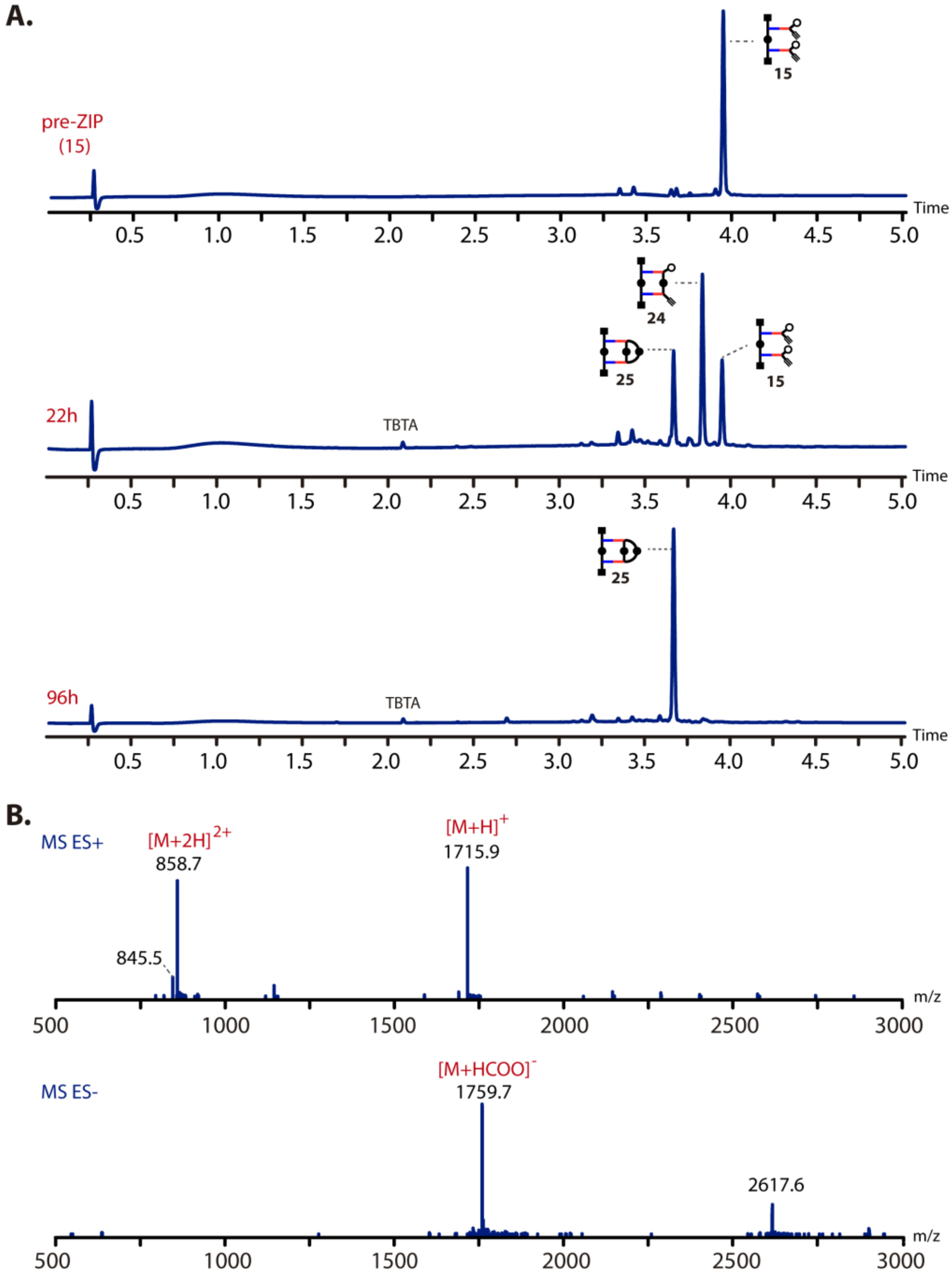

Figure S4. A. UPLC traces for the CuAAC reaction of *p*-triazolylethynylbenzene derivative **15** (50 µM of **15** and Cu-TBTA in THF) after 22 h and 96 h. *Conditions:* C18 column at 40 °C (254 nm) using water + 0.1% formic acid (A) and CH_3CN + 0.1% formic acid (B) with flow rate of 0.6 ml/min; Gradient of 0-3 minutes 5% -95%B + 2 minutes 95% B. **B.** Mass spectra of **25**.

Compound **25** was fully characterised by ^{1}H NMR, ^{13}C NMR, IR and HRMS (see page S94). Key evidence of the formation of a cyclic product was the absence of acetylene protons in ^{1}H NMR and azide stretching bands in IR, when compared to the starting material **15** (Figure S5).

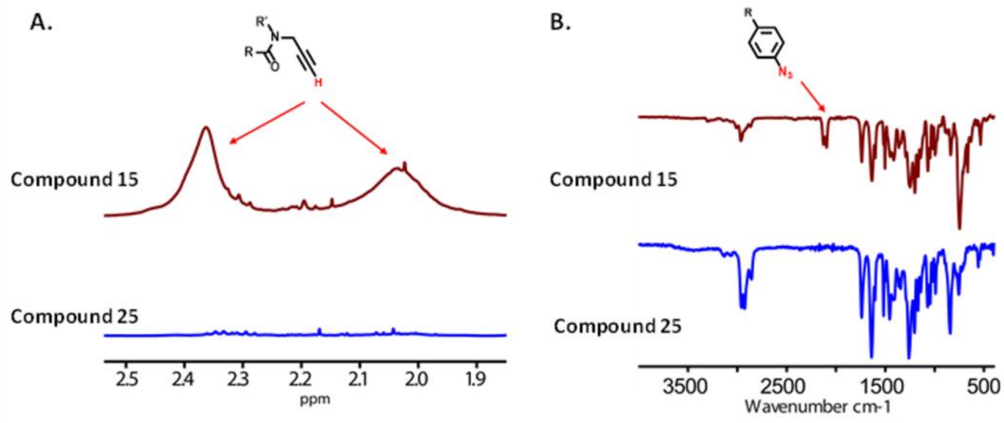

Figure S5. A. Partial ^{1}H NMR (400 MHz, $CDCl_3$) of pre-ZIP **15** (top) and cyclic duplex **24** (bottom), showing the absence of acetylene protons in the latter. **B.** FT-IR (ATR) of pre-ZIP **15** (top) and cyclic duplex **25** (bottom), showing the absence of azide stretching band in the latter.

As shown in Scheme S5, basic hydrolysis of **25** provided template **14** and cyclic 2-mer **26**. The reaction was performed as follow: A solution of **25** (5 mg, $2.9{\cdot}10^{-3}$ mmol) in MeOH (1 mL) was treated with 2 M NaOH solution (0.030 mL, $5.8{\cdot}10^{-2}$ mmol). After 20 min of stirring at room temperature, the solution was acidified with 1 M HCl soln. to pH 2-3 and extracted with EtOAc (3x). The combined organic phase was washed with brine, dried over $MgSO_4$ and evaporated to dryness. The obtained residue was analysed without further purification, corresponding to a mixture of cyclic-2-mer **26** and template **14** (Figure S6).

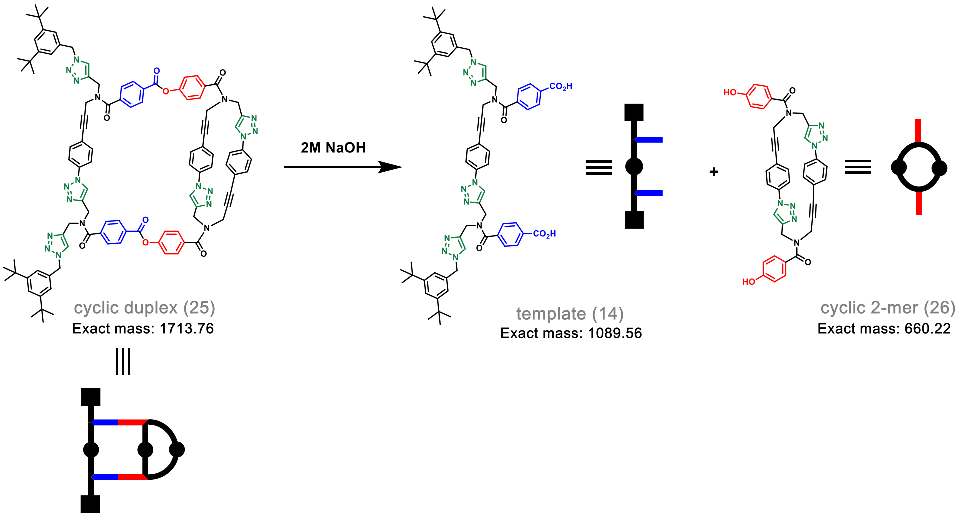

Scheme S5.

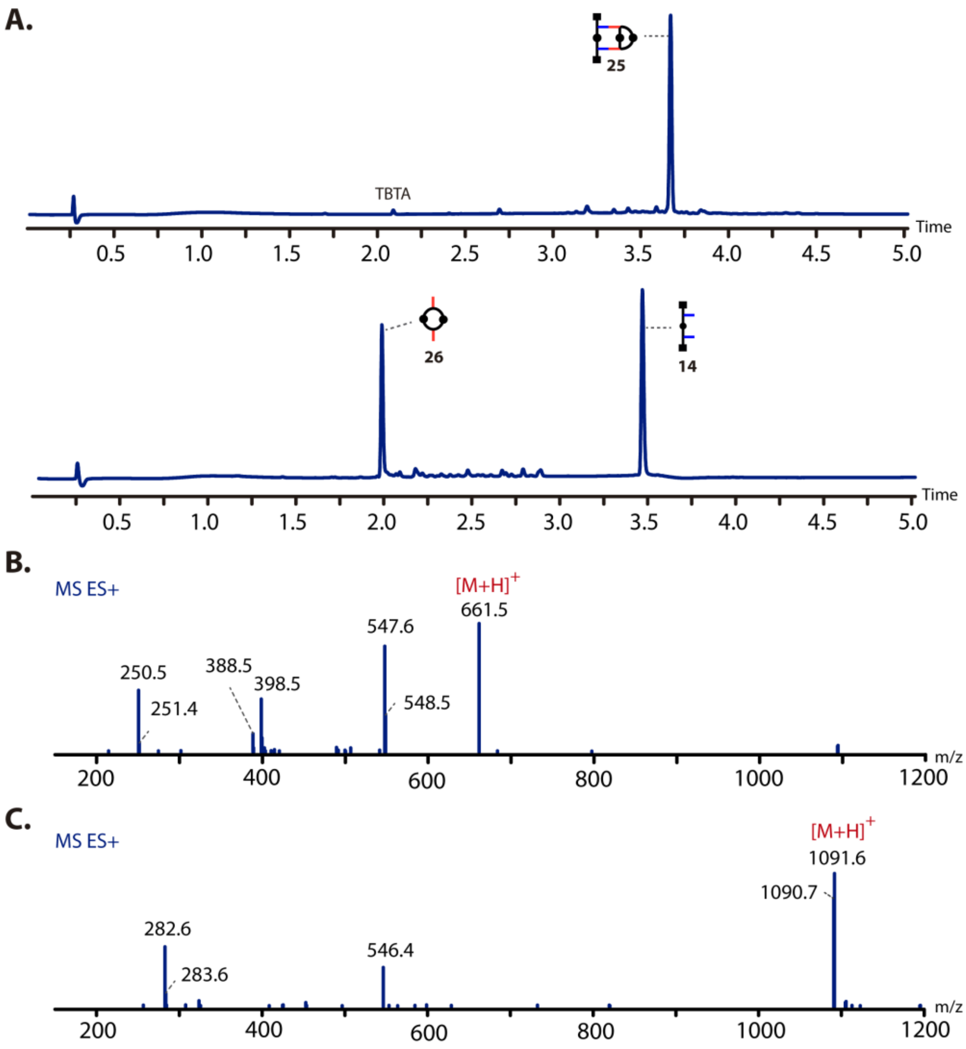

Figure S6. A. UPLC traces of cyclic duplex **25** (top) and crude of the hydrolysis of **25** (bottom). *Conditions:* C18 column at 40 ^{o}C (254 nm) using water + 0.1% formic acid (A) and CH_3CN + 0.1% formic acid (B) with flow rate of 0.6 ml/min; Gradient of 0-3 minutes 5% -95%B + 2 minutes 95% B. **B.** Mass spectra of cyclic 2-mer **26**. **C.** Mass spectra of template **14**.

CuAAC reaction of pre-ZIP 20.

Scheme S6 shows the possible products for the CuAAC reaction of pre-ZIP **20** performed at high dilution. Firstly, the linear duplex **27** must be formed both in the parallel and antiparallel arrangement as the backbone is directional. Then, further CuAAC reaction of **27** would lead to intermolecular adducts such as **28**. Cyclisation of the linear duplex **27** to form cyclic 2-mer duplex is not possible due to the high ring strain associated to such process.

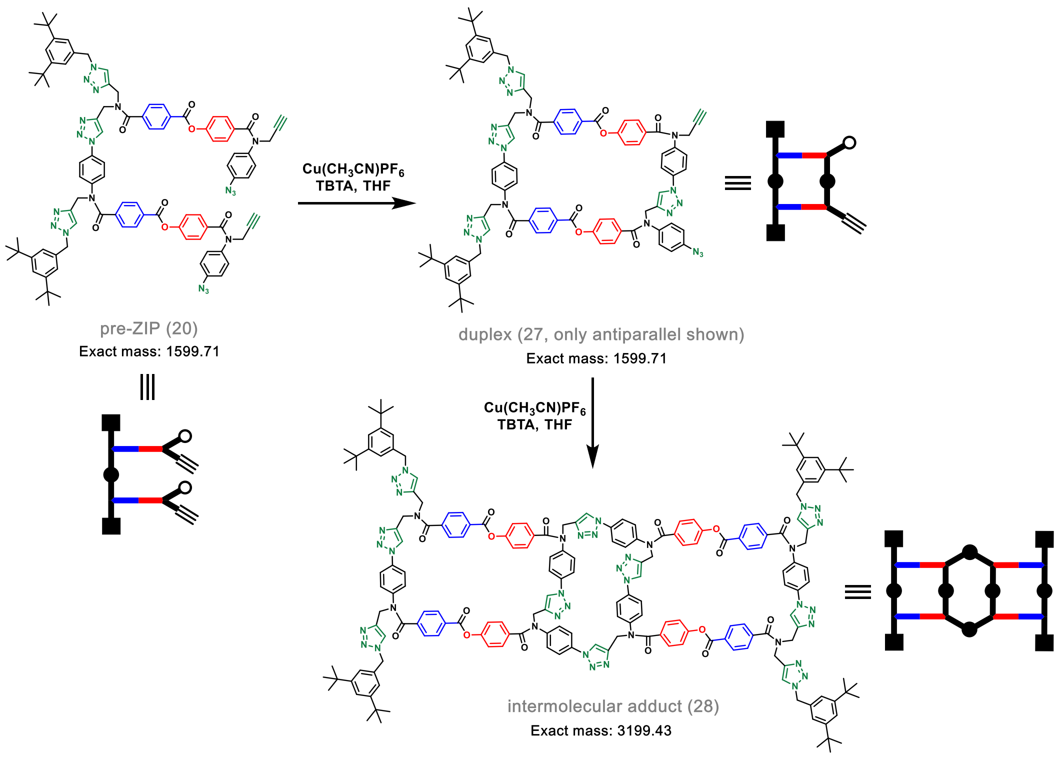

Scheme S6.

The reaction was performed as follow: Compound **20** (4 mg, $2.5 \cdot 10^{-3}$ mmol) was dissolved, under N_2 atmosphere, in dry THF (50 mL). $Cu(CH_3CN)_4PF_6$ (0.9 mg, $2.5 \cdot 10^{-3}$ mmol) and TBTA (1.2 mg, $2.5 \cdot 10^{-3}$ mmol) were added and the reaction was stirred at room temperature under N_2 atmosphere. Aliquots of the reaction were taken at different times, diluted with EtOAc and washed with 0.2 M EDTA soln. and then analysed by UPLC to assess the progress of the reaction (Figure S7). After 22 h of reaction, duplex **27** is the major product formed and correspond to the linear duplex structure bearing alkyne and azide functionalities because it accumulates and is then consumed. Mass spectra of **27** is shown in Figure S8A. After 9 days of reaction, **27** reacted completely to give intermolecular adducts, such as **28**, although multiple adducts are possible. Confirmation of the formation of intermolecular adducts is given by the mass spectra shown in Figure S8B. The triply charged ion with a mass of 1068.2 can only correspond to an intermolecular adduct with molecular mass of 3199.4.

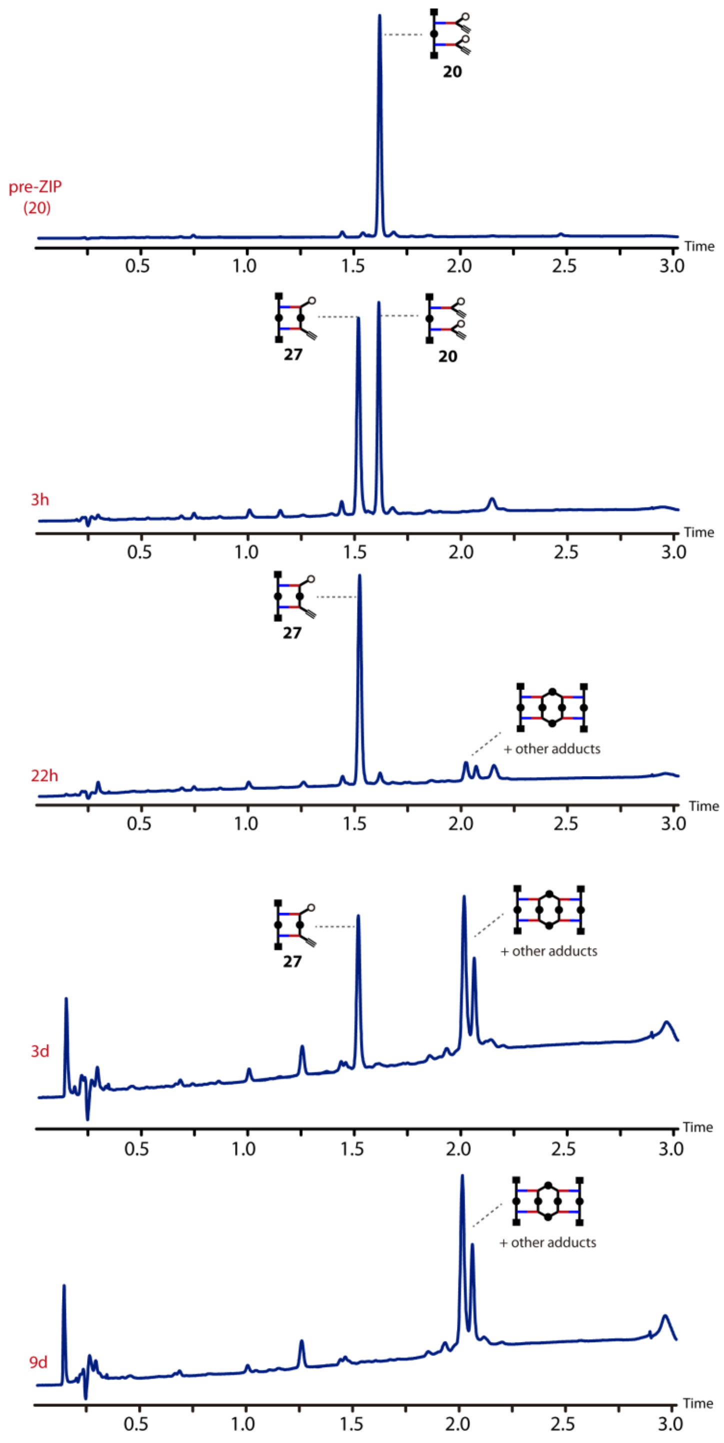

Figure S7. UPLC traces for the CuAAC reaction of *p*-triazolylaniline derivative **20** (50 µM of **20** and Cu-TBTA in THF) after 3h, 22 h, 3 days and 9 days. *Conditions:* C18 column at 40 ºC (254 nm) using water + 0.1% formic acid (A) and CH_3CN + 0.1% formic acid (B) with flow rate of 0.6 ml/min; Gradient of 0-2 minutes 65% -100%B + 1 minute 100% B.

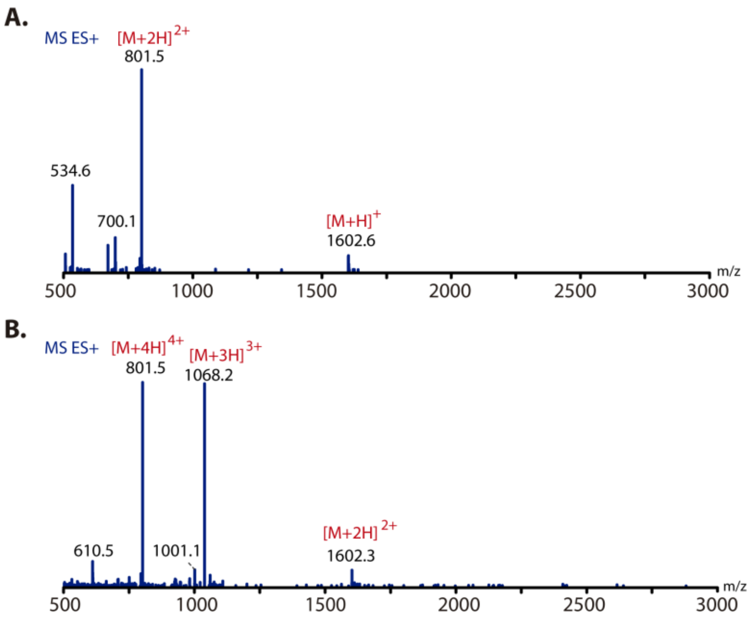

Figure S8. A. Mass spectra of CuAAC crude after 22 h, corresponding to linear duplex **27**. **B.** Mass spectra of CuAAC crude after 9 days, corresponding to intermolecular adduct **28**.

Basic hydrolysis of the aliquots taken from the reaction at different times proved to be an alternative and efficient way to analyse the progress of the reaction. As shown in Scheme S7, basic hydrolysis of the aliquot taken after 22 h of reaction, containing **27** as the major product, would provide template **18** and linear 2-mer **26**. In contrast, basic hydrolysis of the aliquot taken after 9 days of reaction, containing intermolecular adducts such as **28**, would provide template **18** and cyclic 4-mer **29** among larger cyclic species.

The reaction was performed as follow: Aliquots taken from the reaction at different times were diluted with EtOAc, washed with 0.2 M EDTA soln. and the solvent evaporated. The crude was dissolved in THF (1 mL) and 1 M LiOH soln. (3-4 drops) was added. After 15 min of reaction at room temperature, the reaction was quenched with diluted HCl soln., extracted with EtOAc and analyse by UPLC. Figure S9A shows the chromatogram of the crude after hydrolysis of the aliquot taken after 22 h of reaction (top), and the mass spectra of the peak with a retention time of 1.94 min, corresponding to the linear 2-mer. Figure S9B shows the chromatogram of the crude after hydrolysis of the aliquot taken after 9 days of reaction (top), and the mass spectra of the peak with a retention time of 1.61 min, corresponding to the cyclic 4-mer **29**.

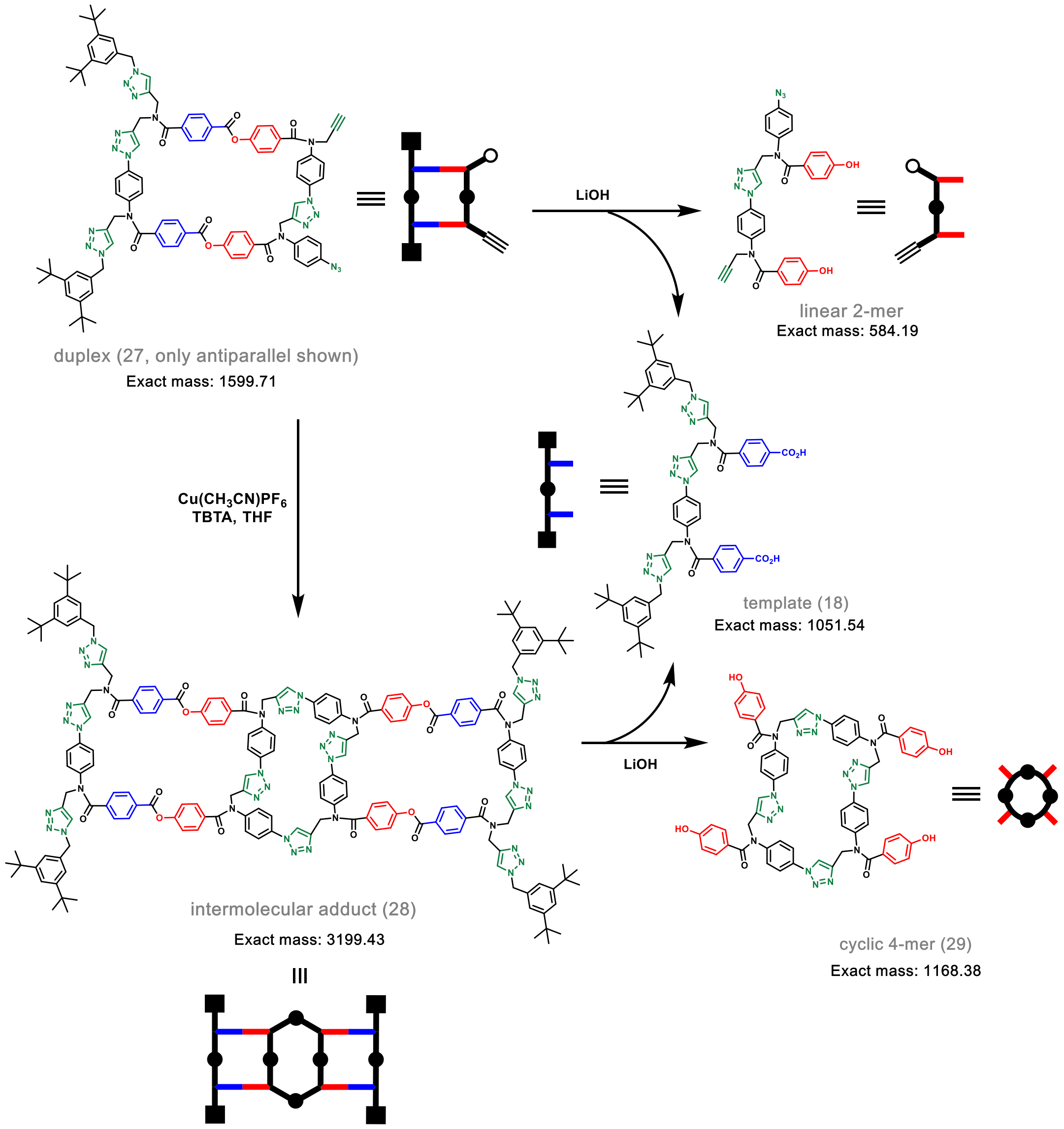
LiOH
duplex (27, only antiparallel shown)
Exact mass: 1599.71
linear 2-mer
Exact mass: 584.19
Cu(CH3CN)PF6
TBTA, THF
template (18)
Exact mass: 1051.54
LiOH
intermolecular adduct (28)
Exact mass: 3199.43
cyclic 4-mer (29)
Exact mass: 1168.38

Scheme S7.

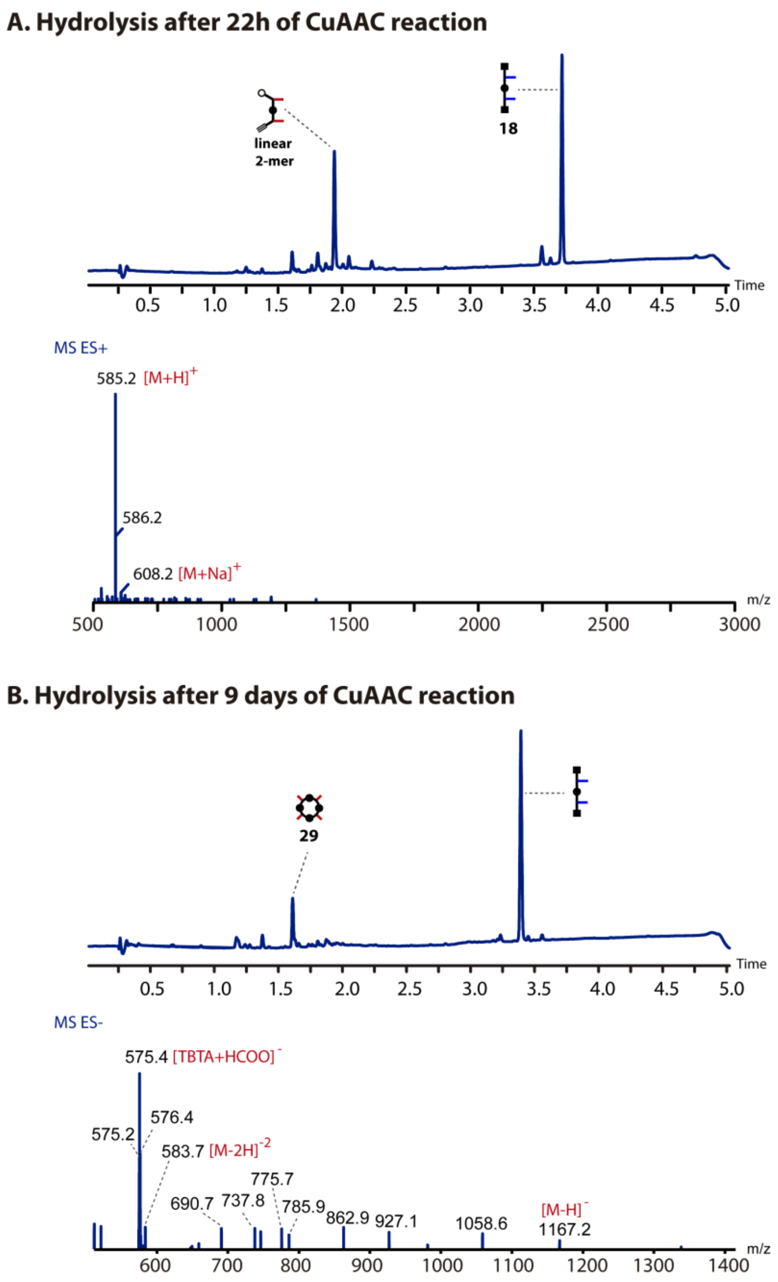

Figure S9. A. UPLC trace for the hydrolysis of CuAAC reaction crude of **20** after 22 h (top) and mass spectra of linear 2-mer (bottom). **B.** UPLC trace for the hydrolysis of CuAAC reaction crude of **20** after 9 days (top) and mass spectra of cyclic 4-mer **29** (bottom). *Conditions:* C18 column at 40 °C (254 nm) using water + 0.1% formic acid (A) and CH_3CN + 0.1% formic acid (B) with flow rate of 0.6 ml/min; Gradient of 0-4 minutes 5% -100%B + 1 minute 100% B.

CuAAC reaction of pre-ZIP S7.

Scheme S8 shows a schematic representation of the ZIP reaction for the trimer Pre-ZIP **S7**.

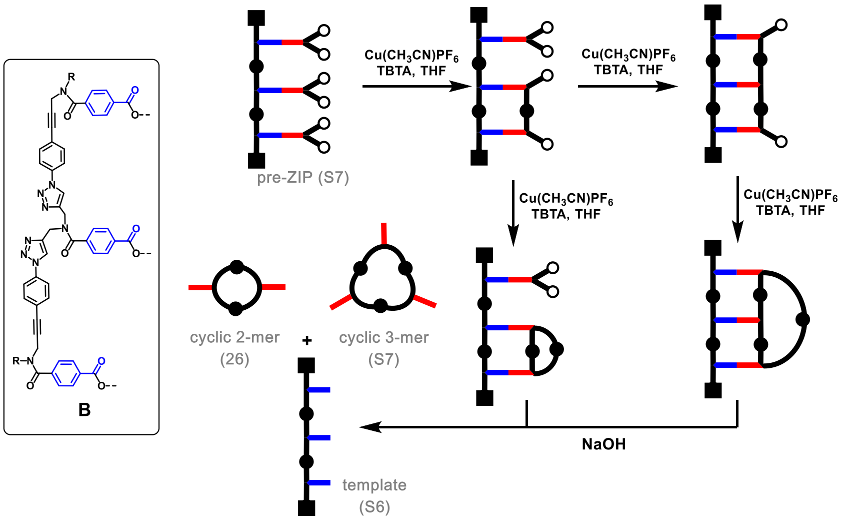

Scheme S8.

The reaction was performed as follow: Compound **S7** (5 mg, $5.8 \cdot 10^{-3}$ mmol) was dissolved, under N_2 atmosphere, in dry THF (120 mL). $Cu(CH_3CN)_4PF_6$ (2 mg, $5.8 \cdot 10^{-3}$ mmol) and TBTA (3 mg, $5.8 \cdot 10^{-3}$ mmol) were added and the reaction was stirred at room temperature under N_2 atmosphere. Aliquots of the reaction were taken at different times, diluted with EtOAc and washed with 0.2 M EDTA soln. and then analysed by UPLC to assess the progress of the reaction (Figure S10).

After 64 h of reaction. basic hydrolysis of the ZIP product provided template **S6** and a mixture of cyclic 2-mer **26** and cyclic 3-mer **S8**. The reaction was performed as follow: A solution of ZIP product (3 mg, $2.9 \cdot 10^{-3}$ mmol) in MeOH (1 mL) was treated with 2 M NaOH solution (0.030 mL, $5.8 \cdot 10^{-2}$ mmol). After 20 min of stirring at room temperature, the solution was acidified with 1 M HCl soln. to pH 2-3 and extracted with EtOAc (3x). The combined organic phase was washed with brine, dried over $MgSO_4$ and evaporated to dryness. The obtained residue was analysed without further purification, corresponding to a mixture of cyclic-2-mer **26**, cyclic 3-mer **S7** and template **14** (Figure S10).

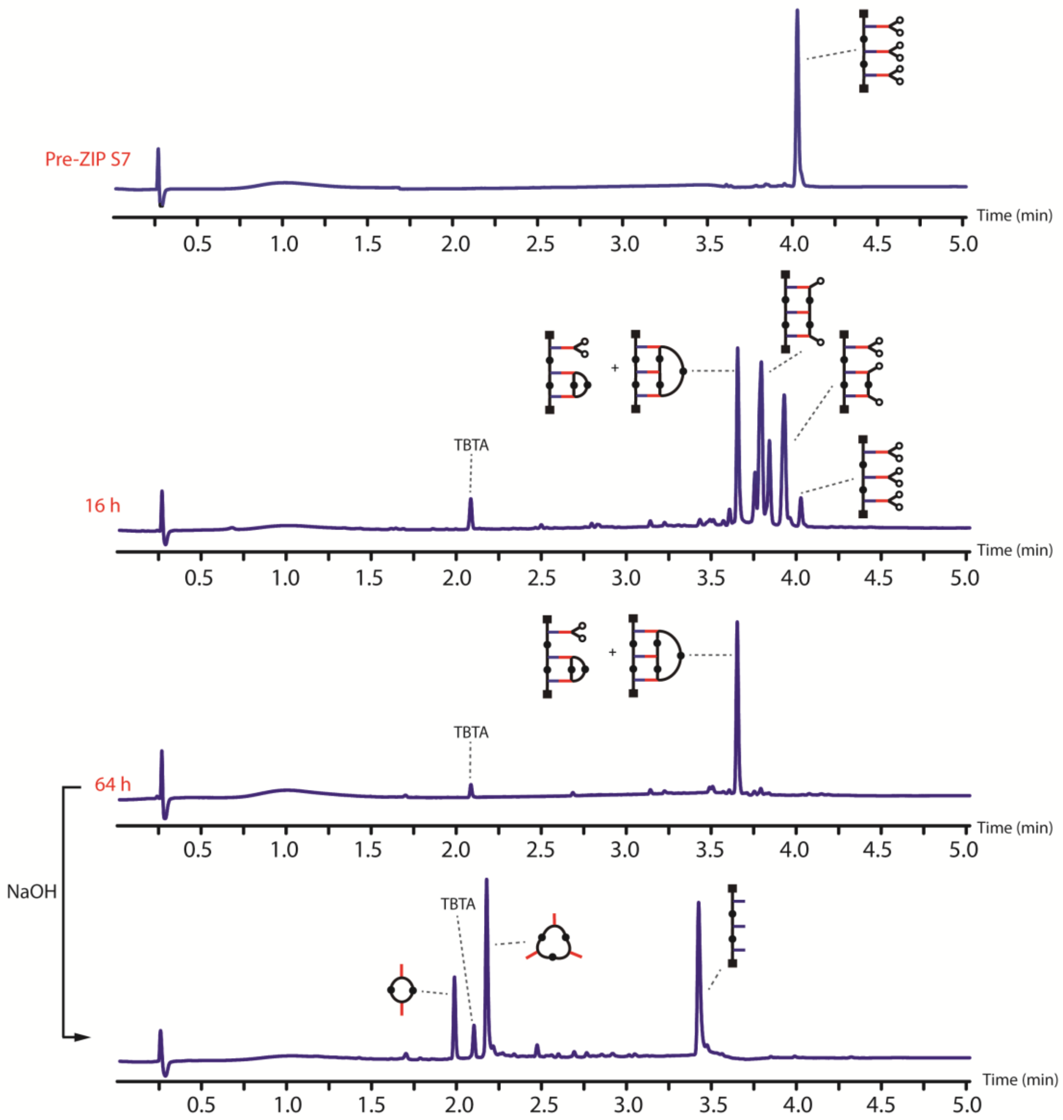

Figure S10. UPLC traces for the CuAAC reaction of pre-ZIP **S7** (50 µM in THF) after 16 h and 64 h, and after subsequent hydrolysis. *Conditions:* C18 column at 40 ºC (254 nm) using water + 0.1% formic acid (A) and CH_3CN + 0.1% formic acid (B); Gradient of 0-3 minutes 5% -95%B + 2 minutes 95% B.

Molecular modelling calculations

General details

Molecular mechanics calculations were performed using MacroModel implemented in Maestro 11 (Schrodinger release 2016-4).[S7] Structures were simplified by replacing the capping groups by methyl and phenyl groups. The structures were minimized first, and the minimized structures were then used as the starting molecular structure for the MacroModel conformational search. The force field used was MMFFs with $CHCl_3$ solvation. The charges were defined by the force field library and no cut off was used for non-covalent interactions. A Polak-Ribiere Conjugate Gradient (PRCG) was used and each minimisation was subjected to 10.000 iterations with a convergence threshold of 0.01. Conformational searches were performed using 100 steps per rotatable bond (maximum number of steps of 10.000). Images were created using PyMol.[S8]

Estimation of the ring strain.

The ring strain for the ZIP and 2-mer macrocyclization reactions was estimated by using the strain energy of the product (E_{strain}), as shown in Scheme S9. The disconnection of the triazole rings into the corresponding azide and alkyne was not used due to problems in the parameterisation of azide moieties in the MMFFs force-field. The disconnection of the macrocyclic structures was done through the benzyl-triazole or phenyl-triazole bonds. This hypothetical transformation provides a method for calculating E_{Bond} as the energy difference between two fragments and the connected oligomer backbone (Equation 1). The energy contribution associated with benzyl-triazole or phenyl-triazole bond connection (E_{bond}) was subtracted from the difference between the energy of the macrocyclic product ($E_{macrocycle}$) and the energy of the linear intermediate (E_{linear}) (Equation 2).

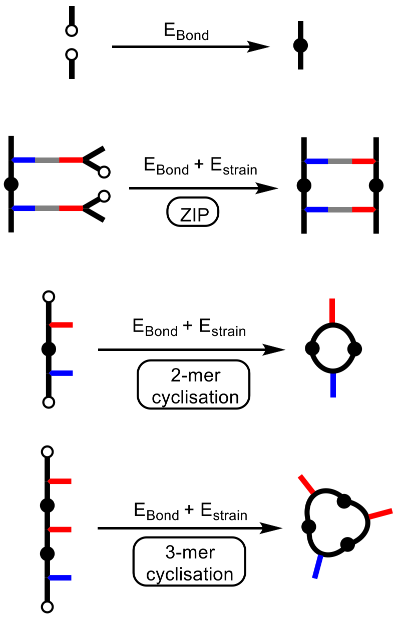

Scheme S9.

$$E_{Bond} = E_{product} - (2 \times E_{fragment}) \quad \text{(Eq.1)}$$

$$E_{strain} = E_{macrocycle} - E_{linear} - E_{bond} \quad \text{(Eq.2)}$$

1,4-bis(triazolyl)methylbenzene backbone (A)

Bond

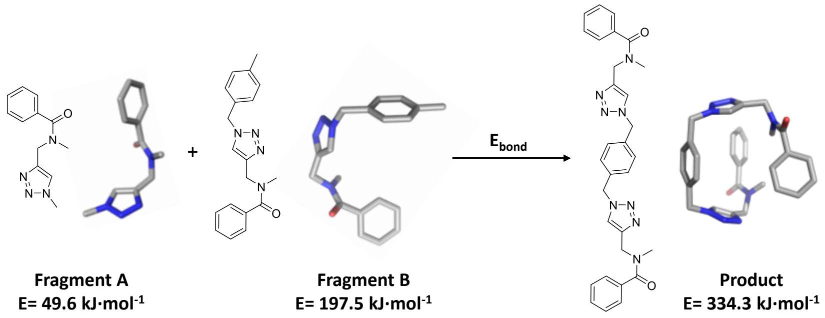

$E_{Bond} = E_{product} - (E_{fragmentA} + E_{fragmentB})$: 334.3 – (197.5 + 49.6) = **87.1 kJ·mol^{-1}**

ZIP

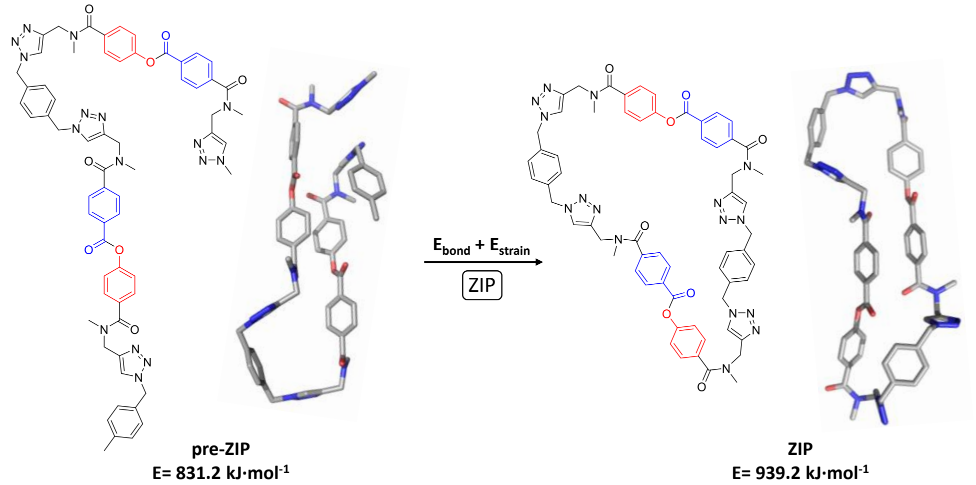

$E_{strain} = E_{ZIP} - E_{preZIP} - E_{bond}$ = 939.2 – 831.2 – 87.1 = **20.9 kJ·mol^{-1}**

2-mer macrocyclization

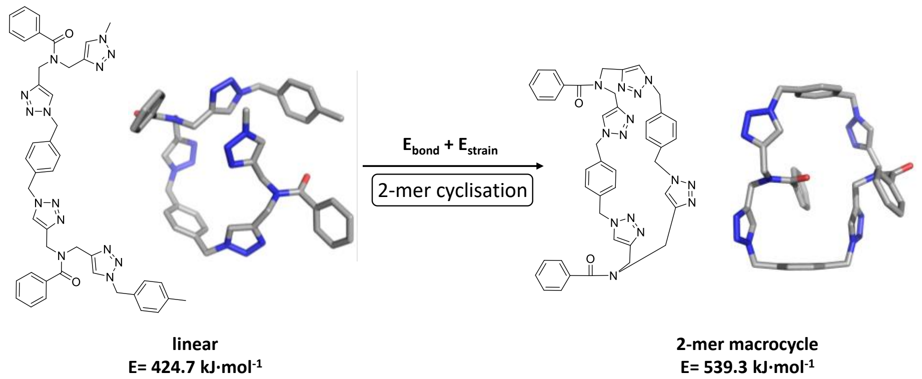

$E_{strain} = E_{macrocycle} - E_{linear} - E_{bond}$ = 539.3 – 424.7 – 87.1 = **27.5 kJ·mol^{-1}**

3-mer macrocyclization

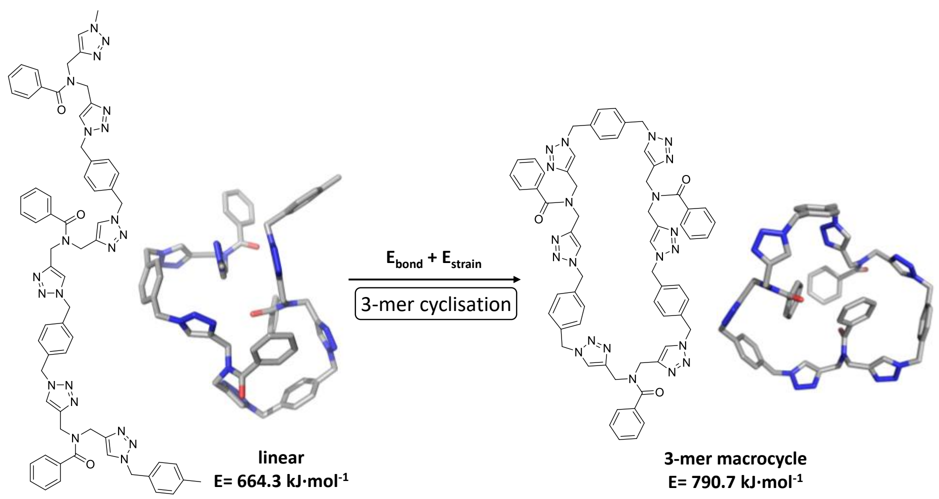

$E_{strain} = E_{macrocycle} - E_{linear} - E_{bond} = 790.7 - 664.3 - 87.1 = \mathbf{39.3\ kJ \cdot mol^{-1}}$

***p*-triazolylethynylbenzene backbone (B)**

Bond

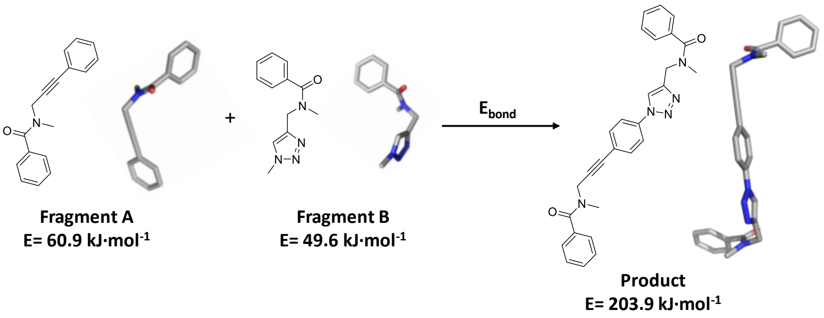

$E_{Bond} = E_{product} - (E_{fragmentA} + E_{fragmentB})$: $203.9 - (60.9 + 49.6) = \mathbf{93.4\ kJ \cdot mol^{-1}}$

ZIP

Parallel

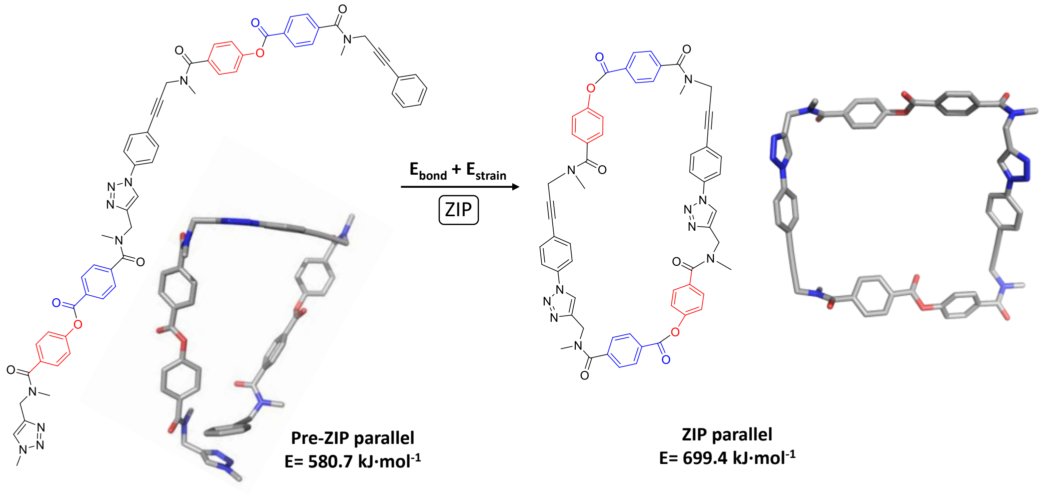

Antiparallel

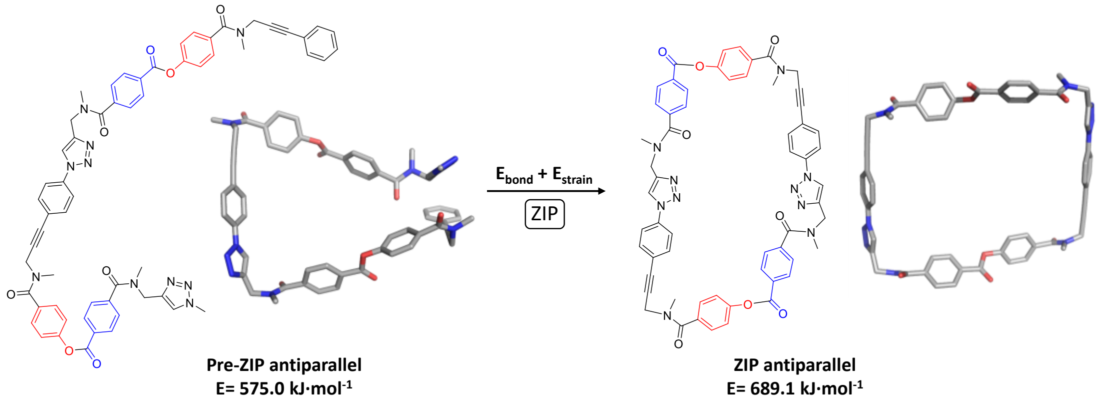

$E_{strain\ parallel} = E_{ZIP\ parallel} - E_{preZIP\ parallel} - E_{bond} = 699.4 - 580.7 - 93.4 = \mathbf{25.2\ kJ \cdot mol^{-1}}$

$E_{strain\ antiparallel} = E_{ZIP\ antiparallel} - E_{preZIP\ antiparallel} - E_{bond} = 689.1 - 575.0 - 93.4 = \mathbf{20.7\ kJ \cdot mol^{-1}}$

Average $E_{strain} = (25.2 + 20.7)/2 = \mathbf{23.0\ kJ \cdot mol^{-1}}$

2-mer macrocyclization

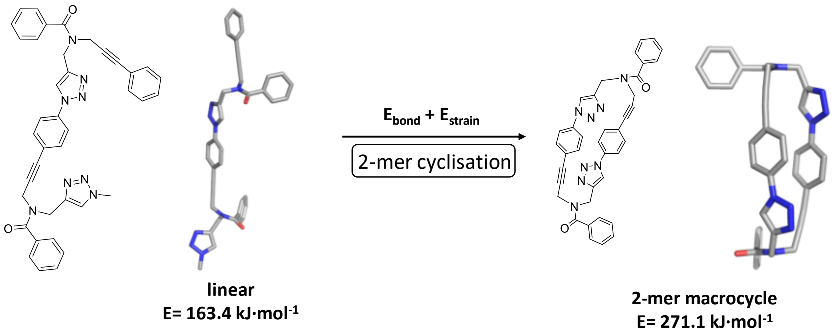

$E_{strain} = E_{macrocycle} - E_{linear} - E_{bond} = 271.1 - 163.4 - 93.4 = \mathbf{14.3\ kJ \cdot mol^{-1}}$

3-mer macrocyclization

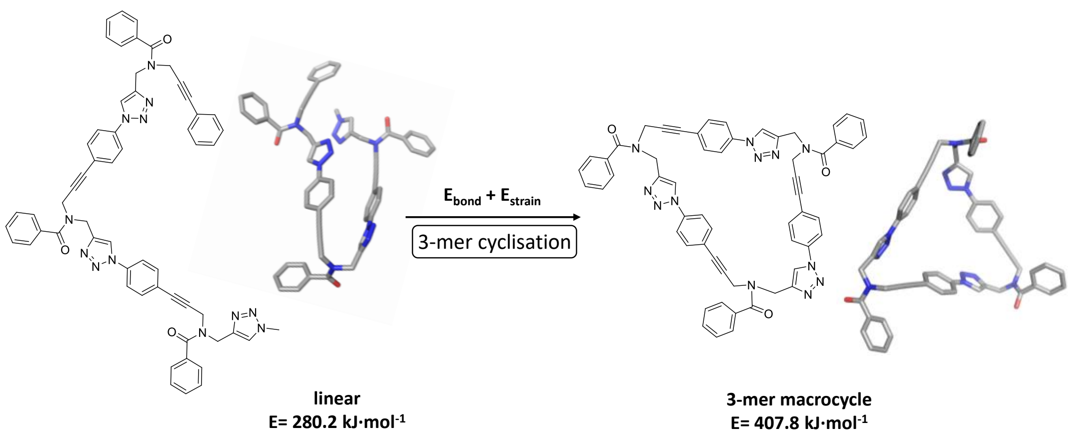

$E_{strain} = E_{macrocycle} – E_{linear} – E_{bond}$ = 407.8 – 280.2 – 93.4 = **34.2 kJ·mol^{-1}**

p-triazolylaniline backbone (C)

Bond

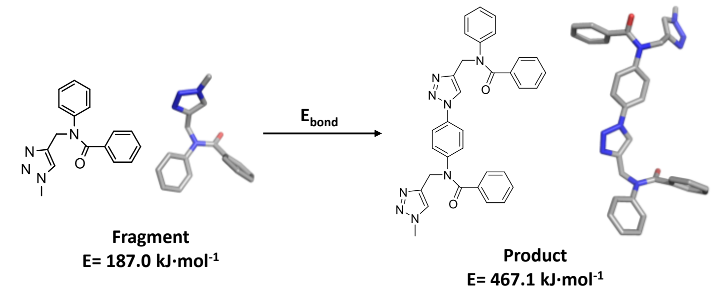

$E_{Bond} = E_{product} – (2xE_{fragment})$: 467.1 – (2 x 187.0) = **93.0 kJ·mol^{-1}**

ZIP

Parallel

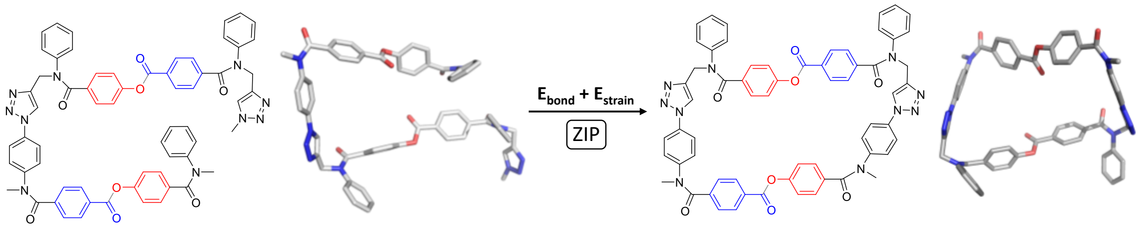

Pre-ZIP parallel
E= 1100.1 kJ·mol⁻¹

ZIP parallel
E= 1219.6 kJ·mol⁻¹

Antiparallel

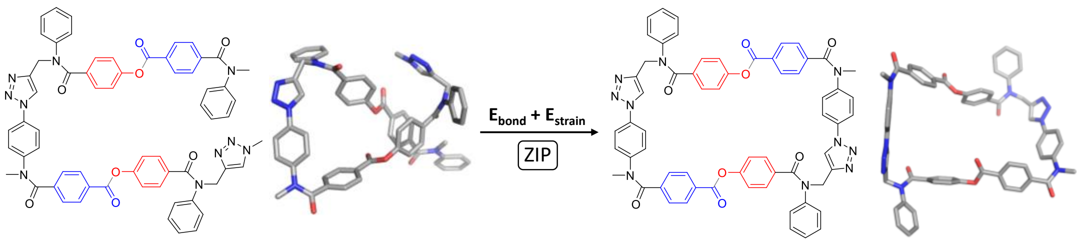

Pre-ZIP antiparallel
E= 1099.8 kJ·mol⁻¹

ZIP antiparallel
E= 1215.7 kJ·mol⁻¹

$E_{\text{strain parallel}} = E_{\text{ZIP parallel}} - E_{\text{preZIP parallel}} - E_{\text{bond}} = 1219.6 - 1100.1 - 93.0 = \mathbf{26.5\ kJ \cdot mol^{-1}}$

$E_{\text{strain antiparallel}} = E_{\text{ZIP antiparallel}} - E_{\text{preZIP antiparallel}} - E_{\text{bond}} = 1215.7 - 1099.8 - 93.0 = \mathbf{22.9\ kJ \cdot mol^{-1}}$

Average $E_{\text{strain}} = (26.5 + 22.9)/2 = \mathbf{24.7\ kJ \cdot mol^{-1}}$

2-mer macrocyclization

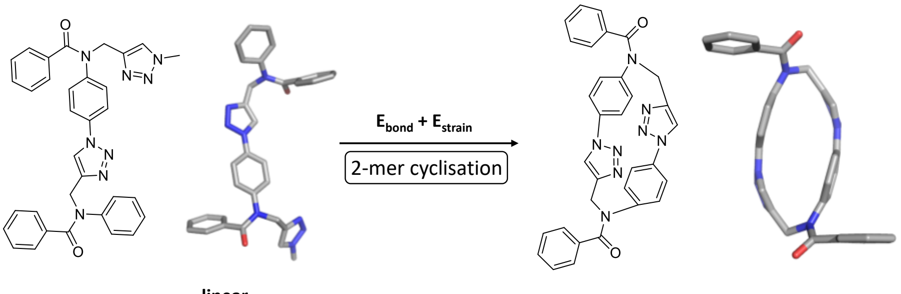

linear
E= 467.1 kJ·mol⁻¹

2-mer macrocycle
E= 671.8 kJ·mol⁻¹

$E_{\text{strain}} = E_{\text{macrocycle}} - E_{\text{linear}} - E_{\text{bond}} = 671.8 - 467.1 - 93.0 = \mathbf{111.7\ kJ \cdot mol^{-1}}$

3-mer macrocyclization

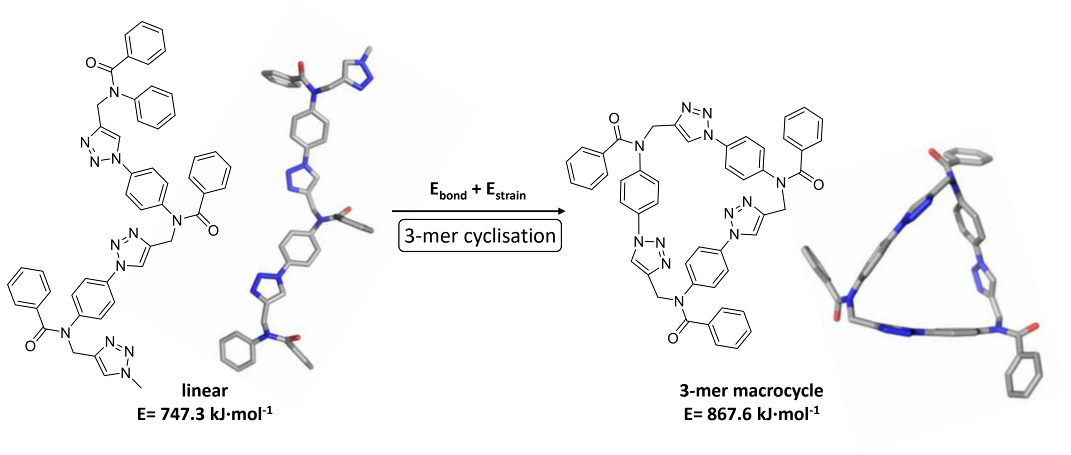

$E_{strain} = E_{macrocycle} - E_{linear} - E_{bond} = 867.6 - 747.3 - 93.0 = \mathbf{27.3\ kJ{\cdot}mol^{-1}}$

Ring strain calculations for 2-mer macrocycle attached to the template strand.

1,4-bis(triazolyl)methylbenzene backbone (A)

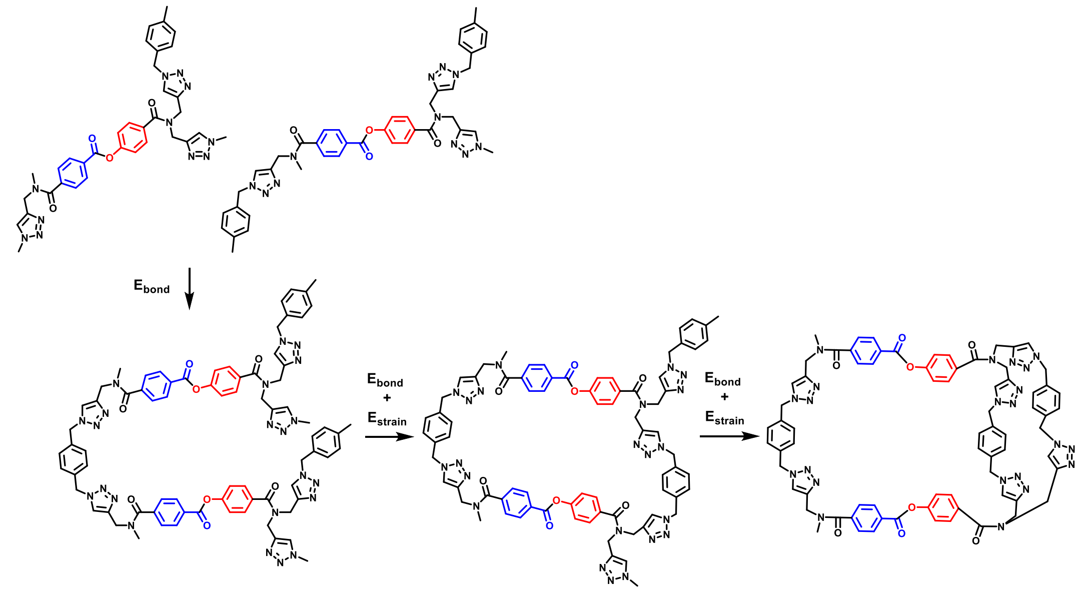

p-triazolylethynylbenzene backbone (B)

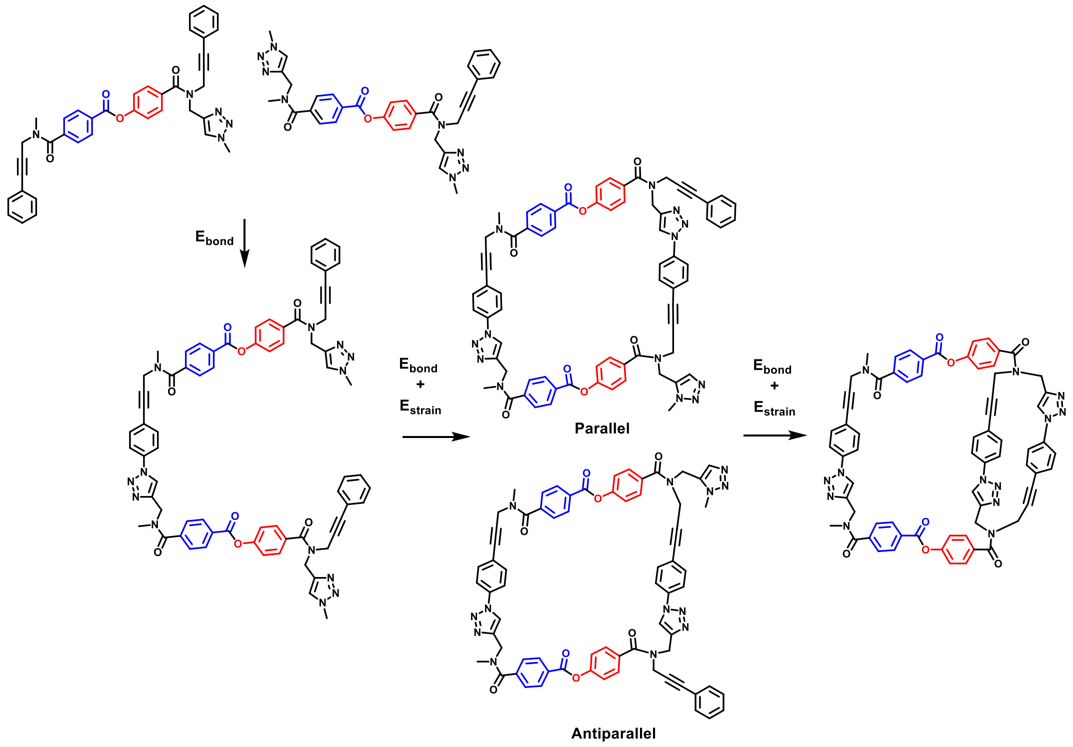

p-triazolylaniline backbone (C)

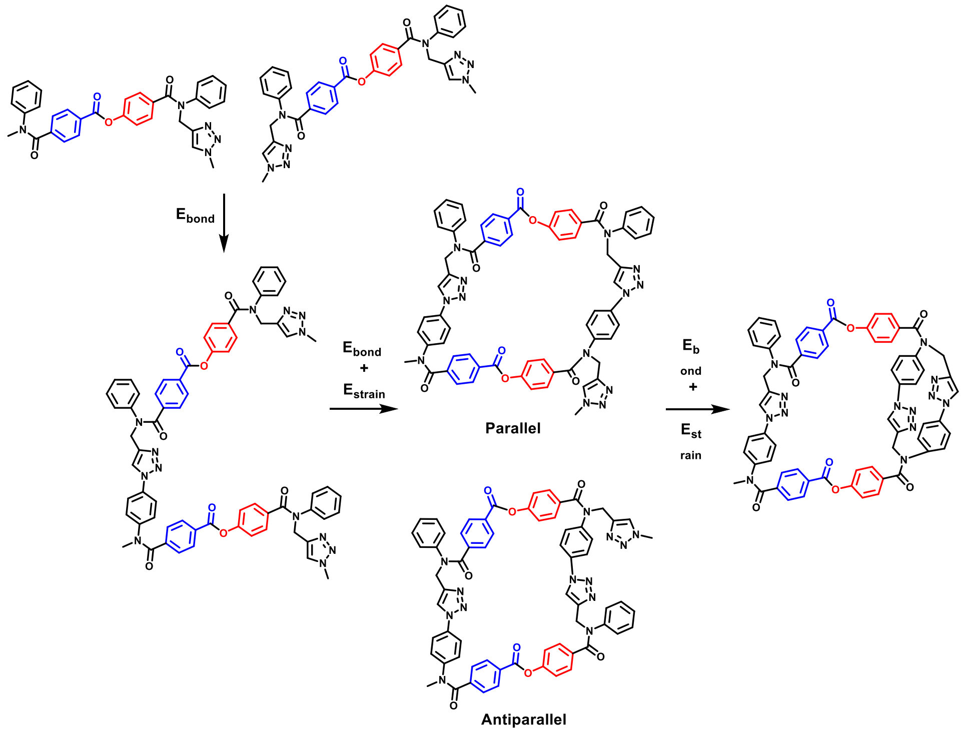

In the same way as in the previous section, these structures were used to perform molecular mechanics conformational searches with the MMFFs force-field and $CHCl_3$ solvation, the energies of the lowest conformations were used to calculate the ring strain for the

macrocyclization reaction. Figure S11 compares the ring strain energies for macrocyclization reaction for the simplified structures used in the previous section and those shown above.

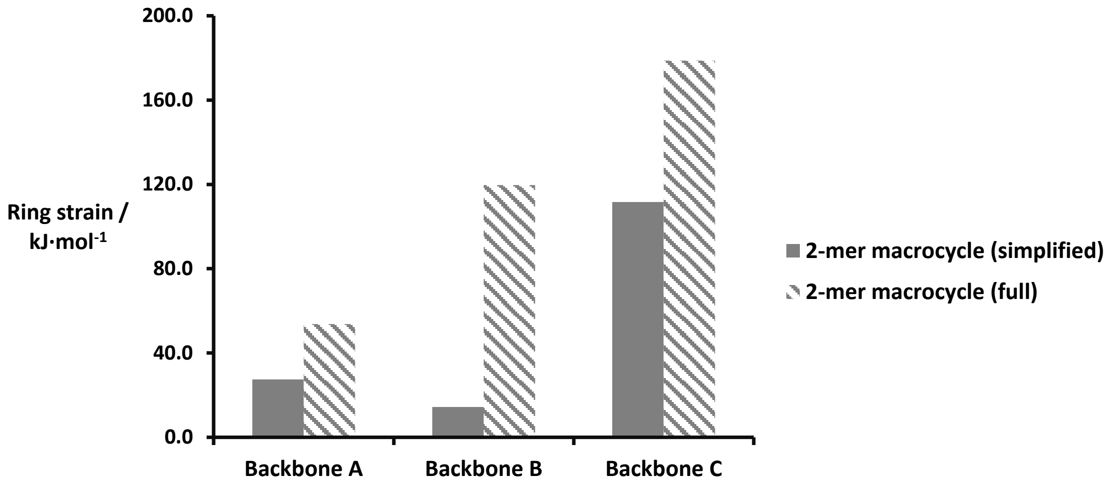

Figure S11. Comparison of the calculated ring strain energies for the macrocyclization reaction for the structures shown in the previous section (simplified structures) and those shown in the previous page (full structures)

References

[S1] D. Núñez-Villanueva, M. Ciaccia, C. A. Hunter, *RSC Adv.*, 2019, **9**, 29566.

[S2] D. Núñez-Villanueva, M. Ciaccia, G. Iadevaia, E. Sanna, C. A. Hunter. *Chem. Sci.* **2019**,*10*, 5258.

[S3] D. Núñez-Villanueva, M. A. Jinks, J. Gómez Magenti, C. A. Hunter. *Chem. Commun.* **2018**, *54*, 10874.

[S4] J. R. Thomas, X. Liu, P. J. Hergenrother. *J. Am. Chem. Soc.* **2005**, *127*, 12434.

[S5] T. Slagbrand, A. Volkov, P. Trillo, F. Tinnis and H. Adolfsson. *ACS Catal.* **2017**, *7*, 1771.

[S6] J. J. Gassensmith, L. Barr, J. M. Baumes, A. Paek, A. Nguyen and B. D. Smith. *Org. Lett.* **2008**, *10*, 3343.

[S7] Schrödinger Release 2022-1: MacroModel, Schrödinger, LLC, New York, NY, 2022 (version 13.1.141).

[S8] The PyMOL Molecular Graphics System (open-source PyMOL). Version 2.3.0a0. Schrödinger, LLC
